# Supplementary material for: Development of a 1,3a,6a-triazapentalene derivative as a compact and thiol-specific fluorescent labeling reagent
Source: Commun Chem. 2020 Jan 9;3:6. doi: 10.1038/s42004-019-0250-0 (PMC9812263; doi:10.1038/s42004-019-0250-0)
Supplement: Supplementary file 2 — Supplementary Information [file 42004_2019_250_MOESM2_ESM.pdf]

• Supplementary Tables

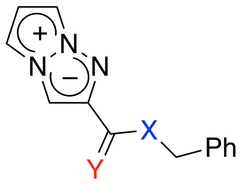

functional goup	X	Y	half life (min)
alkyl: **1a**	CH_2	H, H	8
amide: **1b**	NH	O	15
ester: **1c**	O	O	45
ketone: **1d**	CH_2	O	90

Supplementary Table 1 | Stability comparison of various functional groups at the C2 position. The photo stability test was conducted with 1,3a,6a-triazapentalene analogs possessing various functional groups such as alkyl (**1a**), amide (**1b**), ester (**1c**), and ketone (**1d**) at the C2-position. The detail was described at the Supplementary Notes 1.

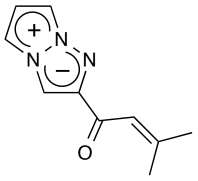

TAP-VK1 (2a)

	C_6H_6	CH_2Cl_2	Acetone	MeOH	MeCN	DMF	H_2O
λ_{abs}^{max} (nm)	**406**	**409**	**407**	**406**	**408**	**414**	**416**
λ_{em}^{max} (nm)	**554**	**574**	**614**	**–**	**–**	**–**	**–**
Φ_F	0.039	0.023	0.034	–	–	–	–

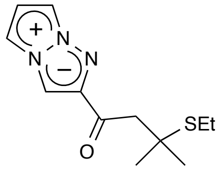

3a

	C_6H_6	CH_2Cl_2	Acetone	MeOH	MeCN	DMF	H_2O
λ_{abs}^{max} (nm)	**385**	**390**	**384**	**385**	**384**	**390**	**396**
λ_{em}^{max} (nm)	**503**	**529**	**525**	**–**	**540**	**530**	**–**
Φ_F	0.36	0.19	0.056	–	0.052	0.27	–

Supplementary Table 2 | Fluorescence solvatochromism of TAP-VK1 (2a) and 1,4-adduct (3a). The synthesized triazapentalenes **2a** and **3a** exhibit positive solvatofluorochromism. The fluorescence maximum and fluorescence quantum yields of **2a** and **3a** in different solvents are shown in Supplementary Table 2.

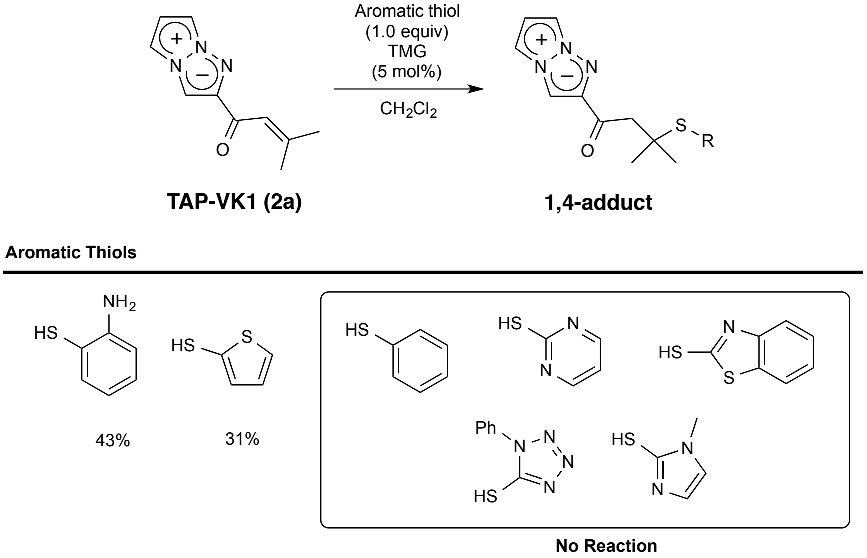

Supplementary Table 3 | Results of 1,4-addition reaction of aromatic thiols with TAP-VK1 (2a). Although most aromatic thiols did not react with TAP-VK1 (**2a**), only 2-aminobenzenethiol and thiophene-2-thiol reacted to give labeled products in 43% and 31% isolated yield, respectively.

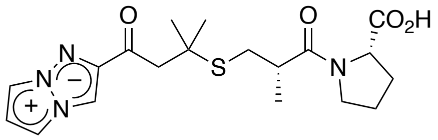

Captopril-TAP-VK1 (3o)

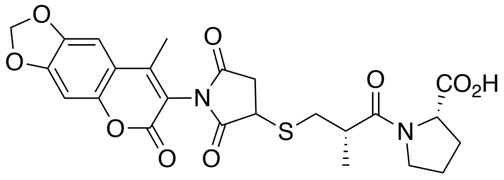

Captopril-Coumarin (8o)

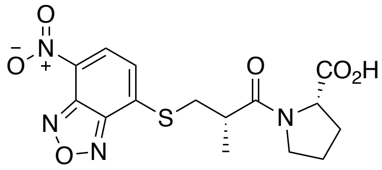

Captopril-NBD (9o)

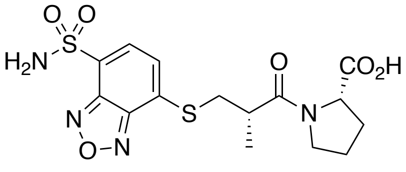

Captopril-ABD (10)

Compound	Solvent	λ_{abs}^{max} (nm)	λ_{em}^{max} (nm)	Φ_F	ε ($dm^3\ mol^{-1}\ cm^{-1}$)
Captopril-TAP-VK1 (3o)	CH_2Cl_2	395	506	0.014	1.15×10^3
Captopril-Coumarin (8o)	CH_2Cl_2	352	414	0.64	9.16×10^3
Captopril-NBD (9o)	CH_2Cl_2	416	497	0.00012	1.53×10^4
Captopril-ABD (10o)	MeOH	385	513	0.017	6.81×10^3

Supplementary Table 4 | Comparison of the fluorescent properties of Captopril derivatives conjugated with TAP-VK1 (2a), Coumarin-maleimide (8), NBD-Cl (9), ABD-F (10). The absorbance and fluorescence maximum, fluorescence quantum yields and the molar extinction coefficient of Captopril-VK1 (**3o**), Captopril-Coumarin (**8o**), Captopril-NBD (**9o**), and Captopril-ABD (**10o**) are shown in Supplementary Table 4.

• Supplementary Figures

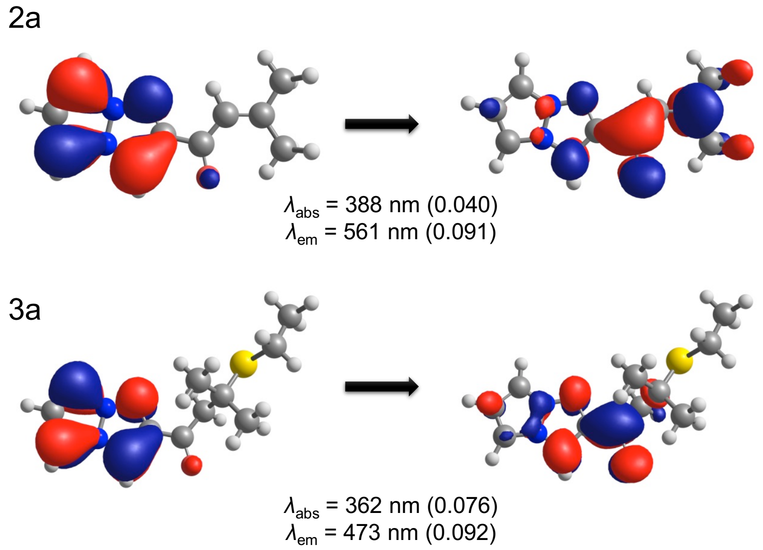

Supplementary Figure 1 | Computational Studies on the fluorescence change after the addition of thiols. The time-dependent density functional theory (TD-DFT) method was conducted for the explanation of the fluorescence difference between TAP-VK1 (**2a**) and thiol adduct **3a**. The detail was described at the Supplementary Notes 2.

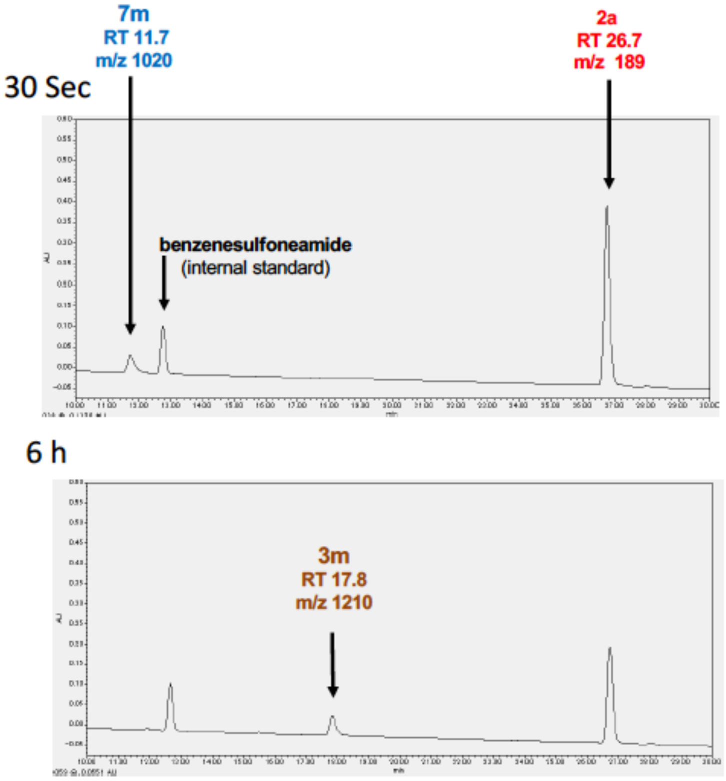

Supplementary Figure 2 | HPLC analysis of the labeling reaction of 6m with 2a. Reverse-phase HPLC (COSMOSIL 5C18-AR-Ⅱ, 4.6ID x 250 mm; Solvent A: [10 mM NH_4OAc buffer]; Solvent B: [MeCN]; Gradient: 5% [B] to 50% [B] from 0 to 30 min; Monitored by UV at 220 nm). The peak of 17.8 min was confirmed as **3m** by a tandem mass spectrometry (MS/MS) analysis (vide infra). There were no peaks other than those for the internal standard, the starting material (**7m**), the labeled product (**3m**), and excess labeling reagent (**2a**).

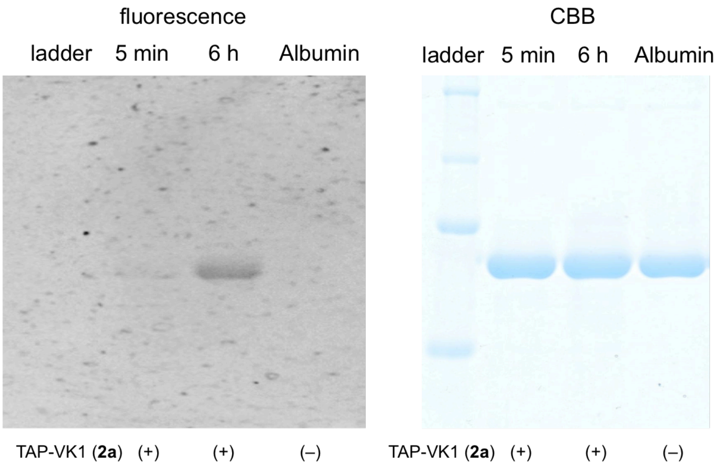

Supplementary Figure 3 | Bioconjugation of HSA with TAP-VK1 (2a). The reaction mixture was analysed using SDS-PAGE in 10% polyacrylamide gels. Fluorescence of the band was detected using a LAS-4000mini (λ_{ex} = 460 nm, λ_{em} >515 nm). For the visualization of all proteins, CBB (Coomassie Brilliant Blue) was employed**.** The band of HAS showed fluorescence derived from TAP-VK1, and no other fluorescent band was observed.

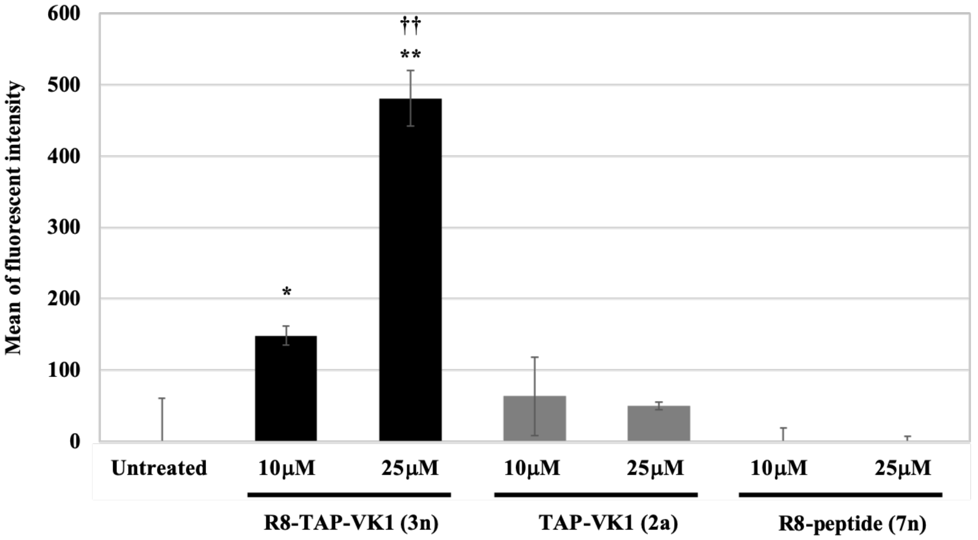

Supplementary Figure 4 | Concentration-dependence of R8-TAP-VK1 (3n), TAP-VK1 (2a) and R8-peptide (7n). The fluorescence intensities of **3n** inside the cells exhibited concentration-dependence, whereas treatments with a solution of TAP-VK1 did not result in concentration-dependence. Error bars represent the standarddeviation of three repeats. *: P < 0.05 (vs Untreated: Student's t-test), **: P < 0.01 (vs Untreated: Student's t-test), ††: P < 0.01 (vs TAP-VK1: Student's t-test).

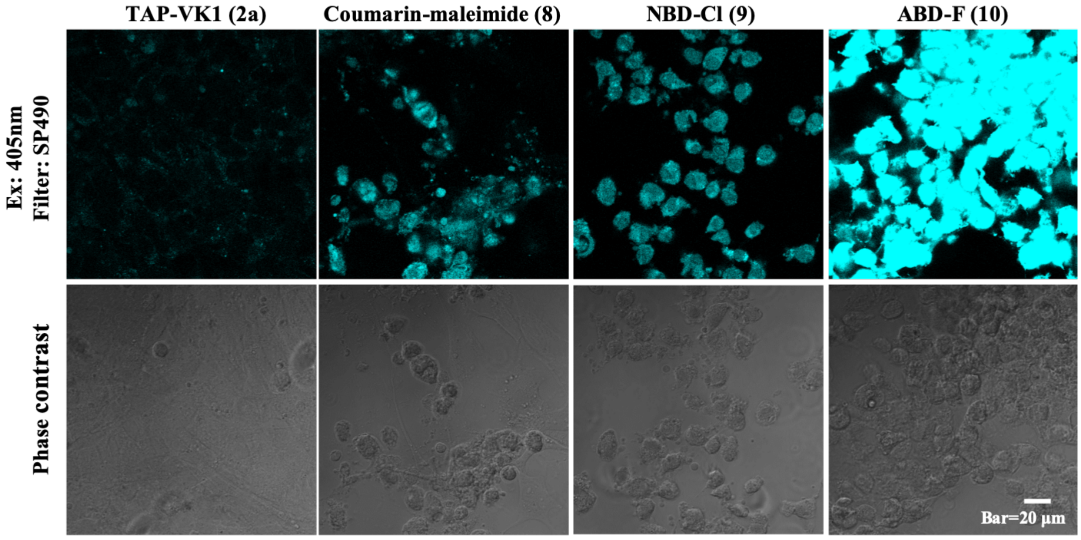

Supplementary Figure 5 | Fluorescence and phase contrast images of high concentration (50 µM) treatment of TAP-VK1 (2a), Coumarin-maleimide (8), NBD-Cl (9), and ABD-F (10) to mouse brain-derived vascular endothelial cell (MBEC4). Mouse brain-derived vascular endothelial cell (MBEC4) was treated with 50 µM of TAP-VK1 (**2a**), Coumarin-maleimide (**8**), NBD-Cl (**9**), and ABD-F (**10**) and incubated for 24 h. The fluorescence distribution of each compounds was observed by confocal microscopy (ZEISS LSM700; Carl Zeiss) in a fluorescence image with excitation and emission wavelengths of 405 and 490 nm. **2a** was not much taken into the MBEC4. On the other hand, **8**, **9** and **10** seemed to be absorbed on the cell surface and the cell damage was caused by these three reagents.

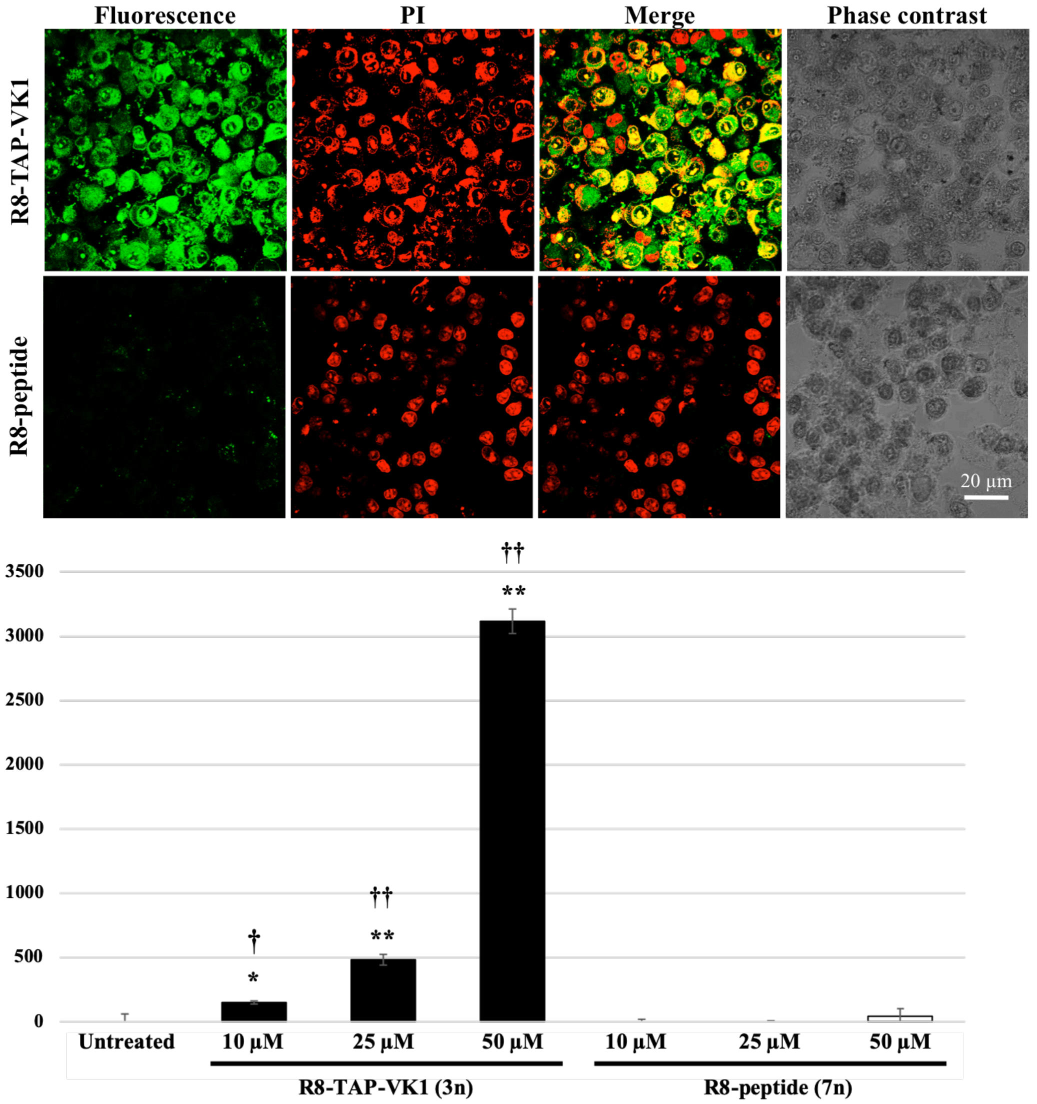

Supplementary Figure 6 | Fluorescence and phase contrast images of high concentration (50 μM) treatment of R8-TAP-VK1 (3n) and R8-peptide (7n) to A549 cell. A549 cell was treated with 50 μM of R8-TAP-VK1 (**3n**) or R8-peptide (**7n**) and incubated for 24 h, and fluorescence and phase contrast images were obtained. R8-TAP-VK1 (3n): Ex/Em = 405 nm/490-555 nm. Propidium Iodide (PI): Ex/Em = 555 nm/560 nm. Treatment with a 50 μM solution of **3n** caused cell damage, although much higher fluorescence intensity inside the cells was observed. Treatment with the 50 μM solution of the R8 peptide **7n** also showed similar cell damage, while a similar concentration of TAP-VK1 (**2a**) solution did not exhibit cytotoxicity to cells. The cell damage caused by the high-concentration solution including **3n** was thus due to the presence of the R8 peptide. In addition, it has already been clarified that the TAP scaffold does not show cytotoxicity in previous reports. [1,2] Error bars represent the standarddeviation of three repeats. *: $P < 0.05$ (vs Untreated: Student's t-test), **: $P < 0.01$ (vs Untreated: Student's t-test), †: $P < 0.05$ (vs R8-peptide: Student's t-test). ††: $P < 0.01$ (vs R8-peptide: Student's t-test).

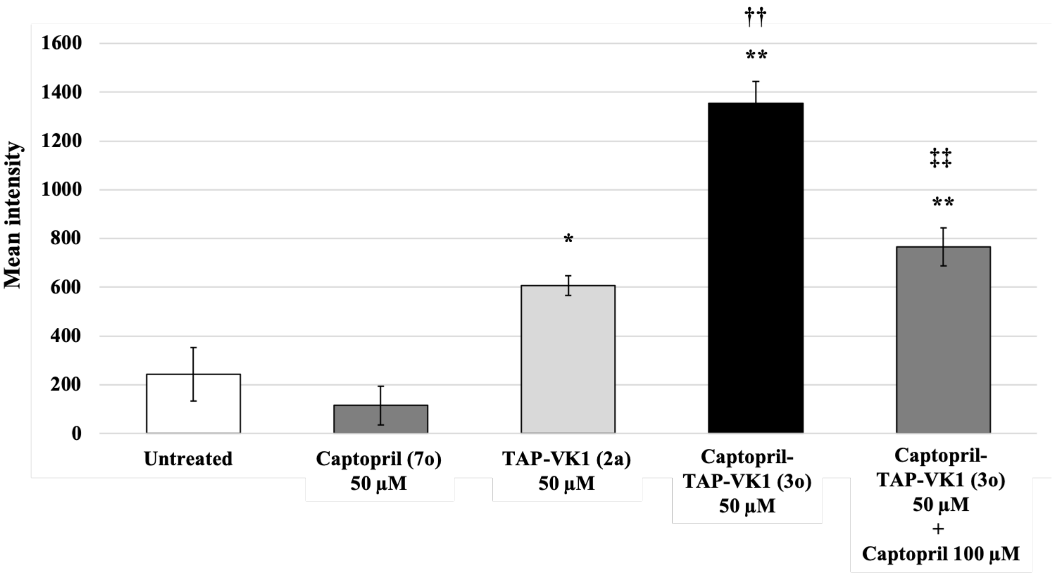

Supplementary Figure 7 | Intracellular fluorescence intensities of vascular endothelial cells that were treated by captopril (7o), TAP-VK1 (2a), VK1-Captopri; (3o) and the mixture of 7o and 3o (2 : 1). The interior of cells treated by **3o** showed higher fluorescence than that of **2a** and **7o**. The coexistence of **7o** reduced the fluorescence intensity of **3o**, suggesting that **7o** competed with **3o** for binding to ACE. Error bars represent the standarddeviation of three repeats. *: P < 0.05 (vs Untreated: Student's t-test), **: P < 0.01 (vs Untreated: Student's t-test), ††: P < 0.01 (vs TAP-VK1: Student's t-test), ‡‡: P < 0.01 (vs Captopril-TAP-VK1: Student's t-test).

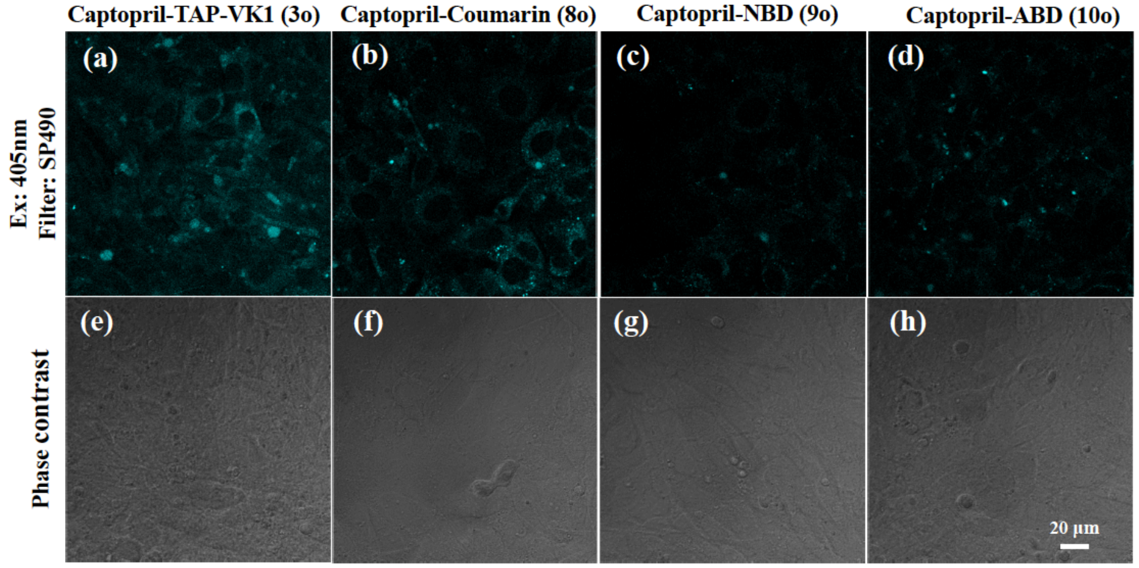

Supplementary Figure 8 | Fluorescence and phase contrast images of high concentration (50 μM) treatment of Captopril-TAP-VK1 (3o), Captopril-Coumarin (8o), Captopril-NBD (9o) and Captopril-ABD (10o) to mouse brain-derived vascular endothelial cell (MBEC4). Mouse brain-derived vascular endothelial cell (MBEC4)[3] was treated with 50 μM of Captopril-TAP-VK1 (**3o**), Captopril-Coumarin (**8o**), Captopril-NBD (**9o**) and Captopril-ABD (**10o**) and incubated for 24 h**.** The fluorescence distribution of each compounds was observed by confocal microscopy (ZEISS LSM700; Carl Zeiss) in a fluorescence image with excitation and emission wavelengths of 405 and 490 nm (panels (a)-(d)). The uptake of **3o** and **8o** were observed by monitoring their fluorescence (panels (a) and (b)). On the other hand, the sufficient fluorescence of **9o** and **10o** were not observed (panels (c) and (d)). Panels (e)-(h) displays phase contrast images at the each condition. Error bars represent the standarddeviation of three repeats. ***: $P < 0.001$ (vs Captopril-TAP-VK1 (**3o**): Student's t-test).

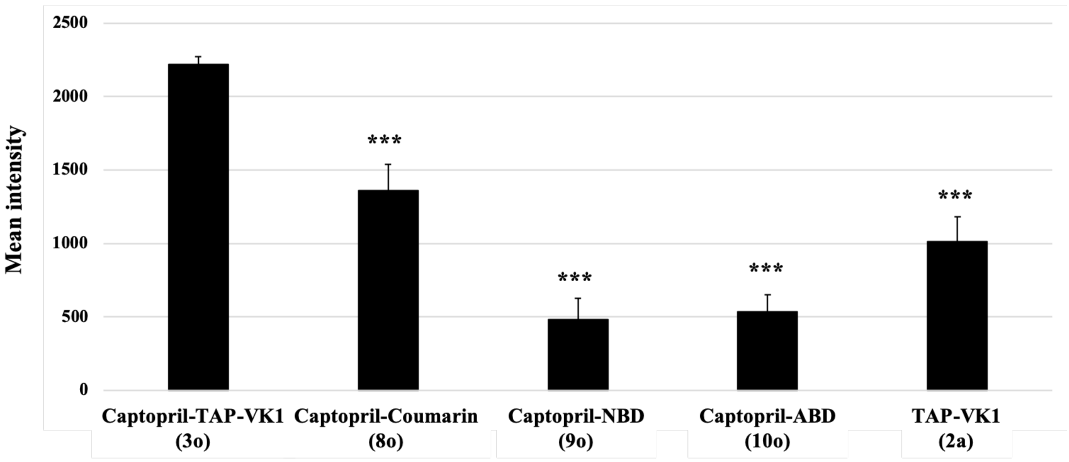

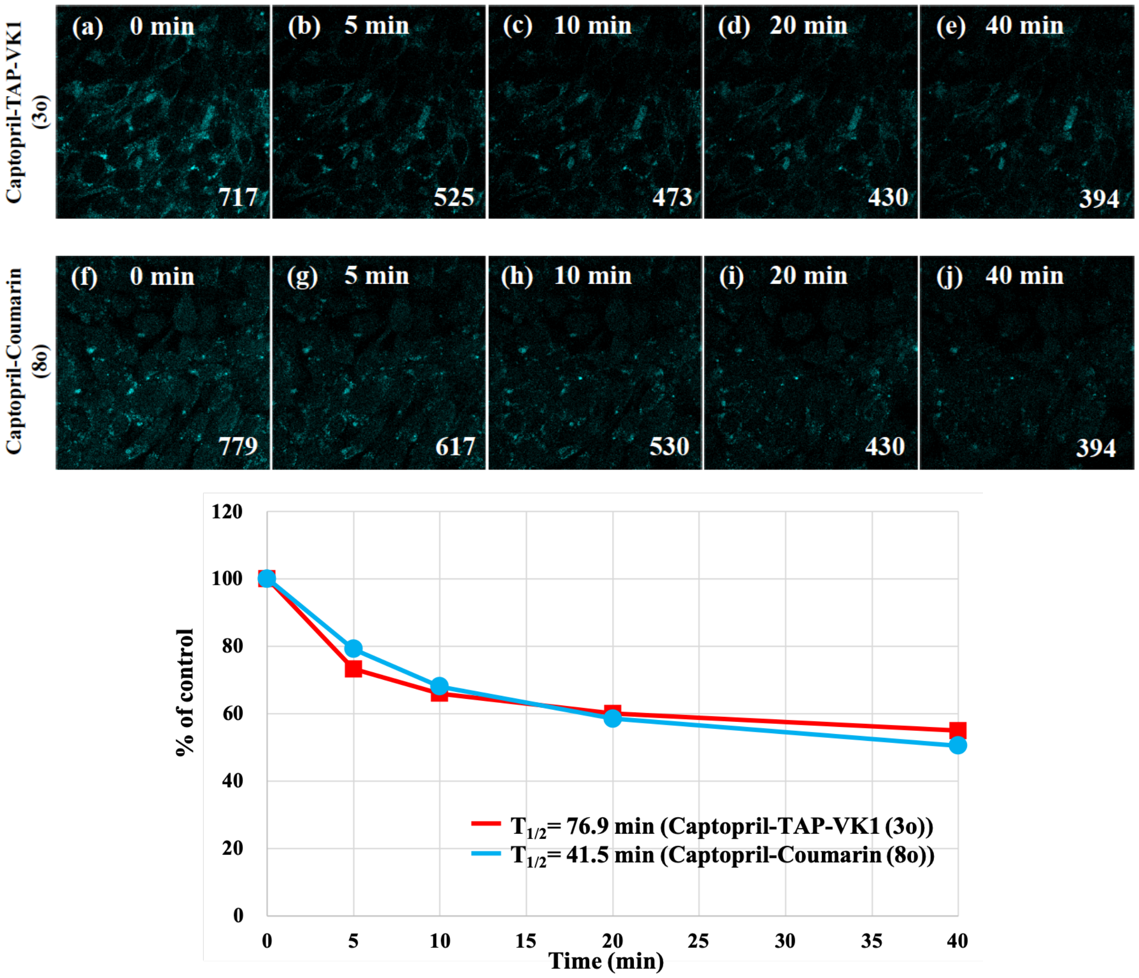

Supplementary Figure 9 | Photostability test of Captopril-TAP-VK1 (3o) and Captopril-Coumarin (8o). Photobleaching experiment with **3o** and **8o** was conducted on the mouse brain-derived vascular endothelial cells (MBEC4) which expressed ACE. After treatment of the MBEC4 with 50 μM of **3o** and **8o** respectively, the cells were exposed to the continuous excitation laser at 405 nm (5 mW Diode Laser) and measured the fluorescence intensity of **3o** and **8o** in the cells at 0 min (panels (a) and (j)), 5 min (panels (b) and (g)), 10 min (panels (c) and (h)), 20 min (panels (d) and (i)) and 40 min (panels (e) and (j)), respectively. Ex/Em = 405 nm/490 nm. The white numbers of the pictures (panels (a)-(j)) display the fluorescence intensity. The fluorescence half-life time ($T_{1/2}$) was obtained from the graph summarizing the reduced fluorescence intensity of **3o** and **8o** over time.

• Supplementary Notes

Supplementary Note 1 | Details of stability comparison of various functional groups at the C2 position. Solutions of 1,3a,6a-triazapentalene analogs possessing various functional groups such as alkyl (**1a**), amide (**1b**), ester (**1c**), and ketone (**1d**) at the C2-position in $CDCl_3$ were exposed to intense light from a high-pressure mercury lump (400 W), and their half-lives were measured by comparing the ^{1}H NMR integration values between **1** and dichloroethane as an internal standard. The ketone analog **1d** exhibited the longest half-life (90 min) and was thus the most stable. 1,3a,6a-triazapentalene derivatives **1a-d** were synthesized according to the single-step synthesis of 1,3a,6a-triazapentalenes developed in our laboratory (see chemical synthesis section).

Supplementary Note 2 | Details of supplementary Fig.1| Computational Studies on the fluorescence change after the addition of thiols. Dominant natural transition orbital (NTO) pairs for the S_1 transition of **2a** and **3a** in dichloromethane, where the "hole" is on the left and the "particle" on the right. The numbers in parentheses represent oscillator strength for $S_0 \rightarrow S_1$ transition.

With the discovery of substantial fluorescence change from **2a** to **3a**, quantum chemical calculations by the time-dependent density functional theory (TD-DFT) method were performed to elucidate the shift of the emission maximum. Supplementary Figure 1 shows the relevant natural transition orbitals (NTO) pairs involved in the photo-excitation to the first singlet excited state (S_1), and it is characterized by the HOMO → LUMO transition. As seen, the transition involves the charge transfer from the 1,3a,6a-triazapentalene skeleton to the 2-substituents. The vertical excitation energies are calculated to be 3.19 eV (388 nm) and 3.47 eV (362 nm) for **2a** and **3a**, respectively, and they are in good agreement with experimental results of 409 nm (**2a**) and 389 nm (**3a**). The emission energies are also evaluated at the equilibrium structures in the S_1 state, $(S_1)_{min}$, and they are calculated to be 2.21 eV (561 nm) and 2.62 eV (473 nm) for **2a** and **3a**, respectively, which are also in fairly good agreement with the experiments (574 nm for **2a** and 521 nm for **3a**). The observed smaller Stokes shift of **3a** than **2a** is attributed to the decreased charge-transfer character in **3a**, where the electronic conjugation of the 2-substituents is terminated in **3a** (Supplementary Figure 1).

• Supplementary Methods

General Procedure

All the reactions were carried out in a round-bottomed flask with an appropriate number of necks and side arms connected to a three-way stopcock and/or a rubber septum cap under an argon atmosphere. All vessels were first evacuated by rotary pump and then flushed with argon prior to use. Solution and solvent were introduced by hypodermic syringe through a rubber septum. During the reaction, the vessel was kept under a positive pressure of argon. Dry THF was freshly prepared by distillation from benzophenone ketyl before use. Anhydrous CH_2Cl_2, DMF, ethanol, MeCN, methanol, pyridine and toluene were purchased from Kanto Chemical Co. Inc. Infrared (IR) spectra were recorded on JASCO FT/IR-4100 spectrophotometer using 5 mm KBr plate. Wavelengths of maximum absorbance are quoted in cm^{-1}. ^{1}H-NMR spectra were recorded on a JEOL ECA–400 (400 MHz), Bruker AV-400N (400 MHz), and Bruker AV–500 (500 MHz) in $CDCl_3$, *d*–MeCN and D_2O. Chemical shifts are reported in part per million (ppm), and signal are expressed as singlet (s), doublet (d), triplet (t), quartet (q), multiplet (m) and broad (br). ^{13}C-NMR spectra were recorded on a JEOL ECA–400 (100 MHz), Bruker AV–400N (100 MHz) and Bruker AV–500 (125 MHz) in $CDCl_3$, C_6D_6, CD_3CN and D_2O. Chemical shifts are reported in part per million (ppm). High resolution mass (HRMS) spectra were recorded on a Thermo Scientific Exactive. High performance liquid chromatography (HPLC) was recorded on a HITACHI D-2500 Chromato-Integrater. All melting points were measured with Yanaco MP-500D and BUCHI 535 melting point apparatuses. Absorption spectra were recorded on a JASCO V-600 spectrometer and corrected fluorescence spectra were recorded on a JASCO FP-8200 spectrofluorometer. Sample solutions were degassed thoroughly by purging with an Ar gas stream for 30 min prior to the experiments and then sealed in their cells. Fluorescence quantum yields were estimated by using 9,10-diphenylanthracene (9,10-DPA) in cyclohexane ($\Phi_F = 0.91$) or rhodamine B in ethanol ($\Phi_F = 0.94$) as a standard.Analytical thin layer chromatography (TLC) was performed using 0.25 mm E. Merck Silica gel (60F-254) plates. Reaction components were visualized phosphomolybdic acid or ninhydrin or *p*-anisaldehyde in 10% sulfuric acid in ethanol. Kanto Chem. Co. Silica Gel 60N (particle size 0.040–0.050 mm) was used for column chromatography. High-resolution images of cells were obtained using a ZEISS LSM700 (Carl Zeiss) confocal microscope.

Experimental Procedures

General procedure of the click reaction

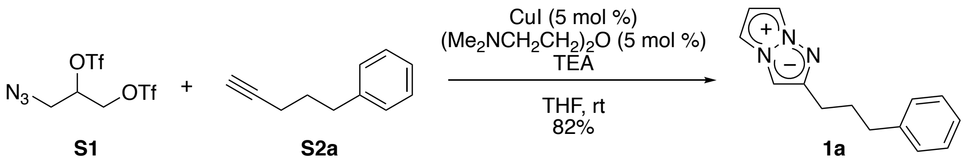

2-(3-phenylpropyl)pyrazolo[1,2-*a*][1,2,3]triazol-8-ium-1-ide (1a): To a solution of bis[2-(*N*,*N*-dimethylaminoethyl)]ether (19.0 μL, 0.10 mmol) in THF (10 mL) was added copper(I) iodide (19 mg, 0.10 mmol) at room temperature. The mixture was stirred until homogeneous, and 1.1 mL (0.11 mmol of ligand·copper complex) of resulting mixture was transferred to the flask containing azide **S1** (100 mg, 0.26 mmol) in THF (25 mL). To the mixture were added TEA (166 μL, 1.19 mmol) and alkyne **S2a** (37.1μL, 0.24 mmol) at room temperature, successively. The mixture was stirred for 3 h and concentrated under reduced pressure. The residue was purified by silica gel column chromatography (EtOAc/hexane = 1/4, 1% Et_3N was contained) to give **1a** (44.1 mg, 0.196 mmol, 82%) as a brown oil. ^{1}H NMR ($CDCl_3$, 400 MHz): δ 7.33 (d, J = 2.4 Hz, 1H), 7.30-7.26 (m, 2H), 7.21-7.01 (m, 3H), 7.01 (d, J = 2.8 Hz, 1H), 6.90 (s, 1H), 6.54 (t, J = 2.8 Hz, 1H), 2.72 (t, J = 7.8 Hz, 2H), 2.68 (t, J = 7.8 Hz, 2H), 2.04 (quint, J = 7.8 Hz, 2H). ^{13}C NMR ($CDCl_3$, 100 MHz): δ 148.8, 142.0, 128.5, 128.3, 125.7, 107.9, 101.7, 100.0, 94.5, 35.4, 30.8, 26.2. IR (KBr): 3154, 2934, 2857, 1495, 1434, 1377, 1240, 1132, 700, 660 cm^{-1}. HRMS (ESI): m/z $[M+H]^+$ calcd for $[C_{14}H_{16}N_3]^+$ 226.1344, found 226.1338. UV/Vis (CH_2Cl_2): λ_{max} (log ε) = 285 (3.61) nm. FL (CH_2Cl_2): λ_{max} = 294 nm.

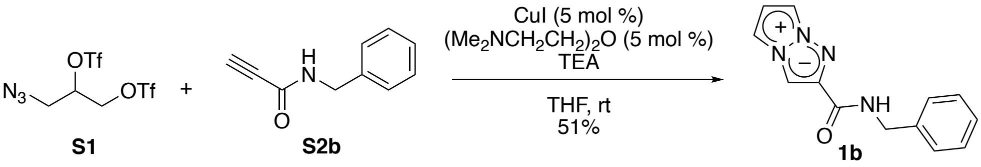

2-(benzylcarbamoyl)pyrazolo[1,2-*a*][1,2,3]triazol-8-ium-1-ide (1b): brown solid. Mp 77 °C (dec.) (recrystallized from EtOAc). ^{1}H NMR ($CDCl_3$, 400 MHz): δ 7.67 (d, J = 1.2 Hz, 1H), 7.36-7.28 (m, 6H), 7.19 (d, J = 3.0 Hz, 1H), 7.12 (br s, 1H), 6.70 (t, J = 3.0 Hz, 1H), 4.64 (d, J = 6.0 Hz, 2H). ^{13}C NMR ($CDCl_3$, 100 MHz): δ 160.8, 142.4, 137.9, 128.7, 127.8, 127.5, 110.2, 102.2, 101.8, 98.6, 43.2. IR (KBr): 3309, 3151, 2923, 2360, 1660, 1574, 1533, 1258, 698 cm^{-1}. HRMS (ESI) m/z $[M+H]^+$ calcd for $[C_{13}H_{13}N_4O]^+$ 241.1089, found 241.1085. UV/Vis (CH_2Cl_2): λ_{max} (log ε) = 327 (3.29), 281 (3.97) nm. FL (CH_2Cl_2): λ_{max} = 420 nm. Φ_F = 0.011 (reference to 9,10-DPA; excited at 350 nm).

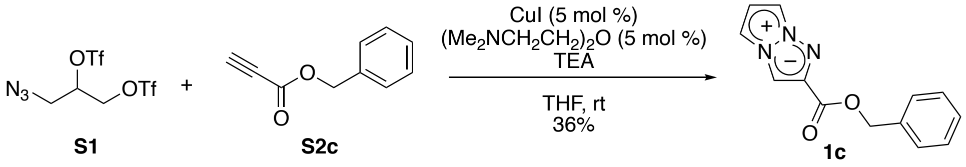

2-((benzyloxy)carbonyl)pyrazolo[1,2-*a*][1,2,3]triazol-8-ium-1-ide (1c): pale yellow solid. Mp 88-89 °C (recrystallized from EtOAc). ^{1}H NMR ($CDCl_3$, 400 MHz): δ 7.68 (d, *J* = 1.2 Hz, 1H), 7.47-7.45 (m, 3H), 7.40 – 7.31 (m, 3H), 7.19 (d, *J* = 3.0 Hz, 1H), 6.73 (t, *J* = 3.0 Hz, 1H), 5.40 (s, 2H); ^{13}C NMR ($CDCl_3$, 100 MHz): δ 161.4, 139.1, 135.5, 128.6, 128.5, 128.4, 110.8, 102.7, 101.7, 100.4, 66.9; IR (KBr): 3152, 2360, 1729, 1396, 1209, 990, 696 cm^{-1}. HRMS (ESI): m/z $[M+Na]^+$ calcd for $[C_{13}H_{11}N_3O_2Na]^+$ 264.0749, found 264.0739. UV/Vis (CH_2Cl_2): λ_{max} (log ε) = 344 (3.32), 281 (4.15) nm. FL (CH_2Cl_2): λ_{max} = 442 nm. Φ_F = 0.011 (reference to 9,10-DPA; excited at 370 nm).

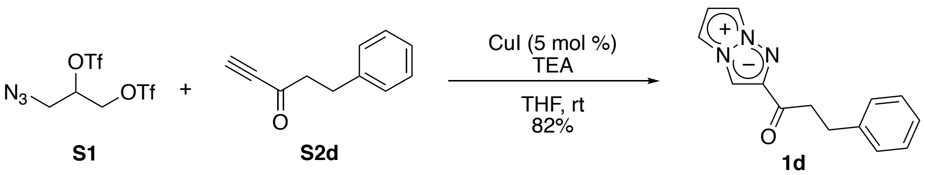

2-(3-phenylpropanoyl)pyrazolo[1,2-*a*][1,2,3]triazol-8-ium-1-ide (1d): brown solid. Mp 74 °C (dec.) (recrystallized from EtOAc). ^{1}H NMR ($CDCl_3$, 400 MHz): δ 7.61 (d, *J* = 1.2 Hz, 1H), 7.43 (br d, *J* = 3.0Hz, 1H), 7.31-7.26 (m, 4H), 7.21-7.17 (m, 2H), 6.75 (t, *J* = 3.0 Hz, 1H), 3.34 (t, *J* = 7.6 Hz, 2H), 3.08 (t, *J* = 7.6 Hz, 2H); ^{13}C NMR ($CDCl_3$, 100 MHz): δ 194.9, 146.4, 141.0, 128.4, 128.38, 126.0, 111.0, 102.2, 101.5, 98.0, 41.0, 29.7. IR (KBr): 3138, 3026, 2932, 1680, 1496, 1401, 1362, 1149, 710, 684 cm^{-1}. HRMS (ESI): m/z $[M+H]^+$ calcd for $[C_{14}H_{14}N_3O]^+$ 240.1137, found 240.1136. UV/Vis (CH_2Cl_2): λ_{max} (log ε) = 381 (3.18), 285 (4.02) nm. FL (CH_2Cl_2): λ_{max} = 515 nm. Φ_F = 0.22 (reference to 9,10-DPA; excited at 370 nm).

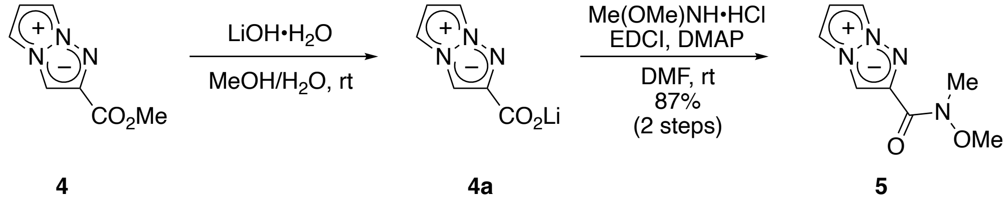

2-(methoxy(methyl)carbamoyl)pyrazolo[1,2-*a*][1,2,3]triazol-8-ium-1-ide (5): To a solution of methylester **4** (2.53 g, 15.3 mmol) in MeOH (57.4 mL) and H_2O (19.1 mL) was added LiOH·H_2O (674 mg, 16.08 mmol) at 0 °C. The mixture was stirred at room temperature for 24 h and concentrated under reduced pressure to give lithium salt **4a** as a brown amorphous material after azeotropic treatment with toluene. The residue was dissolved in DMF (76.6 mL) and the mixture was cooled to 0 °C. To the mixture were added EDCI (6.46 g, 33.7 mmol), DMAP (0.56 g, 4.59 mmol), and *N*,*O*-dimethylamino

hydrochloride (2.99 g, 30.6 mmol) at 0 °C, successively. The mixture was stirred at room temperature for 12 h, and the reaction was quenched with 5% aqueous citric acid. The mixture was extracted with EtOAc (x10). The combined organic layers were dried over anhydrous $MgSO_4$, filtered, and concentrated under reduced pressure. The residue was purified by silica gel flash column chromatography (EtOAc/hexane = 1/1, 1% Et_3N was contained) to give Weinreb amide **5** (2.83 g, 13.4 mmol, 87% in 2 steps) as a brown crystal. Mp 75 °C (dec.) (recrystallized from EtOAc). ^{1}H NMR ($CDCl_3$, 400 MHz): δ 7.67 (s, 1H), 7.44 (br d, J = 3.0 Hz, 1H), 7.17 (d, J = 3.0 Hz, 1H), 6.71 (t, J = 3.0 Hz, 1H), 3.81 (s, 3H), 3.45 (s, 3H). ^{13}C NMR ($CDCl_3$, 100 MHz): δ 161.1, 140.3, 110.3, 102.0, 101.0, 100.0, 61.5, 33.8. IR (KBr): 3147, 2934, 1638, 1417, 964 cm^{-1}. HRMS (ESI): m/z [M+Na]$^+$ calcd for $[C_8H_{10}N_4O_2Na]^+$ 217.0701, found 217.0692. UV/Vis (CH_2Cl_2): λ_{max} (log ε) = 331 (3.45), 282 (4.24) nm. FL (CH_2Cl_2): λ_{max} = 432 nm. Φ_F = 0.00079 (reference to 9,10-DPA; excited at 370 nm).

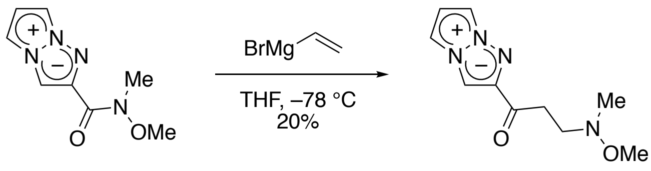

5 **S3a**

2-(3-(methoxy(methyl)amino)propanoyl)pyrazolo[1,2-a][1,2,3]triazol-8-ium-1-ide (S3a): To a solution of Weinreb amide **5** (46.5 mg, 0.237 mmol) in THF (395 μL) was added vinylmagnesium bromide (284 μL, 1.0 M solution in THF) at –78 °C. The mixture was stirred at –78 °C for 2 h, and the reaction was quenched with saturated aqueous NH_4Cl. The mixture was extracted with EtOAc (x3). The combined organic layers were washed with brine, dried over anhydrous $MgSO_4$, filtered, and concentrated under reduced pressure. The residue was purified by silica gel flash column chromatography (EtOAc/Hex = 3/7, 1% Et_3N was contained) to give **S3a** (10.6 mg, 0.047 mmol, 20%) as a yellow amorphous. ^{1}H NMR ($CDCl_3$, 400 MHz): δ 7.63 (d, J = 1.2 Hz, 1H), 7.45 (br d, J = 3.0 Hz, 0.8Hz, 1H), 7.20 (d, J = 3.0 Hz, 1H), 6.76 (t, J = 3.0 Hz, 1H) 3.49 (s, 3H), 3.26 (t, J = 6.8 Hz, 2H), 3.11 (t, J = 6.8 Hz, 2H), 2.62 (s, 3H); ^{13}C NMR ($CDCl_3$, 125 MHz,): δ 194.7, 146.6, 111.1, 102.3, 101.6, 98.1, 60.0, 55.3, 45.2, 37.4. IR (KBr): 3146, 2973, 2937, 1687, 1370, 1041 cm^{-1}. HRMS (ESI): m/z [M+H]$^+$ calcd for $[C_{10}H_{15}N_4O_2]^+$ 223.1195, found 223.1200.

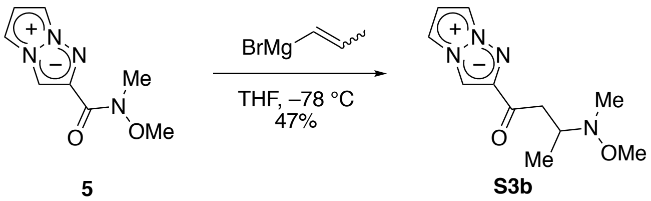

2-(3-(methoxy(methyl)amino)butanoyl)pyrazolo[1,2-a][1,2,3]triazol-8-ium-1-ide (**S3b**): To a solution of Weinreb amide **5** (10.0 mg, 0.051 mmol) in THF (102 μL) was added 1-Propenylmagnesium bromide solution (ca. 0.5 mol/L in THF solution, 102 μL, 0.051 mmol) at –78 °C. The mixture was stirred at –78 °C for 2 h, quenched with saturated aqueous NH_4Cl and extracted with EtOAc (x3). The combined organic layers were washed with brine, dried over anhydrous $MgSO_4$, filtered, and concentrated under reduced pressure. The residue was purified by PTLC (EtOAc/Hex = 1:1, 1% Et_3N was contained) to give **S3b** (5.7 mg, 0.024 mmol, 47%) as a yellow amorphous. ^{1}H NMR ($CDCl_3$, 500 MHz): δ 7.63 (d, *J* = 1.5 Hz, 1H), 7.45 (br d, *J* = 2.9 Hz, 1H), 7.19 (d, *J* = 2.9 Hz, 1H), 6.75 (t, *J* = 2.9 Hz, 1H) 3.58-3.51 (m, 1H), 3.44 (s, 3H), 3.38 (dd, *J* = 15.8 Hz, 6.0 Hz, 1H), 2.91 (dd, *J* = 15.8 Hz, 7.0 Hz, 2H), 2.59 (s, 3H), 1.15 (d, *J* = 6.7 Hz, 3H). ^{13}C NMR ($CDCl_3$, 125 MHz): δ 194.7, 146.9, 111.0, 102.3, 101.5, 98.2, 60.0, 58.7, 43.1, 40.7 (one peak missing in $CDCl_3$). IR (KBr): 3151, 2927, 2853, 2806, 1687, 1380, 1046 cm^{-1}. HRMS (ESI): m/z [M+Na]$^+$ calcd for $[C_{11}H_{16}N_4O_2Na]^+$ 259.1171, found 259.1164.

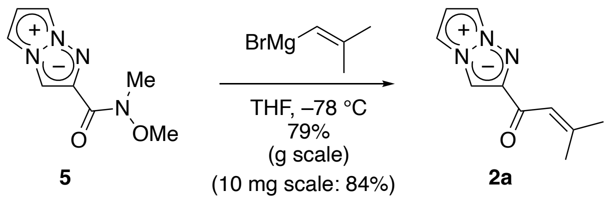

TAP-VK1 (2-(3-methylbut-2-enoyl)pyrazolo[1,2-*a*][1,2,3]triazol-8-ium-1-ide) (2a): To a solution of Weinreb amide **5** (3.3 g, 16.8 mmol) in THF (33.6 mL) was added isobutenylmagnesium bromide (67.3 mL, 0.5 M solution in THF) at –78 °C. The mixture was stirred at –78 °C for 2 h, and the reaction was quenched with saturated aqueous $NaHCO_3$. The mixture was extracted with EtOAc (x3). The combined organic layers were washed with brine, dried over anhydrous $MgSO_4$, filtered, and concentrated under reduced pressure. The residue was purified by silica gel flash column chromatography (EtOAc/Hex = 1/4, 1% Et_3N was contained) to give TAP-VK1 (**2a**) (2.51 g, 13.3 mmol, 79%) as a yellow solid. Mp 96 °C (dec.) (recrystallized from EtOAc); ^{1}H NMR ($CDCl_3$, 400 MHz): δ 7.64 (d, *J* = 1.2 Hz, 1H), 7.44 (d, *J* = 3.0 Hz, 1H), 7.18 (d, *J* = 3.0 Hz, 1H), 6.97-6.96 (m, 1H), 6.74 (t, *J* = 3.0 Hz, 1H), 2.31 (d, *J* = 0.9 Hz, 3H), 2.04 (d, *J* = 1.2 Hz). ^{13}C NMR ($CDCl_3$, 100

MHz): δ 184.5, 159.0, 148.3, 120.7, 110.8, 102.1, 101.3, 98.4, 28.1, 21.4. IR (KBr): 3125, 1663, 1612, 1551, 1375, 966, 853, 680 cm^{-1}. HRMS (ESI): m/z $[M+Na]^+$ calcd for $[C_{10}H_{11}N_3ONa]^+$ 212.0800, found 212.0799. UV/Vis (CH_2Cl_2): λ_{max} (log ε) = 409 (3.17), 279 (4.47) nm. FL (CH_2Cl_2): λ_{max} = 574 nm. Φ_F = 0.023 (reference to Rhodamine B; excited at 370 nm).

* 10 mg scale: 84%

The reaction of ethanthiol to TAP-VK1 as a typical procedure of the reaction with thiols

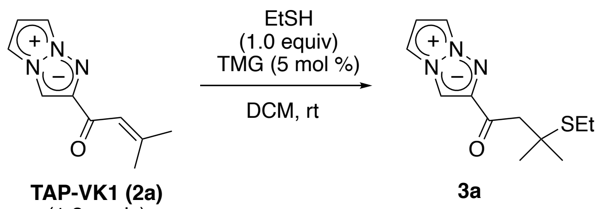

2-(3-(ethylthio)-3-methylbutanoyl)pyrazolo[1,2-*a*][1,2,3]triazol-8-ium-1-ide (3a): To a solution of TAP-VK1 (**2a**) (20.0 mg, 0.11 mmol) in DCM (0.53 mL) were added TMG (0.66 μL, 5.29 μmol) and ethanthiol (6.52 μL, 0.088 mmol) at 0 °C, successively. The mixture was stirred at room temperature for 3 h, and the reaction was quenched with saturated NH_4Cl. The mixture was extracted with EtOAc (x3). The combined organic layers were washed with brine, dried over anhydrous $MgSO_4$, filtered, and concentrated under reduced pressure. The residue was purified by silica gel flash column chromatography (EtOAc/Hex = 1/4, 1% Et_3N was contained) to give thiol adduct **3a** (19.3 mg, 7.7 μmol, 87%) as a yellow amorphous. ^{1}H NMR ($CDCl_3$, 400 MHz): δ 7.62 (d, J = 1.1 Hz, 1H), 7.45 (br d, J = 2.2 Hz, 1H), 7.19 (d, J = 2.8 Hz, 1H), 6.77 (t, J = 2.8 Hz, 1H), 3.27 (s, 2H), 2.66 (q, J = 7.4 Hz, 2H), 1.49 (s, 6H), 1.21 (t, J = 7.4 Hz, 3H). ^{13}C NMR ($CDCl_3$, 100 MHz): δ 193.8, 147.6, 111.1, 102.2, 101.4, 98.1, 49.7, 44.1, 28.9, 22.3, 14.3. IR (KBr): 3150, 2967, 2927, 2360, 1681, 1550, 1364, 670 cm^{-1}. HRMS (ESI): m/z $[M+Na]^+$ calcd for $[C_{12}H_{17}N_3ONaS]^+$ 274.0990, found 274.0983. UV/Vis (CH_2Cl_2): λ_{max} (log ε) = 389 (3.20), 285 (4.11) nm. FL (CH_2Cl_2): λ_{max} = 521 nm. Φ_F = 0.19 (reference to 9,10-DPA; excited at 370 nm).

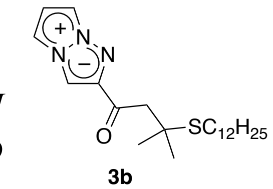

3b

2-(3-(dodecylthio)-3-methylbutanoyl)pyrazolo[1,2-*a*][1,2,3]triazol-8-ium-1-ide (3b): Yellow amorphous material; ^{1}H NMR ($CDCl_3$, 500 MHz): δ 7.61 (d, J = 1.3 Hz, 1H), 7.43 (br d, J = 2.9, 1H), 7.19 (d, J = 2.9 Hz, 1H), 6.75 (t, J = 2.9 Hz, 1H), 3.27 (s, 2H), 2.61 (t, J = 7.5 Hz, 2H), 1.55-1.49 (m, 2H), 1.49 (s, 6H), 1.40-1.15 (m, 18H), 0.87 (t, J = 7.1, 3H). ^{13}C NMR ($CDCl_3$, 125 MHz): δ 193.8, 147.6, 111.0, 102.1, 101.4, 98.1, 49.6, 44.0, 31.9, 29.61, 29.59, 29.55, 29.47, 29.43, 29.3, 29.25 (x2), 28.9, 28.3, 22.6, 14.1. IR (KBr): 3155, 2924, 2853, 1683, 1550, 1463, 1365, 668 cm^{-1}.

HRMS (ESI): m/z [M+Na]$^+$ calcd for [$C_{22}H_{37}N_3ONaS$]$^+$ 414.2555, found 414.2544. UV/Vis (CH_2Cl_2): λ_{max} (log ε) = 388 (3.24), 285 (4.21) nm. FL (CH_2Cl_2): λ_{max} = 519 nm. Φ_F = 0.14 (reference to 9,10-DPA; excited at 370 nm).

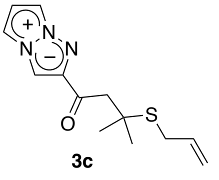

3c

2-(3-(allylthio)-3-methylbutanoyl)pyrazolo[1,2-*a*][1,2,3]triazol-8-ium-1-ide (3c): Yellow amorphous material. ^{1}H NMR ($CDCl_3$, 400 MHz): δ 7.61 (d, *J* = 1.3 Hz, 1H), 7.44 (br d, *J* = 2.5, 1H), 7.19 (d, *J* = 2.8 Hz, 1H), 6.76 (t, *J* = 2.8 Hz, 1H), δ 5.84 (ddt, *J* = 17.0, 10.1, 7.0 Hz, 1H), δ 5.18 (dd, *J* = 17.0, 1.3 Hz, 1H), 5.04 (dd, *J* = 10.1, 1.0 Hz, 1H), 3.33 (d, *J* = 7.0 Hz, 2H), 3.29 (s, 2H) 1.51 (s, 6H). ^{13}C NMR ($CDCl_3$, 100 MHz): δ 193.6, 147.5, 134.8, 117.0, 111.1, 102.2, 101.4, 98.1, 49.7, 44.8, 32.0, 28.9. IR (KBr): 3152, 2965, 2924, 2360, 1681, 1550, 1365, 671 cm^{-1}. HRMS (ESI): m/z [M+Na]$^+$ calcd for [$C_{13}H_{17}N_3ONaS$]$^+$ 286.0990, found 286.0980. UV/Vis (CH_2Cl_2): λ_{max} (log ε) = 388 (3.24), 285 (4.21) nm. FL (CH_2Cl_2): λ_{max} = 525 nm. Φ_F = 0.34 (reference to 9,10-DPA; excited at 370 nm).

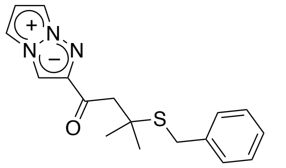

3d

2-(3-(benzylthio)-3-methylbutanoyl)pyrazolo[1,2-*a*][1,2,3]triazol-8-ium-1-ide (3d): Yellow solid. Mp 89 °C (dec.) (recrystallized from EtOAc). ^{1}H NMR ($CDCl_3$, 400 MHz): δ 7.60 (d, *J* = 1.2 Hz, 1H), 7.45 (br d, *J* = 2.7, 1H), 7.35-7.15 (m, 6H), 6.77 (t, *J* = 2.9 Hz, 1H), 3.89 (s, 2H), 3.32 (s, 2H), 1.53 (s, 6H). ^{13}C NMR ($CDCl_3$, 100 MHz): δ 193.6, 147.5, 138.0, 129.0, 128.4, 126.7, 111.1, 102.2, 101.5, 98.1, 49.5, 45.0, 33.4, 28.9. IR (KBr): 3465, 3124, 2362, 1670, 1364, 951, 695 cm^{-1}. HRMS (ESI): m/z [M+H]$^+$ calcd for [$C_{17}H_{20}N_3OS$]$^+$ 314.1327, found 314.1324. UV/Vis (CH_2Cl_2): λ_{max} (log ε) = 389 (3.14), 285 (4.09) nm. FL (CH_2Cl_2): λ_{max} = 517 nm. Φ_F = 0.16 (reference to 9,10-DPA; excited at 370 nm).

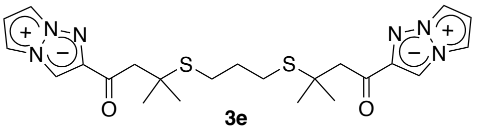

3e

2,2'-(3,3'-(propane-1,3-diylbis(sulfanediyl))bis(3-methylbutanoyl))bis(pyrazolo[1,2-*a*][1,2,3]triazol-8-ium-1-ide) (3e): Yellow amorphous material. ^{1}H NMR ($CDCl_3$, 400 MHz): δ 7.62 (s, 2H), 7.45 (br d, *J* = 0.8 Hz, 2H), 7.19 (d, *J* = 3.0 Hz, 2H), 6.75 (dt, *J* = 3.0, 0.7 Hz, 2H), 3.25 (s, 4H), 2.70 (t, *J* = 7.2 Hz, 4H), 1.77 (quint, *J* = 7.2 Hz, 2H), 1.47 (s, 12H). ^{13}C NMR ($CDCl_3$, 100 MHz): δ 193.7, 147.5, 111.1, 102.2, 101.5, 98.1, 49.6, 44.3, 29.4, 28.9, 27.6. IR (KBr): 3148, 2959, 2924, 2359, 1681, 1549, 1489, 1457, 1364, 370 cm^{-1}. HRMS (ESI): m/z [M+Na]$^+$ calcd for [$C_{23}H_{30}N_6O_2NaS_2$]$^+$ 509.1769, found 509.1769. UV/Vis (CH_2Cl_2): λ_{max} (log ε) = 390 (3.36), 284 (4.28) nm. FL (CH_2Cl_2): λ_{max} = 536 nm. Φ_F = 0.13 (reference to 9,10-DPA; excited at 370 nm).

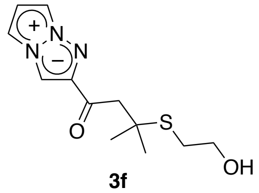

3f

2-(3-((2-hydroxyethyl)thio)-3-methylbutanoyl)pyrazolo[1,2-*a*][1,2,3]triazol-8-ium-1-ide (3f): Yellow amorphous material. ^{1}H NMR ($CDCl_3$, 400 MHz): δ 7.63 (d, *J* = 1.4 Hz, 1H), 7.46 (ddd, *J* = 3.0, 1.5, 0.7 Hz, 1H), 7.21 (dd, *J* = 3.0, 0.7 Hz, 1H), 6.77 (t, *J* = 3.0 Hz, 1H), 3.75 (t, *J* = 5.8 Hz, 1H), 3.27 (s, 2H), 2.87 (t, *J* = 5.8 Hz, 2H), 1.50 (s, 6H). ^{13}C NMR ($CDCl_3$, 100 MHz): δ 193.8, 147.4, 111.2, 102.3, 101.6, 98.2, 61.3, 49.6, 44.6, 32.2, 29.3. IR (KBr): 3369, 3148, 2962, 2360, 2341, 1677, 1550, 1365, 668 cm^{-1}. HRMS (ESI): m/z $[M+Na]^+$ calcd for $[C_{12}H_{17}N_3O_2NaS]^+$ 290.0939, found 290.0940. UV/Vis (CH_2Cl_2): λ_{max} (log ε) = 391 (3.08), 284 (4.01) nm. FL (CH_2Cl_2): λ_{max} = 532 nm. Φ_F = 0.18 (reference to 9,10-DPA; excited at 360 nm).

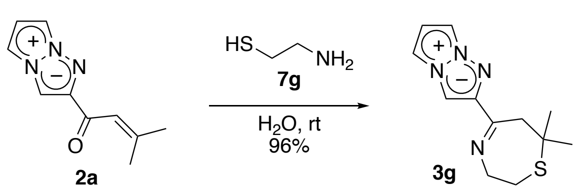

2-(7,7-dimethyl-2,3,6,7-tetrahydro-1,4-thiazepin-5-yl)pyrazolo[1,2-*a*][1,2,3]triazol-8-ium-1-ide (3g): To a solution of TAP-VK1 (**2a**) (20.3 mg, 0.11 mmol) in H_2O (447 μL) was added aminoethanethiol **7g** (6.9 mg, 0.089 mmol) at room temperature. The mixture was stirred at room temperature for 3 h and concentrated under reduced pressure. The residue was purified by silica gel flash column chromatography (EtOAc/Hex = 1/1, 1% Et_3N was contained) to give thiol adduct **3g** (22.9 mg, 0.086 mmol, 96%) as a yellow crystal. Mp 110 °C (dec.). ^{1}H NMR ($CDCl_3$, 400 MHz): δ 7.47 (d, *J* = 1.2 Hz, 1H), 7.39 (br d, *J* = 2.8 Hz, 1H), 7.11 (dd, *J* = 2.9, 0.5 Hz, 1H), 6.66 (t, *J* = 2.9 Hz, 1H), 4.28-4.21 (m, 2H), 3.45 (s, 2H), 2.83-2.72 (m, 2H), 1.42 (s, 6H). ^{13}C NMR (125 MHz, D_2O): δ 165.3, 149.6, 109.6, 101.9, 100.5, 95.4, 55.3, 47.0, 38.4, 29.6, 25.0. IR (KBr): 3149, 2960, 2919, 2854, 1636, 1365 cm^{-1}. HRMS (ESI): m/z $[M+H]^+$ calcd for $[C_{12}H_{17}N_4S]^+$ 249.1174, found 249.1172. UV/Vis (CH_2Cl_2): λ_{max} (log ε) = 360 (3.36), 288 (4.19) nm. FL (CH_2Cl_2): λ_{max} = 471 nm. Φ_F = 0.23 (reference to 10-DPA; excited at 360 nm).

Synthesis of thiol 7h

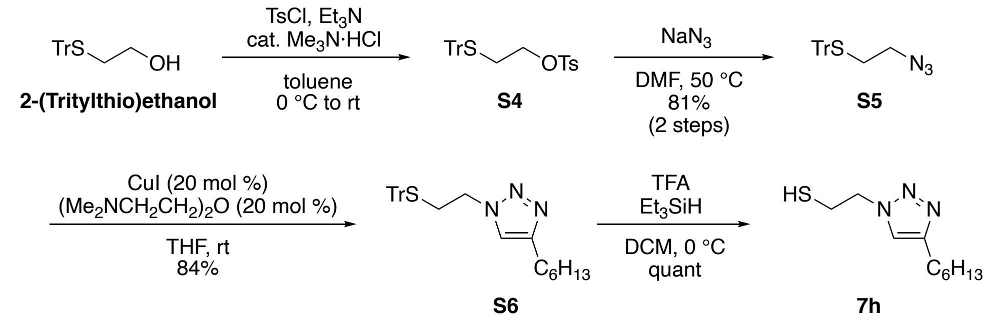

(2-azidoethyl)(trityl)sulfane (S5): To a solution of 2-(tritylthio)ethanol (533 mg, 1.66 mmol) in toluene (8.3 mL) were added Et_3N (694 µL, 4.98 mmol), TsCl (652 mg, 3.32 mmol), and $Me_3N{\cdot}HCl$ (15.9 mg, 0.17 mmol) at 0 °C, successively. The mixture was stirred at room temperature for 5 h, and the reaction was quenched with H_2O. The mixture was extracted with EtOAc (x3). The combined organic layers were dried over $MgSO_4$, filtered, and concentrated under reduced pressure. The crude residue of **S4** was used for the next reaction without purification. To a solution of the residue in DMF (8.3 mL) was added NaN_3 (162 mg, 2.49 mmol). The reaction mixture was stirred at 50 °C for 2 h, and the reaction was quenched with H_2O. The mixture was extracted with Et_2O (x3). The combined organic layers were dried over $MgSO_4$, filtered, and concentrated under reduced pressure. The residue was purified by silica gel flash column chromatography (EtOAc/Hex = 1/50) to give azide **S5** (462 mg, 81%) as a pale yellow amorphous. ^{1}H NMR ($CDCl_3$, 500 MHz): δ 7.47-7.40 (m, 6H), 7.32-7.25 (m, 6H), 7.25-7.19 (m, 3H), 2.92 (t, J = 7.3 Hz, 2H), 2.43 (t, J = 7.6 Hz, 2H). ^{13}C NMR (125 MHz, $CDCl_3$): δ 144.5, 129.5, 128.0, 126.8, 67.1, 50.2, 31.2. IR (KBr): 2100, 1488, 1444, 742, 700 cm^{-1}. HRMS (ESI): m/z $[M+H]^+$ calcd for $[C_{21}H_{20}N_3S]^+$ 346.1378, found 346.1370.

4-hexyl-1-(2-(tritylthio)ethyl)-1*H*-1,2,3-triazole (S6): To a solution of azide **S5** (72.5 mg, 0.21 mmol) in THF (1.3 mL) were added 1-octyne (39.0 µL, 0.25 mmol), bis[2-(*N*,*N*-dimethylaminoethyl)]ether (8.3 µL, 0.042 mmol), and CuI (8.0 mg, 0.042 mmol) at room temperature, successively. The mixture was stirred at room temperature for 3 h and concentrated under reduced pressure. The residue was purified by silica gel flash column chromatography (EtOAc/Hex = 1/8) to give **S6** (98.4 mg, 86%) as a colorless oil. ^{1}H NMR ($CDCl_3$, 400 MHz): δ 7.44-7.38 (m, 6H), 7.34-7.27 (m, 6H), 7.26-7.20 (m, 3H), 7.00 (s, 1H), 3.85 (t, J = 7.3 Hz, 2H), 2.74 (t, J = 7.3 Hz, 2H), 2.65 (t, J = 7.5 Hz, 2H), 1.64-1.50 (m, 2H), 1.36-1.25 (m, 6H), 0.87 (t, J = 7.0 Hz, 3H). ^{13}C NMR ($CDCl_3$, 125 MHz): δ 148.2, 144.3, 129.5, 128.1, 127.0, 120.9, 67.3, 48.9, 32.1, 31.6, 29.4, 28.9, 25.6, 22.6, 14.1. IR (KBr): 2925, 2854, 743, 700. HRMS (ESI): m/z $[M+Na]^+$ calcd for $[C_{29}H_{33}N_3SNa]^+$ 478.2293, found 478.2287.

2-(4-hexyl-1*H*-1,2,3-triazol-1-yl)ethane-1-thiol (7h): To a stirred solution of **S6** (10.0 mg, 0.022 mmol) in CH_2Cl_2 (1.1 mL) were added Et_3SiH (35.6 µL, 0.22 mmol) and TFA (629 µL, 8.47 mmol)

at 0 °C, successively. The mixture was stirred at 0 °C for 15 min, diluted with EtOAc, and washed with H_2O. The organic layer was dried over $MgSO_4$, filtered, and concentrated under reduced pressure. The residue was purified by silica gel flash column chromatography (EtOAc/Hex = 1:3) to give **7h** (5.0 mg, quant) as a colorless amorphous. ^{1}H NMR ($CDCl_3$, 500 MHz): δ 7.34 (s, 1H), 4.49 (t, J = 6.6 Hz, 2H), 3.01 (dt, J = 8.9, 6.6 Hz, 2H), 2.70 (t, J = 7.6 Hz, 2H), 1.66 (quint, J = 6.6 Hz, 2H), 1.41 (t, J = 8.9 Hz, 1H (SH)), 1.40-1.20 (m, 6H), 0.87 (t, J = 7.0 Hz, 3H). ^{13}C NMR (125 MHz, $CDCl_3$): δ 148.4, 121.3, 52.9, 31.6, 29.4, 28.9, 25.7, 24.9, 22.6, 14.1. IR (KBr): 2927, 2856, 1457, 1216, 1048 cm^{-1}. HRMS (ESI): m/z [M+Na]$^+$ calcd for $[C_{10}H_{19}N_3SNa]^+$ 236.1197, found 236.1195.

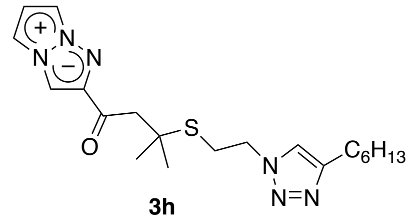

2-(3-((2-(4-hexyl-1*H*-1,2,3-triazol-1-yl)ethyl)thio)-3-methylbutanoyl)pyrazolo[1,2-*a*][1,2,3]triazol-8-ium-1-ide (3h): Yellow solid. Mp 65 °C (dec.) (recrystallized from EtOAc). ^{1}H NMR ($CDCl_3$, 500 MHz): δ 7.62 (d, J = 1.3 Hz, 1H), 7.45 (br-d, J = 2.8 Hz, 1H), 7.33 (s, 1H), 7.21 (d, J = 3.0 Hz, 1H), 6.77 (t, J = 3.0 Hz, 1H), 4.45 (t, J = 7.2 Hz, 2H), 3.25 (s, 2H), 3.13 (t, J = 7.2 Hz, 2H), 2.69 (t, J = 7.7 Hz, 2H), 1.65 (quint, J = 7.7 Hz, 2H), 1.48 (s, 6H), 1.45–1.21 (m, 6H), 0.88 (t, J = 7.0 Hz, 3H). ^{13}C NMR ($CDCl_3$, 125 MHz): δ 193.3, 148.3, 147.4, 121.1, 111.3, 102.4, 101.7, 98.1, 50.0, 49.4, 45.1, 31.6, 29.4, 29.0, 28.9, 28.8, 25.7, 22.6, 14.1. IR (KBr): 3145, 2956, 2927, 2856, 1681, 1550, 1458, 1366 cm^{-1}. HRMS (ESI): m/z [M+H]$^+$ calcd for $[C_{20}H_{31}N_6OS]^+$ 403.2280, found 403.2276. UV/Vis (CH_2Cl_2): λ_{max} (log ε) = 392 (3.25), 284 (4.18) nm. FL (CH_2Cl_2): λ_{max} = 520 nm. Φ_F = 0.30 (reference to 9,10-DPA; excited at 370 nm).

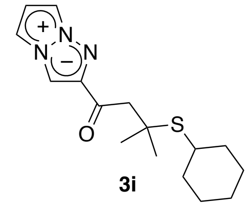

2-(3-(cyclohexylthio)-3-methylbutanoyl)pyrazolo[1,2-*a*][1,2,3]triazol-8-ium-1-ide (3i): Yellow amorphous material. ^{1}H NMR ($CDCl_3$, 500 MHz): δ 7.61 (d, J = 1.3 Hz, 1H), 7.44 (br d, J = 3.0, 1H), 7.19 (d, J = 3.0 Hz, 1H), 6.75 (t, J = 3.0 Hz, 1H), 3.30 (s, 2H), 2.79 (m, 1H), 1.98-1.85 (m, 2H), 1.76-1.62 (m, 2H), 1.65-1.49 (m, 1H), 1.50 (s, 6H), 1.42-1.15 (m, 5H). ^{13}C NMR ($CDCl_3$, 100 MHz): δ 193.8, 147.6, 111.1, 102.2, 101.4, 98.1, 50.3, 45.4, 41.3, 36.1, 29.5, 26.4, 25.5. IR (KBr): 3152, 2927, 2851, 2360, 1682, 1550, 1446, 1364, 668 cm^{-1}. HRMS (ESI): m/z [M+Na]$^+$ calcd for $[C_{16}H_{23}N_3ONaS]^+$ 328.1460, found 328.1453. UV/Vis (CH_2Cl_2): λ_{max} (log ε) = 389 (3.17), 285 (4.14) nm. FL (CH_2Cl_2): λ_{max} = 521 nm. Φ_F = 0.27 (reference to 9,10-DPA; excited at 370 nm).

(*R*)-2-(3-((2-((*tert*-butoxycarbonyl)amino)-3-methoxy-3-oxopropyl)thio)-3-methylbutanoyl)pyrazolo[1,2-*a*][1,2,3]triazol-8-ium-1-ide (3j): Yellow amorphous material. ^{1}H NMR ($CDCl_3$, 400 MHz): δ 7.62 (s, 1H), 7.45 (d, *J* = 2.7 Hz, 1H), 7.19 (d, *J* = 2.7 Hz, 1H), 6.76 (t, *J* = 2.7 Hz, 1H), 5.35 (br d, *J* = 7.6 Hz, 1H), 4.5 (m, 1H), 3.73 (s, 3H), 3.24 (s, 2H), 3.09 (d, *J* = 5.0 Hz, 2H), 1.48 (s, 3H), 1.47 (s, 3H), 1.42 (s, 9H). ^{13}C NMR ($CDCl_3$, 125 MHz): δ 193.4, 171.4, 147.4, 111.2, 102.4, 101.6, 98.2, 80.0, 53.1, 52.5, 49.4, 44.7, 30.8, 28.8, 28.3. IR (KBr): 3316, 3154, 2976, 2932, 1745, 1680, 1517, 1366, 1168 cm^{-1}. HRMS (ESI): m/z $[M+H]^+$ calcd for $[C_{19}H_{29}N_4O_5S]^+$ 425.1859, found 425.1855. UV/Vis (CH_2Cl_2): λ_{max} (log ε) = 390 (3.34), 284 (4.23) nm. FL (CH_2Cl_2): λ_{max} = 521 nm. Φ_F = 0.12 (reference to 9,10-DPA; excited at 370 nm). $[\alpha]^{28}_D$ +9.6° (*c* 0.81, $CHCl_3$).

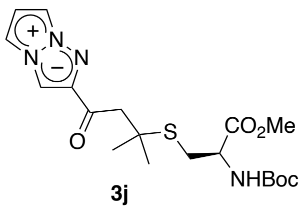

3j

2-((6*S*,9*R*)-9-(methoxycarbonyl)-2,2,6,12,12-pentamethyl-4,7-dioxo-3-oxa-11-thia-5,8-diazatetradecan-14-oyl)pyrazolo[1,2-*a*][1,2,3]triazol-8-ium-1-ide (3k): Yellow amorphous material. ^{1}H NMR ($CDCl_3$, 400 MHz): δ 7.64 (d, *J* = 1.3 Hz, 1H), 7.46 (br d, *J* = 2.1 Hz, 1H), 7.21 (d, *J* = 3.0 Hz, 1H), 7.14 (br d, *J* = 4.9 Hz, 1H), 6.77 (t, *J* = 3.0 Hz, 1H), 5.36 (br s, 1H), 4.81 (dt, *J* = 7.5, 4.9 Hz, 1H), 4.26 (m, 1H), 3.74 (s, 3H), 3.23 (s, 2H), 3.17 (dd, *J* = 12.9, 5.2 Hz, 1H), 3.07 (dd, *J* = 12.9, 4.7 Hz, 1H), 1.48 (s, 3H), 1.45 (s, 3H), 1.45 (s, 9H), 1.38 (d, *J* = 7.2 Hz, 3H). ^{13}C NMR ($CDCl_3$, 125 MHz): δ 193.5, 172.6, 170.7, 155.4, 147.3, 111.2, 102.4, 101.7, 98.3, 80.0, 52.6, 51.8, 50.0, 49.3, 44.8, 30.3, 28.9, 28.7, 28.3, 26.7, 18.4. IR (KBr): 3316, 3153, 2975, 2931, 2360, 1681, 1507, 1366, 1168. HRMS (ESI): m/z $[M+H]^+$ calcd for $[C_{22}H_{34}N_5O_6S]^+$ 496.2230, found 496.2227. UV/Vis (CH_2Cl_2): λ_{max} (log ε) = 393 (3.26), 284 (4.17) nm. FL (CH_2Cl_2): λ_{max} = 540 nm. Φ_F = 0.17 (reference to 9,10-DPA; excited at 370 nm). $[\alpha]^{28}_D$ +2.7° (*c* 0.11, $CHCl_3$).

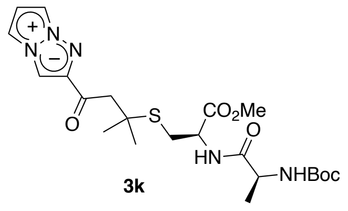

3k

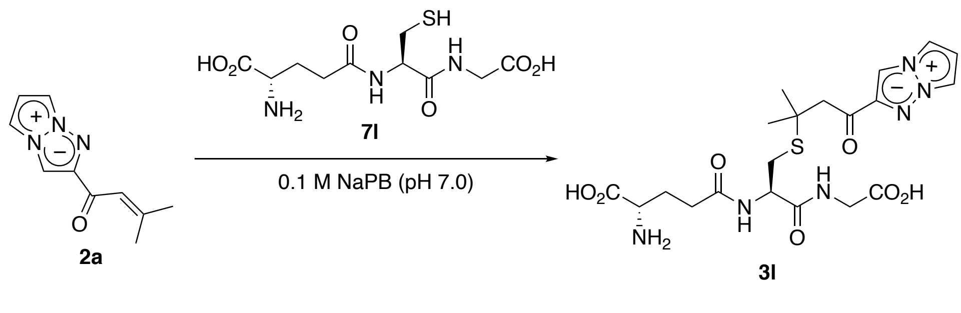

2-(3-(((*R*)-2-((*S*)-4-amino-4-carboxybutanamido)-3-((carboxymethyl)amino)-3-oxopropyl)thio)-3-methylbutanoyl)pyrazolo[1,2-*a*][1,2,3]triazol-8-ium-1-ide (3l): To a solution of glutathione (**7l**) (10.0 mg, 0.033 mmol) in phosphate buffer (pH 7.0, 15.9 mL, 2.04 mM) was added a solution of TAP-VK1 (**2a**) (12.3 mg, 0.065 mmol) in DMF (651 μL) at 27 °C. The mixture was incubated at the same temperature for 23 h and washed with CH_2Cl_2. The aqueous layer was concentrated under reduced pressure. The residue was purified by reverse-phase column chromatography (Wakogel 50C18, MeCN/H_2O = 1/99) to give **3l** (7.6 mg, 0.015 mmol, 47%) as a yellow amorphous. ^{1}H NMR (D_2O, 500 MHz): δ 8.02 (d, *J* = 1.1 Hz, 1H), 7.58 (d, *J* = 2.5 Hz, 1H), 7.48 (d, *J* = 2.5 Hz, 1H), 6.84 (t, *J* = 3.1 Hz, 1H), 4.44 (dd, *J* = 9.0, 5.0 Hz, 1H), 3.69 (dd, *J* = 17.3, 1.3 Hz, 1H), 3.67 (t, *J* = 6.4 Hz, 1H), 3.62 (dd, *J* = 17.3, 1.8 Hz, 1H), 3.20-3.05 (m, 3H), 2.90 (dd, *J* = 13.0, 8.8 Hz, 1H), 2.29 (t, *J* = 7.3 Hz, 2H), 2.02 (q, *J* = 7.0 Hz, 2H), 1.40 (s, 6H). ^{13}C NMR (D_2O, 125 MHz): δ 195.4, 176.1, 174.6, 173.9, 171.6, 145.4, 111.5, 54.0, 53.3, 49.3, 44.8, 43.2, 31.3, 29.6, 28.6, 28.5, 26.1. *The peaks at C3, C4, C5, and C6 of triazapentalene skeleton could not be detected because of their low measurement sensitivity by deuterium exchange. IR (KBr): 3271, 3148, 2966, 2360, 2341, 1651, 1605, 1549, 1392 cm^{-1}. HRMS (ESI): m/z [M+Na]$^+$ calcd for [$C_{20}H_{28}N_6O_7NaS$]$^+$ 519.1638, found 519.1630. UV/Vis (H_2O): λ_{max} (log ε) = 396 (3.44), 255 (4.30) nm. FL (H_2O): λ_{max} = 493 nm. Φ_F = 0.0004 (reference to 10-DPA; excited at 370 nm). $[\alpha]^{29}_D$ -14.5° (*c* 0.31, H_2O).

Synthesis of octapeptide 7n and R8-peptide 7o.

General Method and Procedure.

Fmoc-protected amino acids were purchased from Peptide Institute. Inc. or Novabiochem®. NovaSyn TGR resin and Rink Amide resin were purchased from NovabioChem®. Diisopropylcarbodiimide (DIC) and 1-hydroxybenzotriazole monohydrate (HOBt·H_2O) were purchased from Watanabe Chemical Industries. Ltd.. For HPLC purification and analysis, a Cosmosil 5C18-AR-II preparative column (Nacalai Tesque, 20 × 250 mm, flow rate 10 mL/min) and a Cosmosil 5C18-AR-II analytical column (Nacalai Tesque, 4.6 × 250 mm, flow rate 1.0 mL/min) were employed and eluting products were detected by UV at 220 nm. The peptides **7m** and **7n** were prepared by standard Fmoc solid phase peptide synthesis as follows.

Octapeptide (Ac-RSHKDYCA-NH$_2$) (7m): NovaSyn TGR resin (400 mg, loading 0.25 mmol/g; 0.10 mmol) was swollen with CH_2Cl_2 in a 20 mL disposable fritted polypropylene column (GL Sciences Inc. Japan) for 10 minutes and the solvent was drained. A mixture of Fmoc-protected amino acid (0.40 mmol), HOBt·H_2O (0.4 M in DMF, 1.00 mL, 0.40 mmol) and DIC (62.3 μL, 0.40 mmol) was vortexed in a separate container for 5 min and added to the swollen resin. The reaction mixture was shaken on a rotary shaker at room temperature for 2 h. The excess reagents were drained and the resin was washed with DMF five times. Reaction completion was monitored by Kaiser test. The Fmoc group was removed using a 20% solution of piperidine in DMF for 10 minutes. The resin was washed with DMF (x5). After the completion of the construction of whole peptide sequence, the resin was swollen with DMF. Then acetic anhydride (378 μL, 4.0 mmol) and pyridine (322 μL, 4.0 mmol) were added to cap the N-terminal amino group with Ac group. The completion of the capping was determined by Kaiser test. The resulting resin was washed with CH_2Cl_2 and dried under vacuum to produce the resin-bound crude peptide (550 mg). To the crude resin-bound peptide (158 mg) were successively added Et_3SiH (198 μL), H_2O (198 μL), and TFA (7.5 mL) at room temperature, and the mixture was stirred at the same temperature for 2 h. Then the mixture was passed through a filter to remove the insoluble resin and the filtrate was poured into cold Et_2O (200 mL) at 0 ºC. The resulting suspension was centrifuged at 4000 rpm for 5 min. The supernatant was decanted and the peptide pellet was added cold Et_2O, vortexed, centrifuged and the supernatant was decanted. This process was repeated five times. After that the residue was dissolved in H_2O/CH_3CN (8:2, containing 0.1 % TFA) and purified by reversed phase preparative HPLC (Solvent A: [H_2O, 0.1% TFA], Solvent B: [MeCN, 0.1% TFA]. Gradient: 5% [B] to 18% [B] from 0 to 30 min. Monitored by UV 220 nm, t_R = 21.7 min). The collected eluent was lyophilized to yield the pure peptide **7m** as a white powder (6.40 mg, 22% determined from the amount of the resin-bound peptide processed for cleavage).

Octapeptide (Ac-RSHKDYCA-NH$_2$) (7m): analytical HPLC properties (Solvent A: [H_2O, 0.1% TFA], Solvent B: [MeCN, 0.1% TFA]. Gradient: 5% [B] to 80% [B] from 0 to 30 min. Monitored by UV 220 nm, t_R = 7.90 min). MS (ESI$^+$): *m/z* calcd for $C_{42}H_{66}N_{15}O_{13}S$ ($[M + 2H]^{2+}$) 510.7, found 510.8.

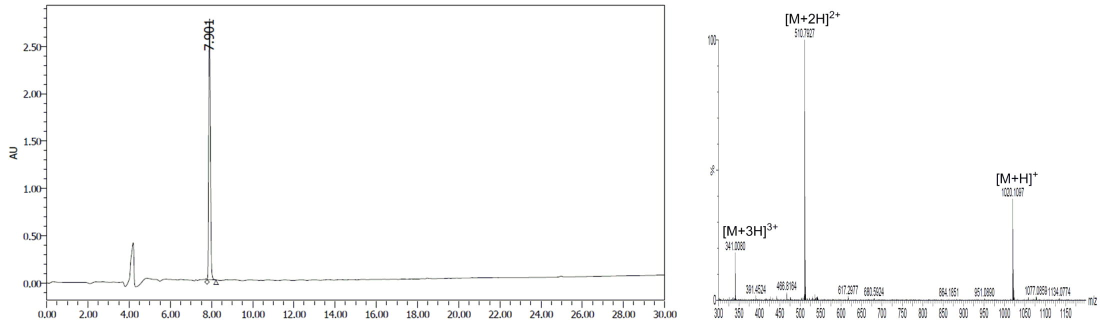

Supplementary Figure 10 | HPLC chart and MS spectrum of pure 7m.

R8-peptide (Ac-RSHKDYCA-NH_2) (7n): Rink Amide AM resin (323 mg, loading 0.62 mmol/g; 0.2 mmol) was swollen with CH_2Cl_2 in a 20 mL disposable fritted polypropylene column (GL Sciences Inc. Japan) for 10 minutes and the solvent was drained. A mixture of Fmoc-protected amino acid (0.60 mmol), HOBt·H_2O (0.6 M in DMF, 1.00 mL, 0.60 mmol) and DIC (93.5 μL, 0.60 mmol) was vortexed in a separate container for 5 min and added to the swollen resin. The reaction mixture was shaken on a rotary shaker at room temperature for 2 h. The excess reagents were drained and the resin was washed with DMF five times. Reaction completion was monitored by Kaiser test. The Fmoc group was removed using a 20% solution of piperidine in DMF for 10 minutes. The resin was washed with DMF (x5). The weight of the resin after installing eight arginines was 1.07 g and 201 mg of the resin was used for further elongation. After the completion of whole peptide sequence, the resin was swollen with DMF. Then acetic anhydride (189 μL, 2.0 mmol) and pyridine (161 μL, 2.0 mmol) were added to cap the N-terminal amino group with Ac group. The completion of the capping was determined by Kaiser test. The resulting resin was washed with CH_2Cl_2 and dried under vacuum to produce the resin-bound crude peptide (228 mg). To the crude resin-bound peptide (106 mg) were added *m*-cresol (250 μL), thioanisole (250 μL), ethanedithiol (125 μL), Et_3SiH (125 μL), H_2O (250 μL) and TFA (4.0 mL) successively at room temperature and stirred at the same temperature for 2 h. Then the mixture was passed through a filter to remove the insoluble resin and the filtrate was poured into cold Et_2O (200 mL) at 0 ºC. The resulting suspension was centrifuged at 4000 rpm for 5 min. The supernatant was decanted and the peptide pellet was added cold Et_2O, vortexed, centrifuged and the supernatant was decanted. This process was repeated five times. After that the residue was dissolved in H_2O/MeCN (8:2, containing 0.1 % TFA) and purified by reversed phase preparative HPLC (Solvent A: [H_2O, 0.1% TFA], Solvent B: [MeCN, 0.1% TFA]. Gradient: 11% [B] to 21% [B] from 0 to 30 min. Monitored by UV 220 nm, t_R = 20.3 min). The collected eluent was lyophilized to yield the pure peptide **7n** as a white powder (5.46 mg, 19% determined from the amount of the resin-bound peptide processed for cleavage).

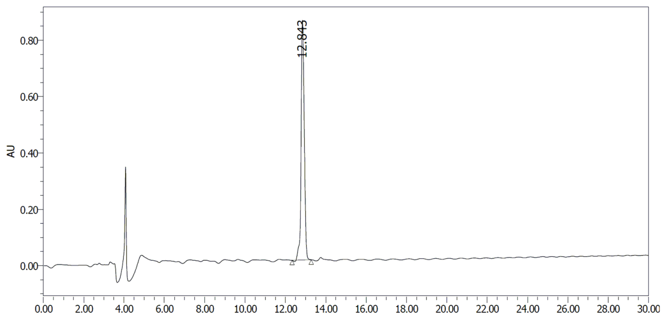

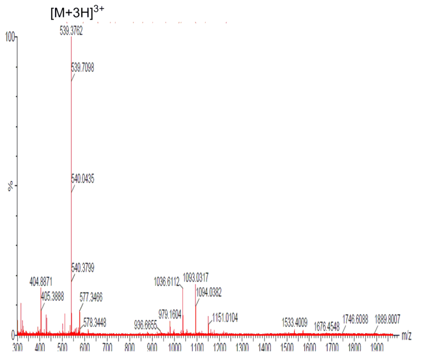

R8-peptide (Ac-RSHKDYCA-NH_2) (7n): analytical HPLC properties (Solvent A: [H_2O, 0.1% TFA], Solvent B: [MeCN, 0.1% TFA]. Gradient: 5% [B] to 50% [B] from 0 to 30 min. Monitored by UV 220 nm, t_R = 12.8 min). MS (ESI$^+$): *m/z* calcd for $C_{64}H_{121}N_{36}O_{12}S$ ($[M + 3H]^{3+}$) 539.3, found 539.4.

Supplementary Figure 11 | HPLC chart and MS spectrum of pure 7n.

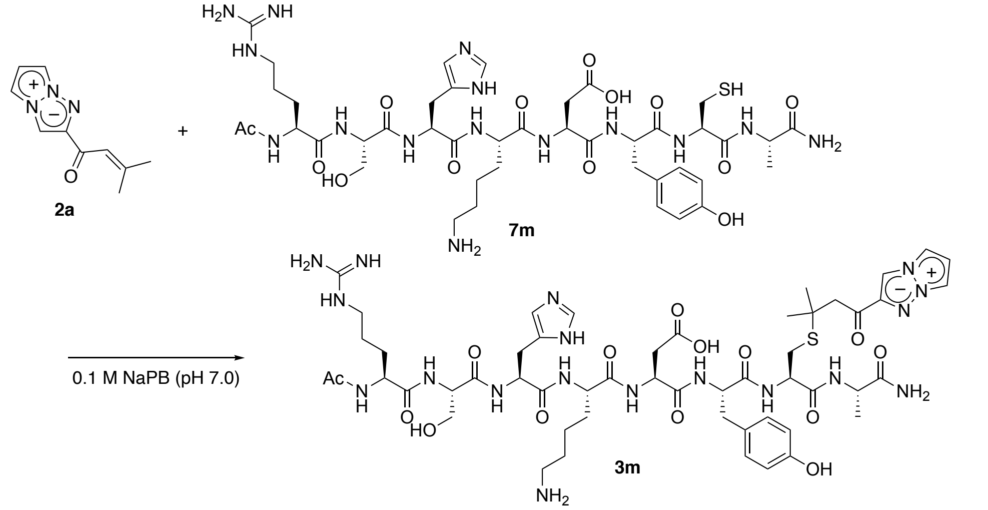

Compound 3m: To a solution of octapeptide **7m** (3.0 mg, 2.9 μmol) in 0.1 M phosphate buffer (pH 7.0, 2.9 mL) was added TAP-VK1 (**2a**) (59 μL, 2.9 μmol, 50 mM solution in DMF,) at 27 °C. The reaction was incubated at the same temperature for 6 h. The progress of the reaction was monitored by reverse-phase analytical HPLC (COSMOSIL 5C18-AR-Ⅱ, 4.6ID x 250 mm, Solvent A: [10 mM NH_4OAc buffer], Solvent B: [MeCN]. Gradient: 5% [B] to 50% [B] from 0 to 30 min. Monitored by UV 220 nm, t_R = 17.8 min). After completion of the reaction, the mixture was directly purified by reverse-phase preparative HPLC (Solvent A: [10 mM NH_4OAc buffer], Solvent B: [MeCN]. Gradient: 8% [B] to 28% [B] from 0 to 30 min. Monitored by UV 220 nm). The collected eluent was lyophilized to give **3m** (0.92 mg, 0.76 μmol, 26%) as a yellow powder. HRMS (ESI) m/z $[M+H]^+$ calcd for $[C_{52}H_{77}N_{18}O_{14}S]^+$ 1209.5587, found 1209.5579; UV/Vis (H_2O): λ_{max} (log ε) = 401 (3.20), 256 (4.29) nm. FL (H_2O): λ_{max} = 508 nm; Φ_F = 0 (reference to 9,10-DPA; excited at 360nm).

Analytical condition: Reverse-Phase HPLC (COSMOSIL 5C18-AR-Ⅱ, 4.6ID x 250 mm, Solvent A: [10 mM NH_4OAc buffer], Solvent B: [MeCN]. Gradient: 5% [B] to 50% [B] from 0 to 30 min. Monitored by UV 220 nm, t_R = 17.8 min)

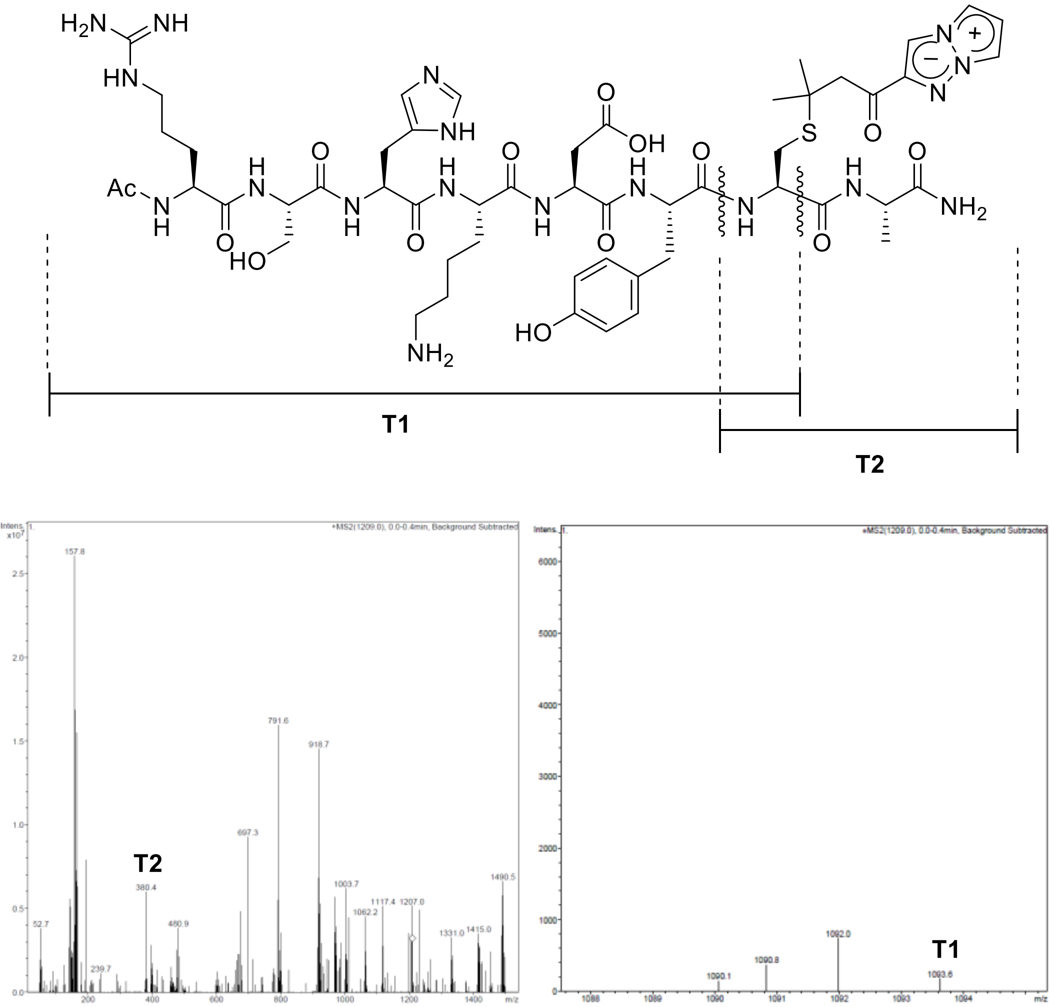

Supplementary Figure 12 | MS/MS analysis of 3m.

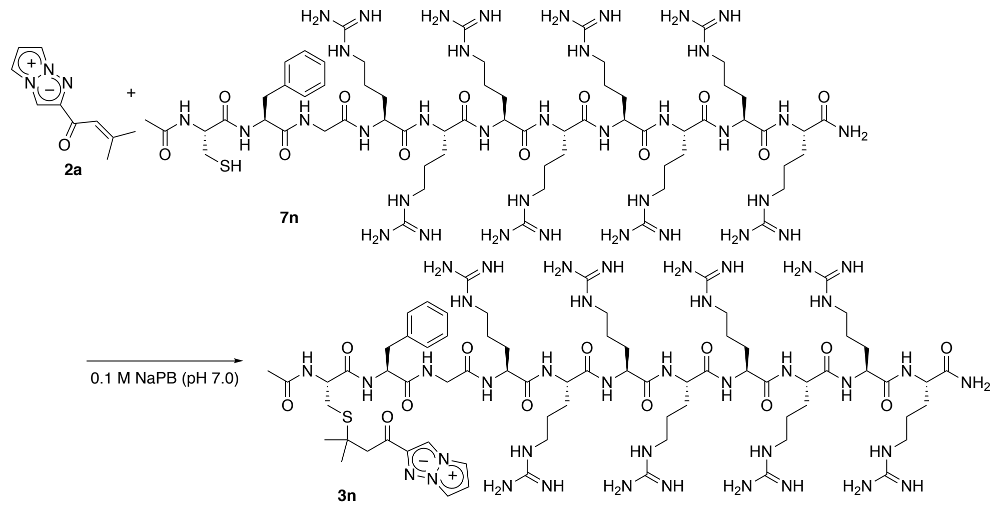

Compound 3n: To a solution of R8 peptide **7n** (pH 7.0, 1.0 mM in phosphate buffer, 98 µL) was added TAP-VK1 (**2a**) (100 mM in DMF, 2.0 µL) at 25 °C. The reaction was incubated at the same temperature for 3 h. The progress of the reaction was monitored by reverse-phase analytical HPLC (Inertsil ODS-P 5 µm, 4.6ID x 250 mm, Solvent A: [H_2O, 0.1% TFA], Solvent B: [MeCN]. Gradient: 5% [B] to 50% [B] from 0 to 30 min. Monitored by UV 220 nm, t_R = 18.3 min) and the reaction mixture (**3n**) was directly treated with several cell lines. MS (ESI$^+$): *m/z* calcd for $C_{74}H_{132}N_{39}O_{13}S$ ([M + 3H]$^{3+}$) 602.3, found 602.7.

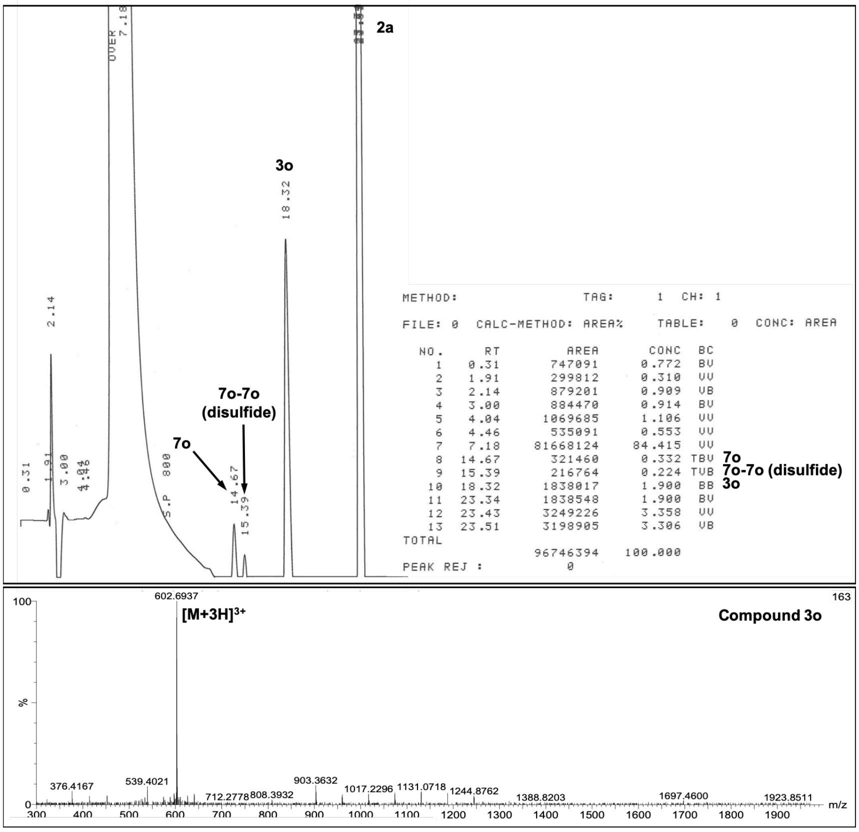

NO.	RT	AREA	CONC	BC	
1	0.31	747091	0.772	BV	
2	1.91	299812	0.310	VV	
3	2.14	879201	0.909	VB	
4	3.00	884470	0.914	BV	
5	4.04	1069685	1.106	VV	
6	4.46	535091	0.553	VV	
7	7.18	81668124	84.415	VV	
8	14.67	321460	0.332	TBV	7o
9	15.39	216764	0.224	TVB	7o-7o (disulfide)
10	18.32	1838017	1.900	BB	3o
11	23.34	1838548	1.900	BV	
12	23.43	3249226	3.358	VV	
13	23.51	3198905	3.306	VB	
TOTAL		96746394	100.000		

Supplementary Figure 13 | HPLC chart and MS spectrum of 3n in the reaction.

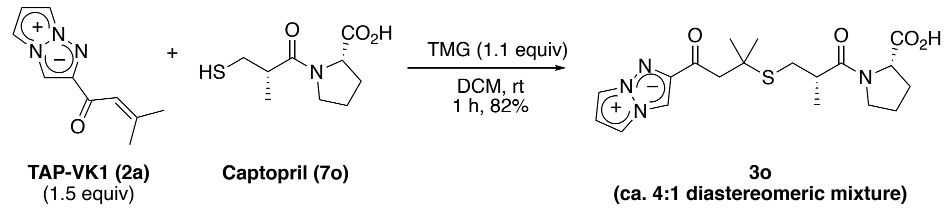

VK1-Captopril (3o): To a solution of TAP-VK1 (**2a**) (13.1 mg, 69.0 μmol) in DCM (0.50 mL) were added Captopril (**7o**) (10.0 mg, 46.0 μmol) and TMG (6.4 μL, 50.6 μmol) at 0 °C, successively. The mixture was stirred at room temperature for 1 h, and the reaction was quenched with AcOH (3.7 μL, 77.7 μmol). The mixture was extracted with CH_2Cl_2 (x4). The combined organic layers were washed with brine, dried over anhydrous Na_2SO_4, filtered, and concentrated under reduced pressure. The residue was purified by silica gel flash column chromatography (MeOH/CH_2Cl_2 = 5/95 to 10/90) to give the inseparable diastereomeric mixture of VK1-Captopril (**3o**) (15.3 mg, 37.6 μmol, 82%, dr ca. 4:1 mixture) as a yellow oil. ^{1}H NMR ($CDCl_3$, 500 MHz): δ 7.62 (d, *J* = 1.3 Hz, 1H), 7.45 (br d, *J* = 2.9 Hz, 1H), 7.20 (d, *J* = 2.9 Hz, 1H), 6.77 (t, *J* = 2.9 Hz, 1H), 4.63 (dd, *J* = 8.0, 2.7 Hz, 1H), 3.64-3.46 (m, 2H), 3.28 (d, *J* = 14.9 Hz, 1H), 3.22 (d, *J* = 14.9 Hz, 1H), 2.97 (m, 1H), 2.82-2.64 (m, 2H), 2.10-1.90 (m, 3H), 1.48 (s, 6H), 1.23 (d, *J* = 6.6 Hz, 3H). ^{13}C NMR ($CDCl_3$, 125 MHz): 193.7, 178.1, 175.1, 147.7, 111.5, 102.6, 101.9, 98.5, 60.6, 49.7, 48.3, 45.0, 38.9, 32.0, 29.2, 29.1, 27.2, 25.1, 18.0: IR (KBr): 3477, 3153, 2970, 2928, 1732, 1682, 1633, 1614, 1538, 1188. HRMS (ESI): m/z [M-H]$^-$ calcd for $[C_{19}H_{25}N_4O_4S]^-$ 405.1597, found 405.1597. UV/Vis (CH_2Cl_2): λ_{max} (log ε) = 395 (3.06), 284 (3.95) nm. FL (CH_2Cl): λ_{max} = 506 nm; Φ_F = 0.014 (reference to 9,10-DPA; excited at 360 nm).

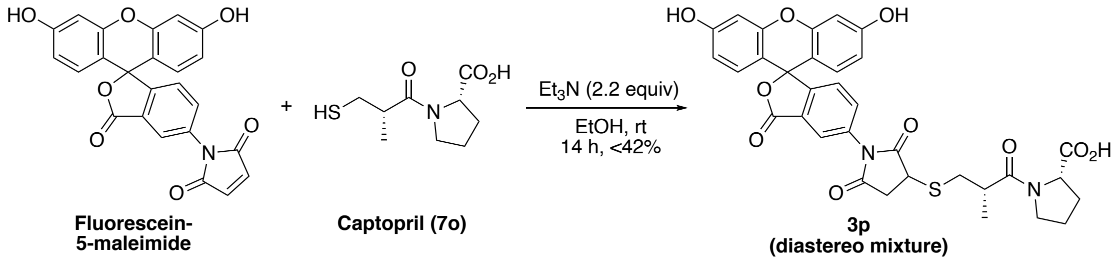

Fluorecein-Captopril (3p): To a solution of Fluorecein-5-maleimide (4.2 mg, 9.8 μmol) in EtOH (0.40 mL) were added Captopril (**7o**) (2.0 mg, 9.2 μmol) and E_3N (2.8 μL, 27.6 μmol) at 0 °C, successively. The mixture was stirred at room temperature for 14 h, and the reaction was quenched with saturated AcOH (1.7 μL, 27.6 μmol). The solvent was removed *in vacuo*, and the residue was purified by reverse-phase column chromatography (Wakogel 50C18, MeCN/H_2O = 10/90 to 75/25) to give the mixture containing Fluorescein-Captopril (**3p**) diastereomers (2.5 mg, <42%), and this mixture was used for ACE assay without further purification. HRMS (ESI): m/z [M-H]$^-$ calcd for $[C_{33}H_{27}N_2O_{10}S]^-$ 643.1386, found 643.1382.

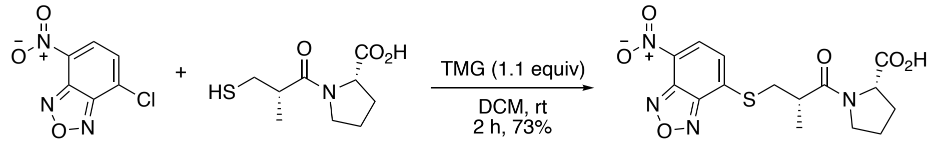

NBD-Cl Captopril (7o) 3q (ca. 8:1 diastereomeric mixture)

NBD-Captopril (3q): To a solution of Captopril (**7o**) (10.6 mg, 48.8 µmol) in DCM (0.50 mL) were added 4-Chloro-7-nitro-2,1,3-benzoxadiazole (NBD-Cl) (14.6 mg, 73.0 µmol) and TMG (6.4 µL, 50.6 µmol) at 0 °C, successively. The mixture was stirred at room temperature for 2 h, and the reaction was quenched with AcOH (10.0 µL, 77.7 µmol). The mixture was extracted with CH_2Cl_2 (x4). The combined organic layers were washed with brine, dried over anhydrous Na_2SO_4, filtered, and concentrated under reduced pressure. The residue was purified by preparative TLC ($MeOH/CH_2Cl_2$ = 10/90) to give the inseparable diastereomeric mixture of NBD-Captopril (**3q**) (12.7 mg, 33.4 µmol, 73%, dr ca. 8:1 mixture) as a yellow oil. HRMS (ESI): m/z $[M+Na]^+$ calcd for $[C_{15}H_{16}N_4O_6SNa]^+$ 403.0688, found 403.0692.

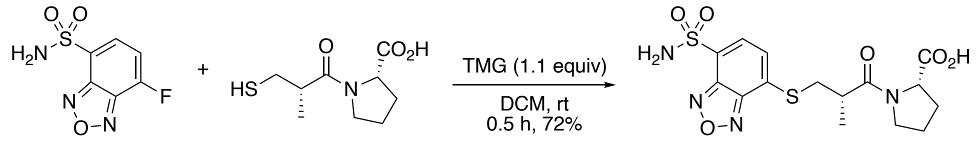

ABD-F Captopril (7o) 3r (ca. 1.3:1 diastereomeric mixture)

ABD-Captopril (3r): To a solution of Captopril (**7o**) (5.0 mg, 23.0 µmol) in DCM (0.23 mL) were added 4-(aminosulfonyl)-7-fluoro-2,1,3-benzoxadiazole (ABD-F) (7.5 mg, 34.5 µmol) and TMG (3.2 µL, 25.3 µmol) at 0 °C, successively. The mixture was stirred at room temperature for 0.5 h, and the reaction was quenched with AcOH (5.0 µL, 38.9 µmol). The mixture was extracted with CH_2Cl_2 (x4). The combined organic layers were washed with brine, dried over anhydrous Na_2SO_4, filtered, and concentrated under reduced pressure. The residue was purified by preparative TLC ($MeOH/CH_2Cl_2$ = 10/90) to give the inseparable diastereomeric mixture of ABD-Captopril (**3r**) (6.9 mg, 16.6 µmol, 72%, dr ca. 8:1 mixture) as a yellow oil. HRMS (ESI): m/z $[M+H]^+$ calcd for $[C_{15}H_{19}N_4O_6S_2]^+$ 415.0746, found 415.0747.

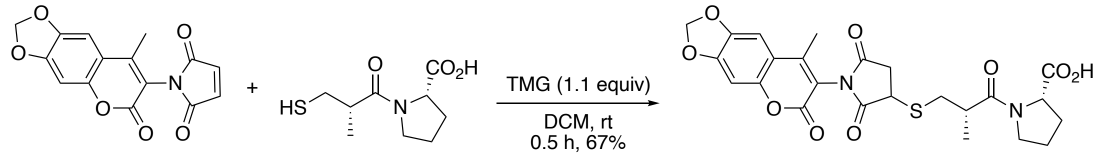

Coumarin-maleimide Captopril (7o) 3s (ca. 2:1 diastereomeric mixture)

Coumarin-Captopril (3s): To a solution of Captopril (**7o**) (2.4 mg, 11.0 µmol) in DCM (0.23 mL) were added 6,7-methylenedioxy-4-methyl-3-maleimidocoumarin (Coumarin-maleimide) (5.0 mg,

16.5 μmol) and TMG (1.6 μL, 12.1 μmol) at 0 °C, successively. The mixture was stirred at room temperature for 0.5 h, and the reaction was quenched with AcOH (5.0 μL, 38.9 μmol). The mixture was extracted with CH_2Cl_2 (x4). The combined organic layers were washed with brine, dried over anhydrous Na_2SO_4, filtered, and concentrated under reduced pressure. The residue was purified by preparative TLC (MeOH/CH_2Cl_2 = 10/90) to give the inseparable diastereomeric mixture of Coumarin-Captopril (**3r**) (4.3 mg, 8.3 μmol, 67%, dr ca. 2:1 mixture) as a white wax. HRMS (ESI): m/z $[M+H]^+$ calcd for $[C_{24}H_{25}N_2O_9S]^+$ 517.1281, found 517.1284.

Compound 1a

(^{1}H NMR, 400 MHz in $CDCl_3$)

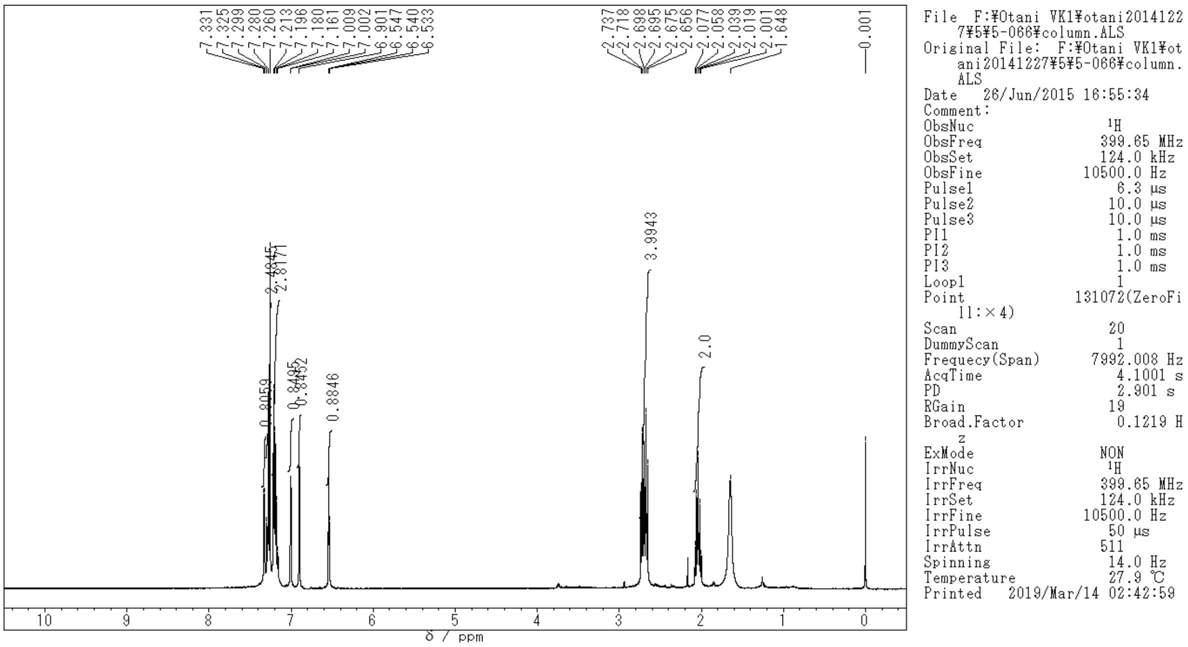

(^{13}C NMR, 100 MHz in $CDCl_3$)

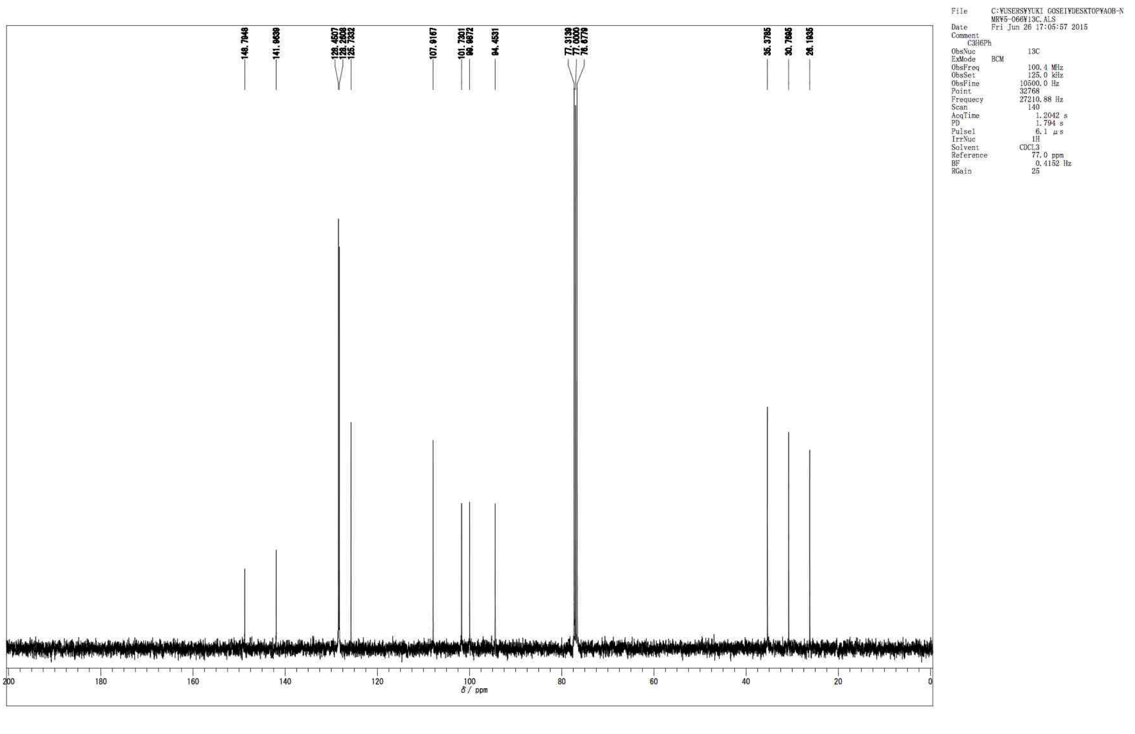

Supplementary Figure 14 | ^{1}H and ^{13}C NMR spectra of Compound 1a.

Compound 1b

(^{1}H NMR, 400 MHz in CDCl$_3$)

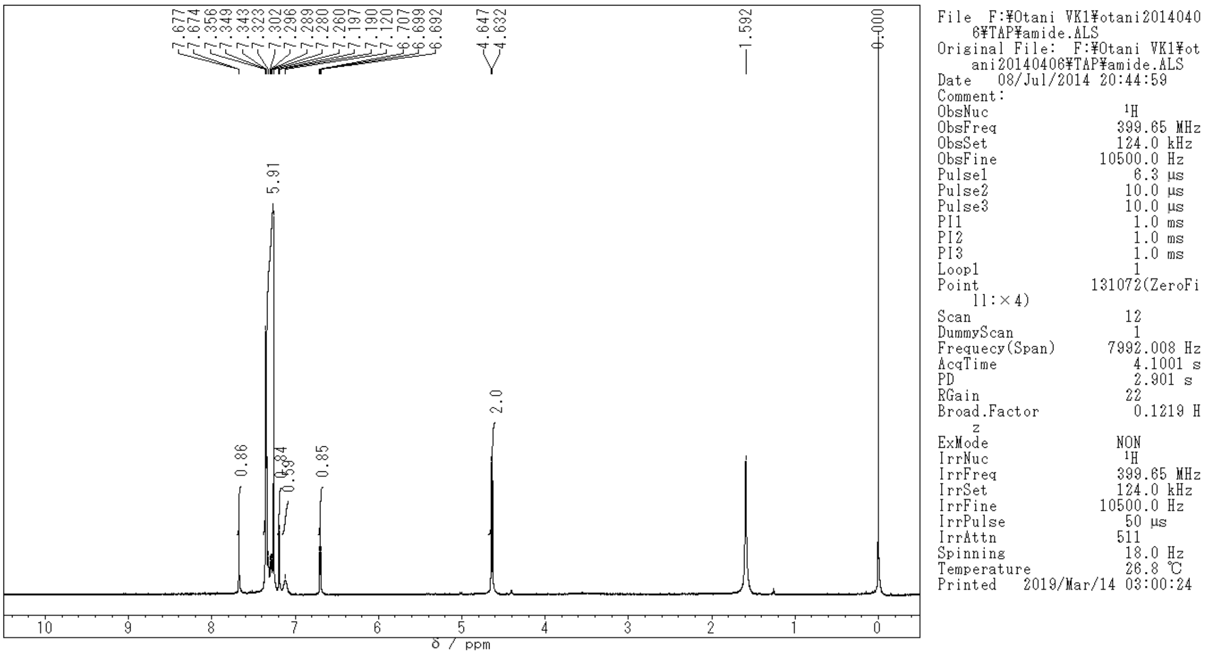

(^{13}C NMR, 100 MHz in CDCl$_3$)

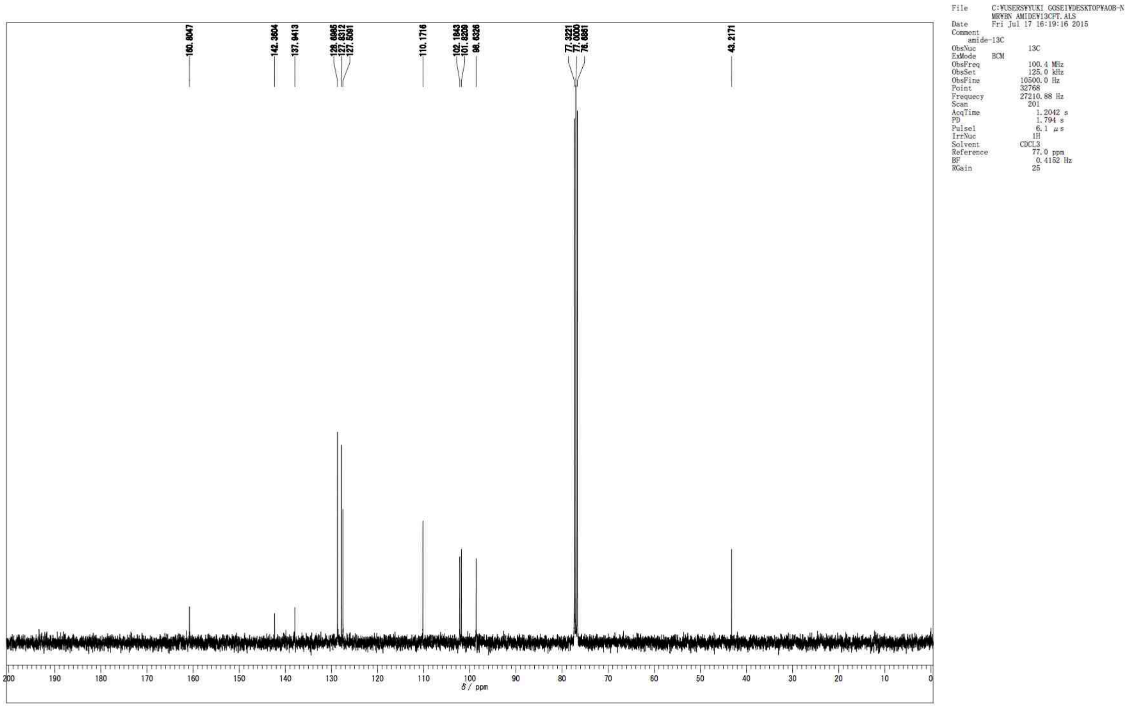

Supplementary Figure 15 | ^{1}H and ^{13}C NMR spectra of Compound 1b.

Compound **1c**

(^{1}H NMR, 400 MHz in $CDCl_3$)

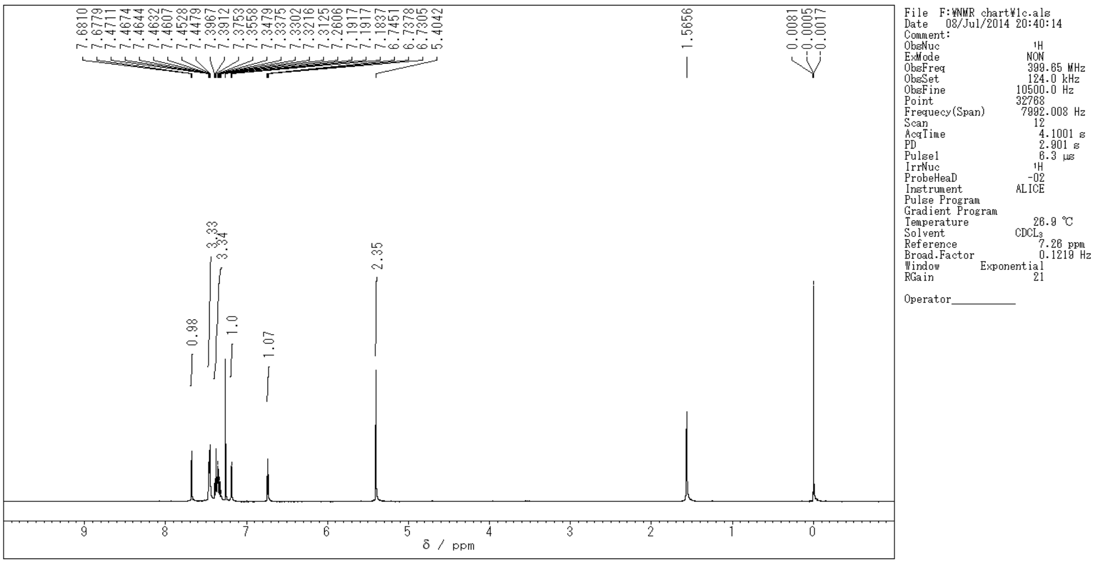

(^{13}C NMR, 100 MHz in $CDCl_3$)

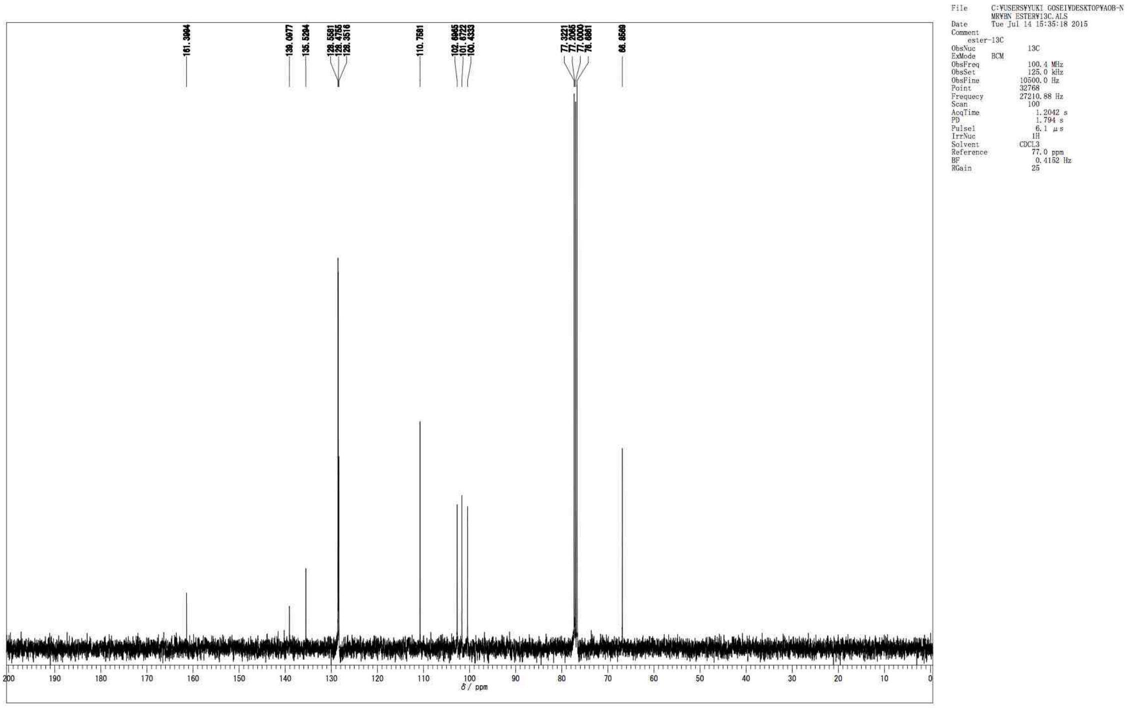

Supplementary Figure 16 | ^{1}H and ^{13}C NMR spectra of Compound 1c.

Compound **1d**

(^{1}H NMR, 400 MHz in $CDCl_3$)

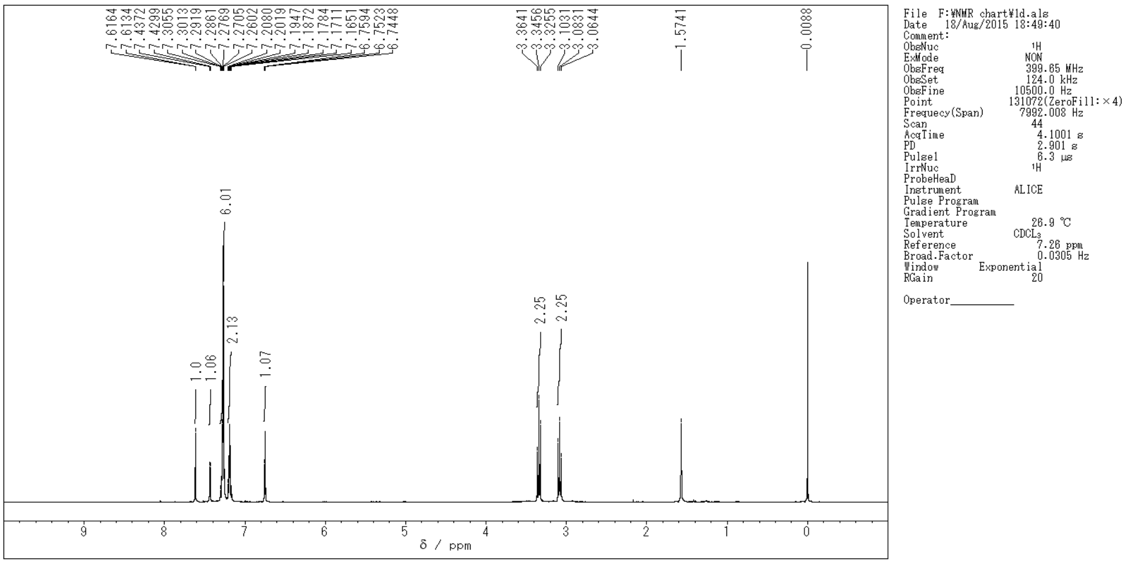

(^{13}C NMR, 100 MHz in $CDCl_3$)

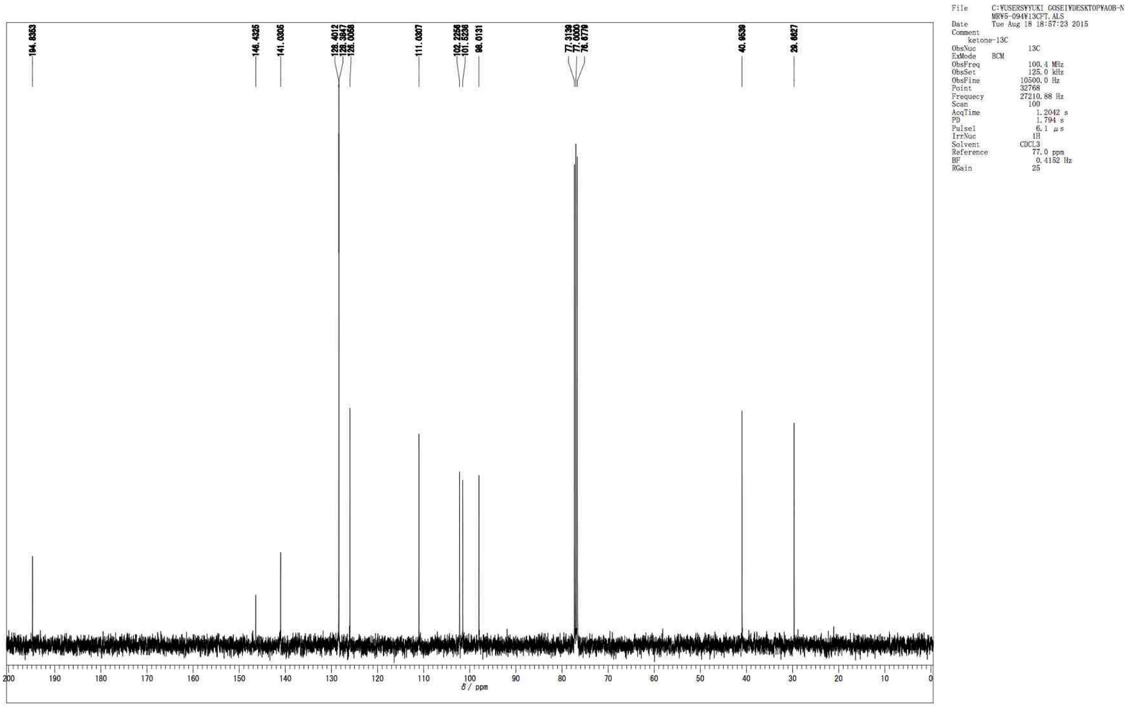

Supplementary Figure 17 | ^{1}H and ^{13}C NMR spectra of Compound 1d.

Compound **5**

(^{1}H NMR, 400 MHz in $CDCl_3$)

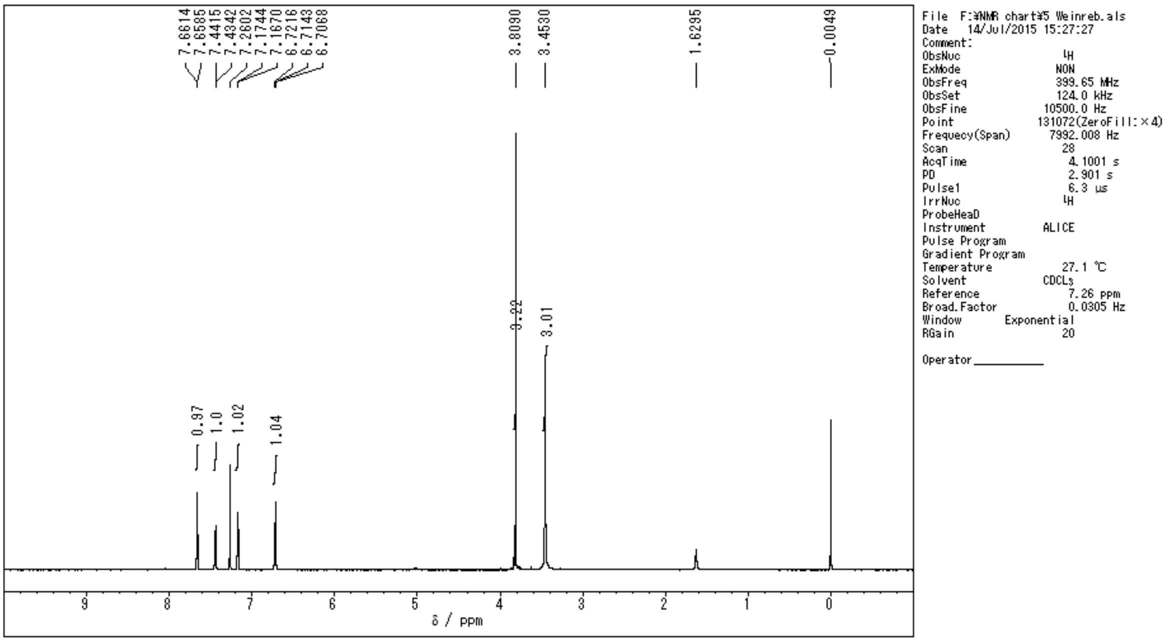

(^{13}C NMR, 100 MHz in $CDCl_3$)

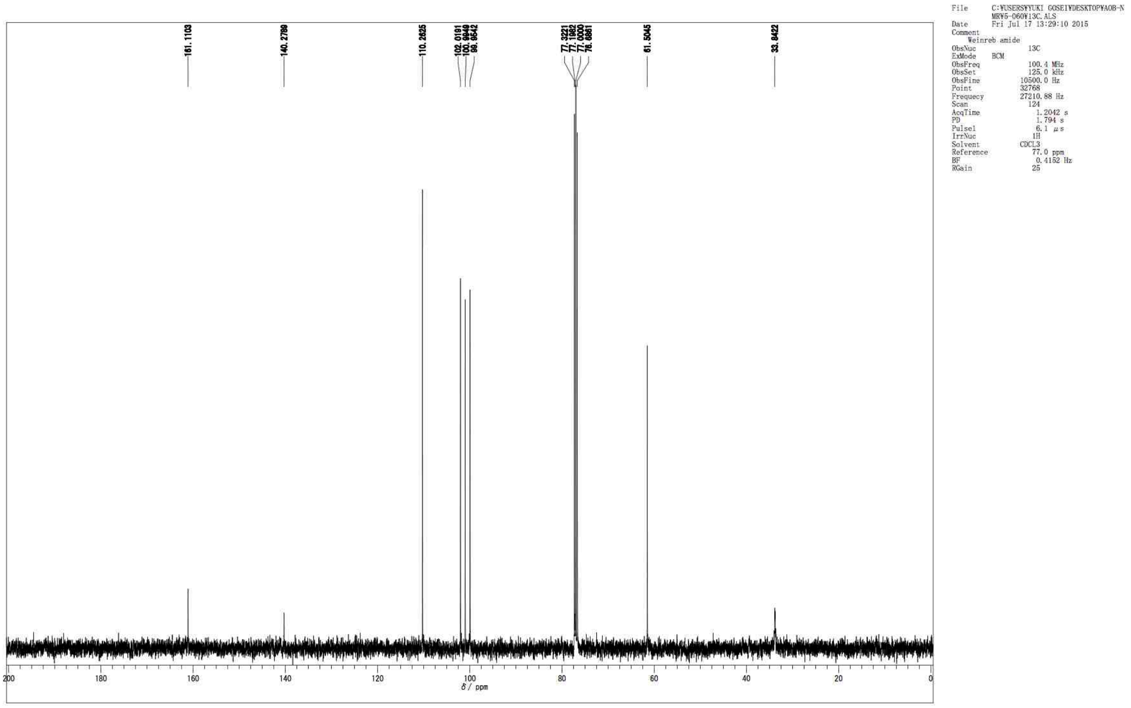

Supplementary Figure 18 | ^{1}H and ^{13}C NMR spectra of Compound 5.

Compound **S3a**

(^{1}H NMR, 400 MHz in CDCl$_3$)

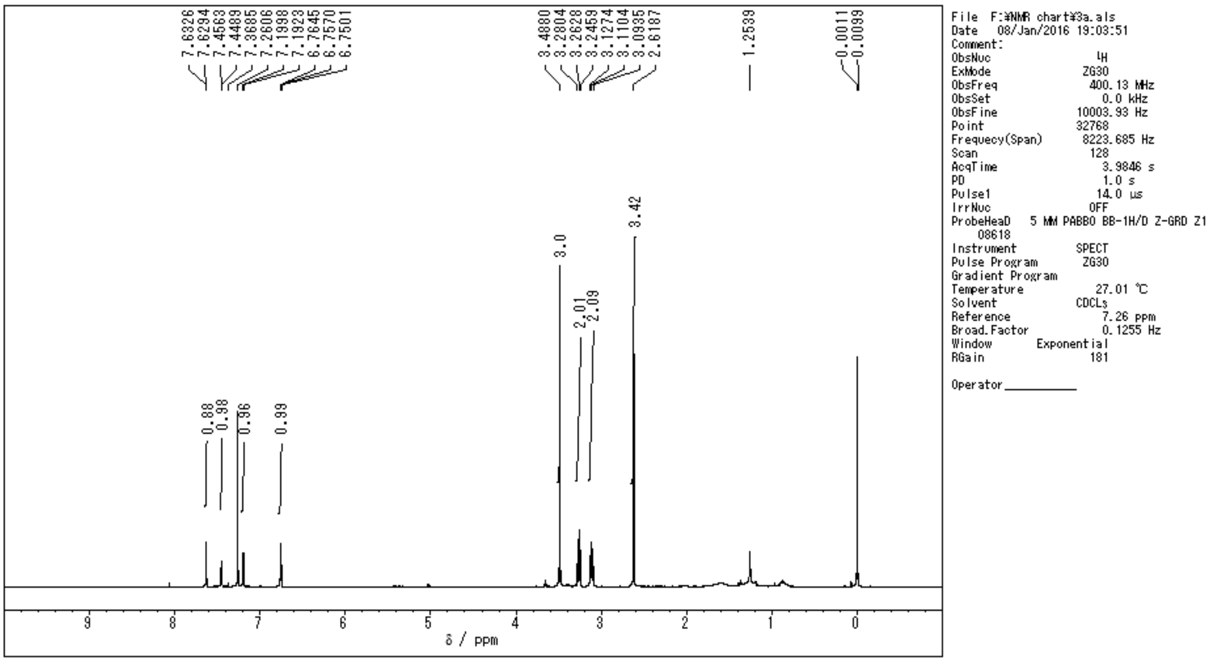

(^{1}H NMR, 125 MHz in CDCl$_3$)

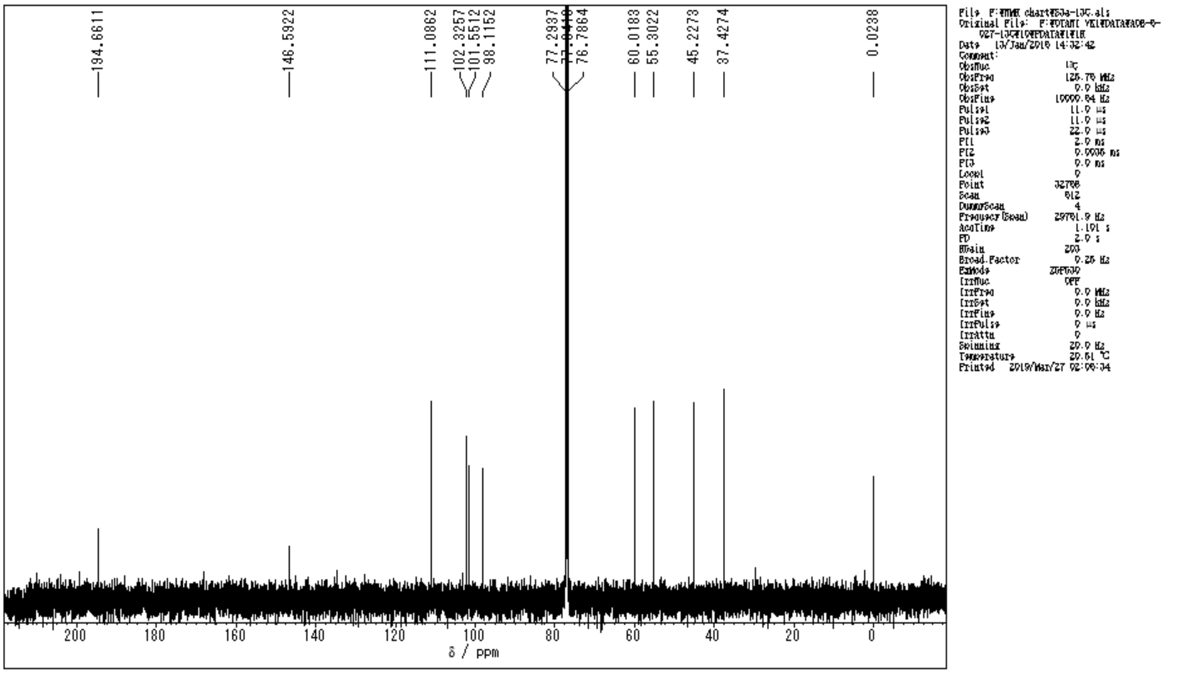

Supplementary Figure 19 | ^{1}H and ^{13}C NMR spectra of Compound S3a.

Compound **S3b**

(^{1}H NMR, 500 MHz in $CDCl_3$)

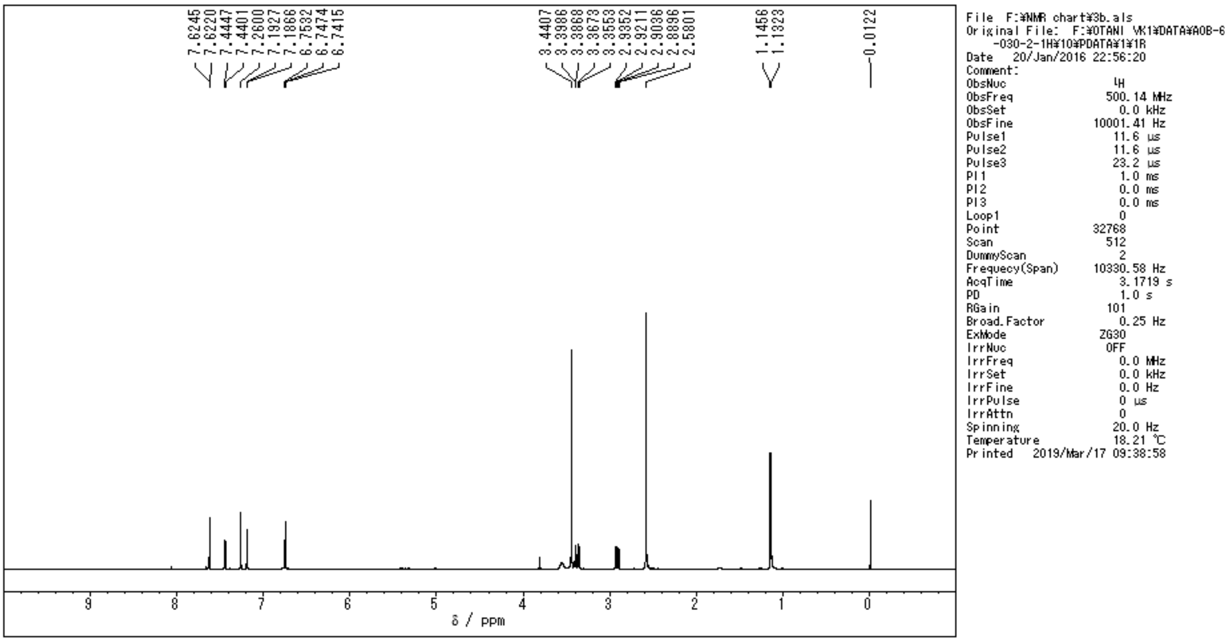

(^{13}C NMR, 125 MHz in $CDCl_3$)

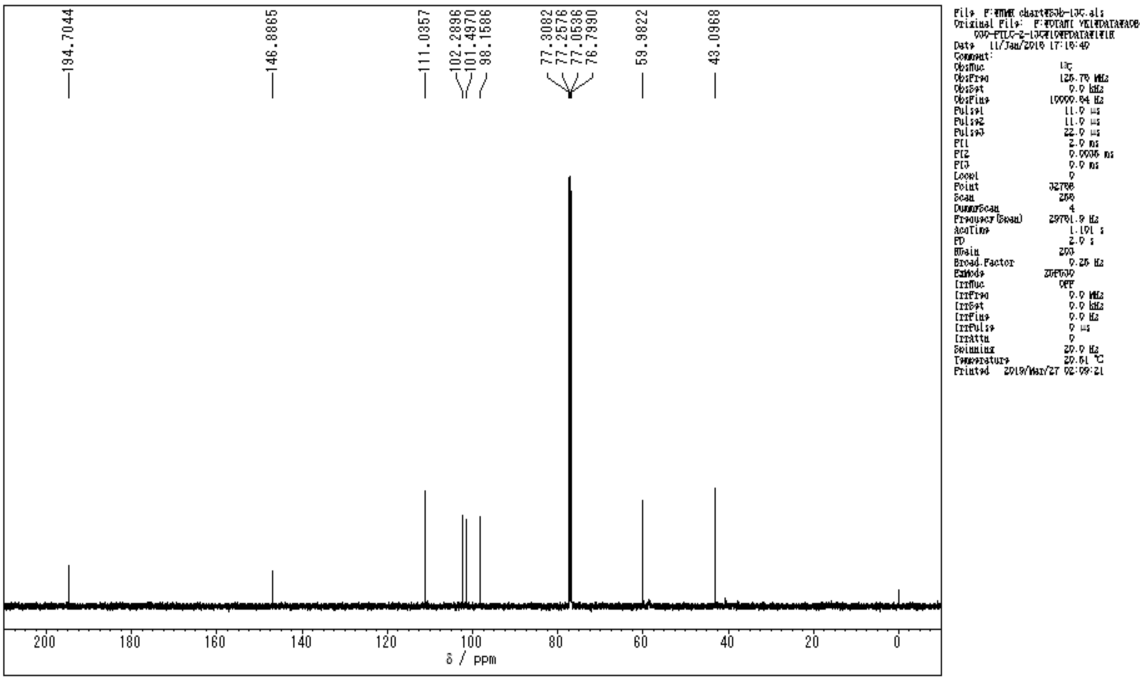

Supplementary Figure 20 | ^{1}H and ^{13}C NMR spectra of Compound S3b.

Compound **2a**

(^{1}H NMR, 400 MHz in $CDCl_3$)

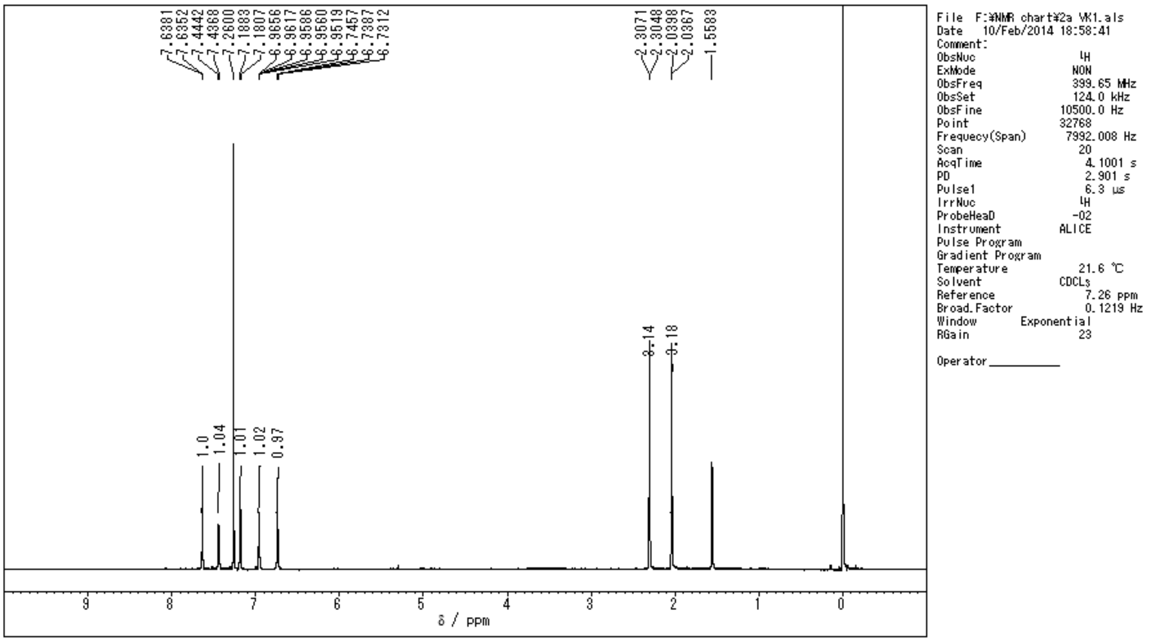

(^{13}C NMR, 100 MHz in $CDCl_3$)

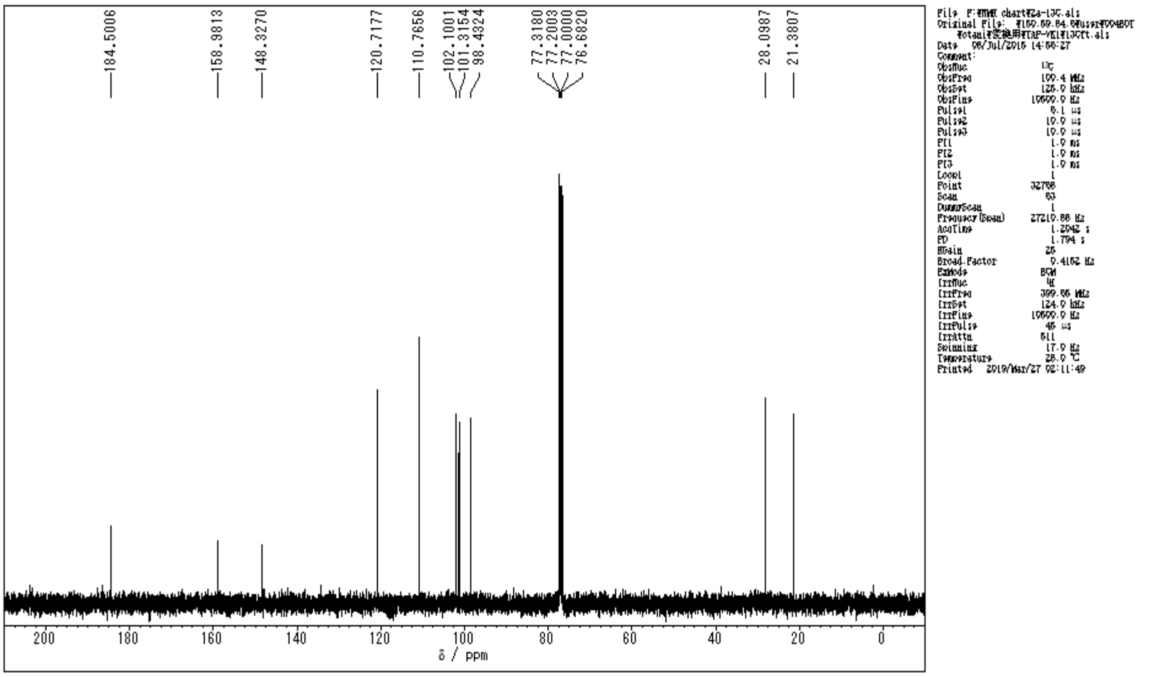

Supplementary Figure 21 | ^{1}H and ^{13}C NMR spectra of Compound 2a.

Compound **3a**

(^{1}H NMR, 400 MHz in CDCl$_3$)

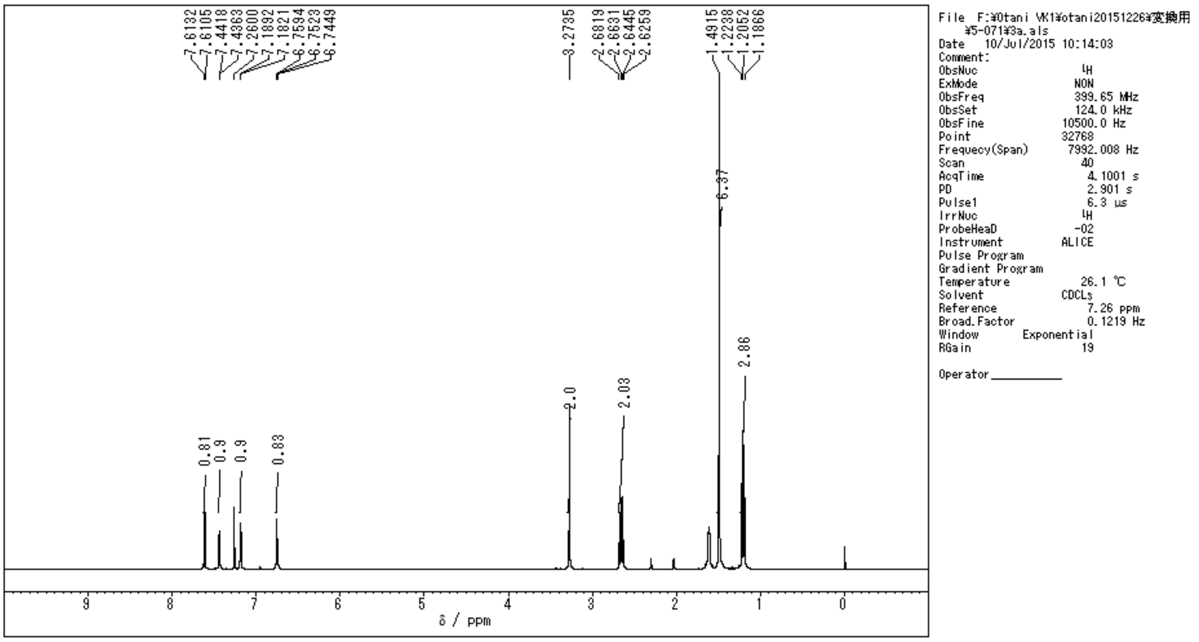

(^{13}C NMR, 100 MHz in CDCl$_3$)

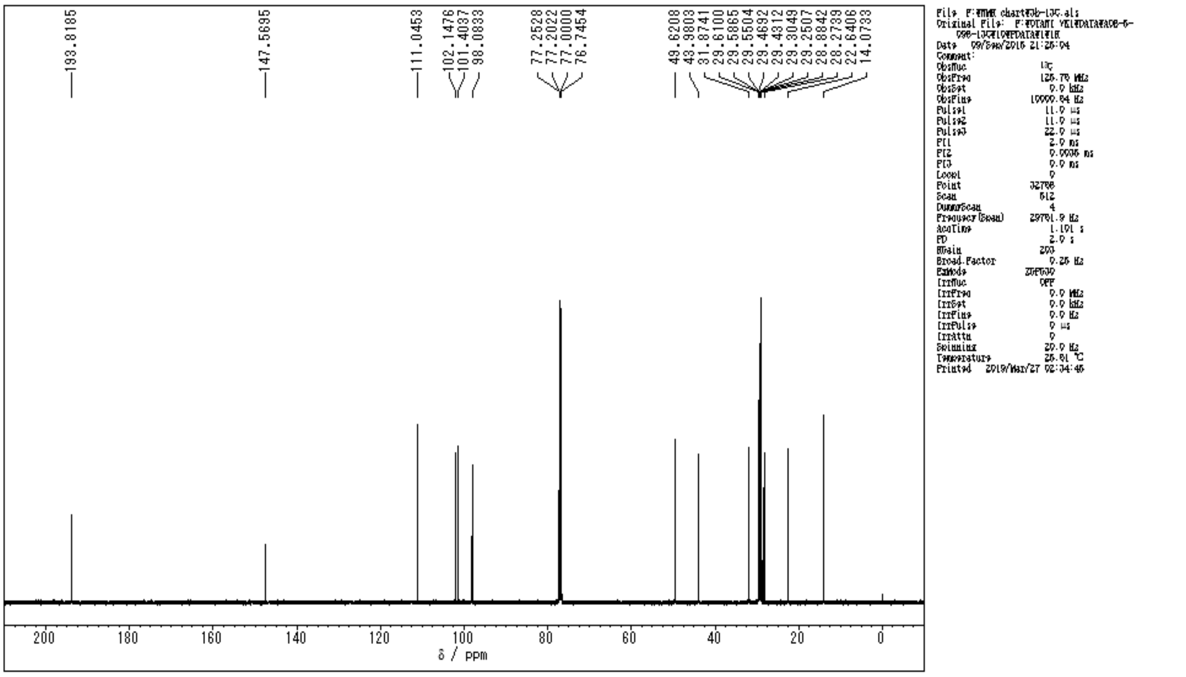

Supplementary Figure 22 | ^{1}H and ^{13}C NMR spectra of Compound 3a.

Compound **3b**

(^{1}H NMR, 400 MHz in CDCl$_3$)

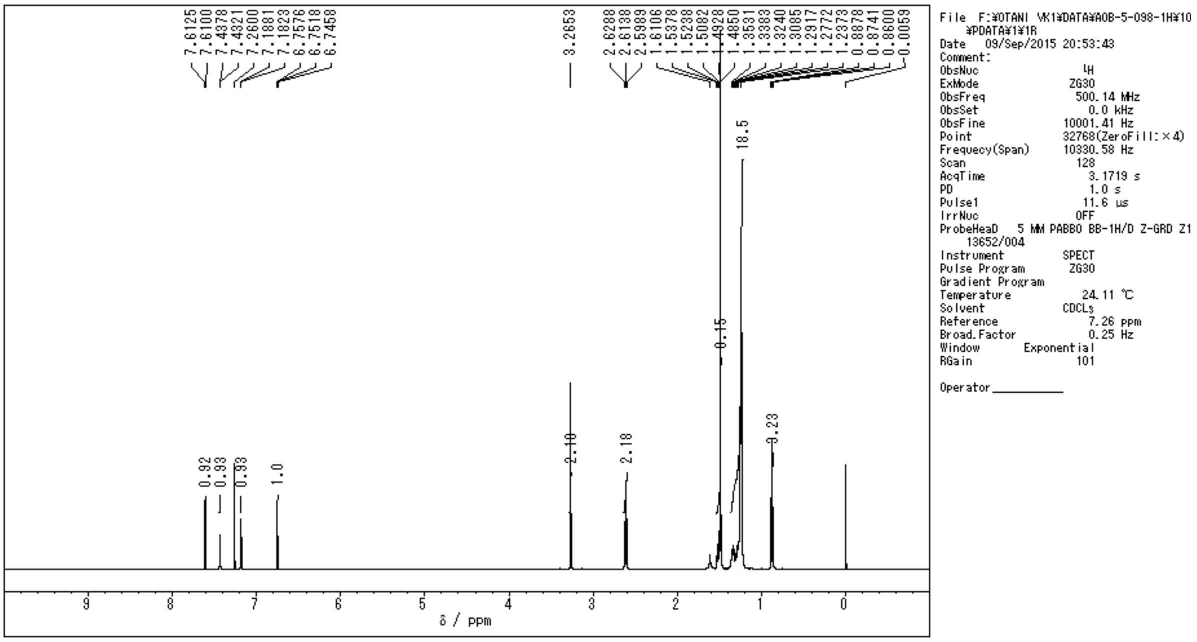

(^{13}C NMR, 125 MHz in CDCl$_3$)

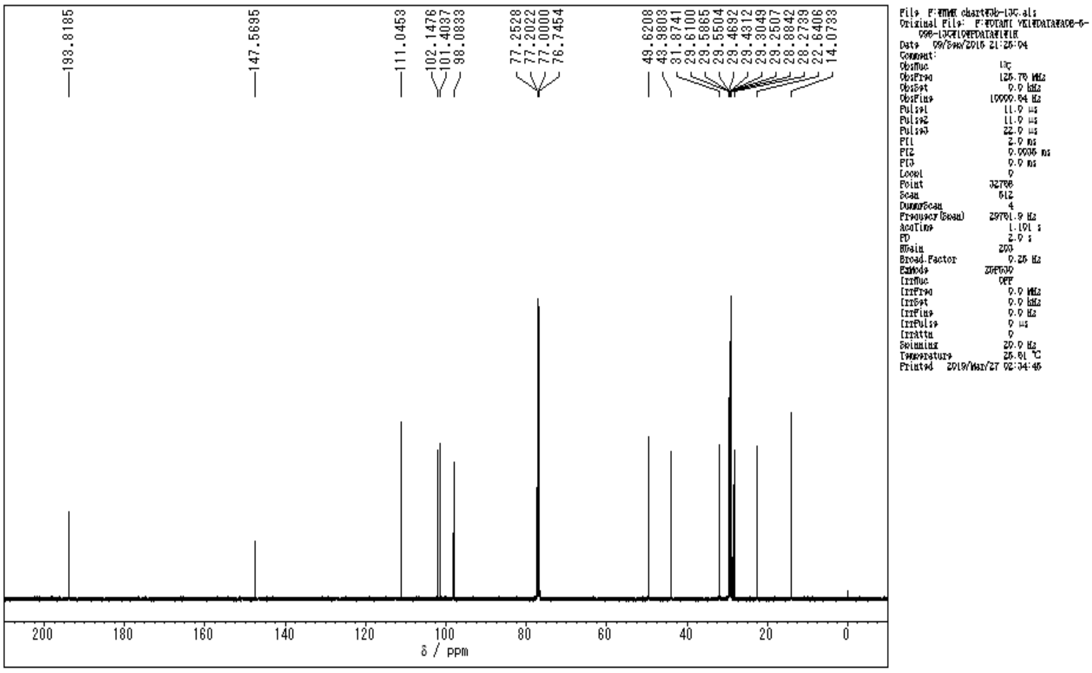

Supplementary Figure 23 | ^{1}H and ^{13}C NMR spectra of Compound 3b.

Compound **3c**

(^{1}H NMR, 400 MHz in $CDCl_3$)

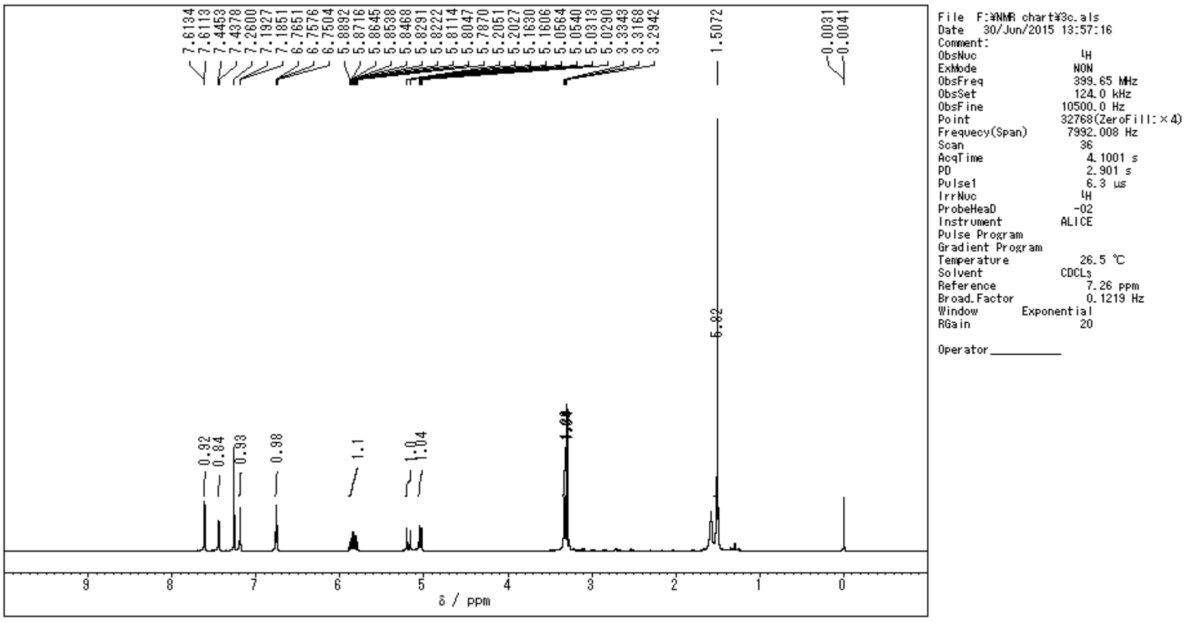

(^{13}C NMR, 125 MHz in $CDCl_3$)

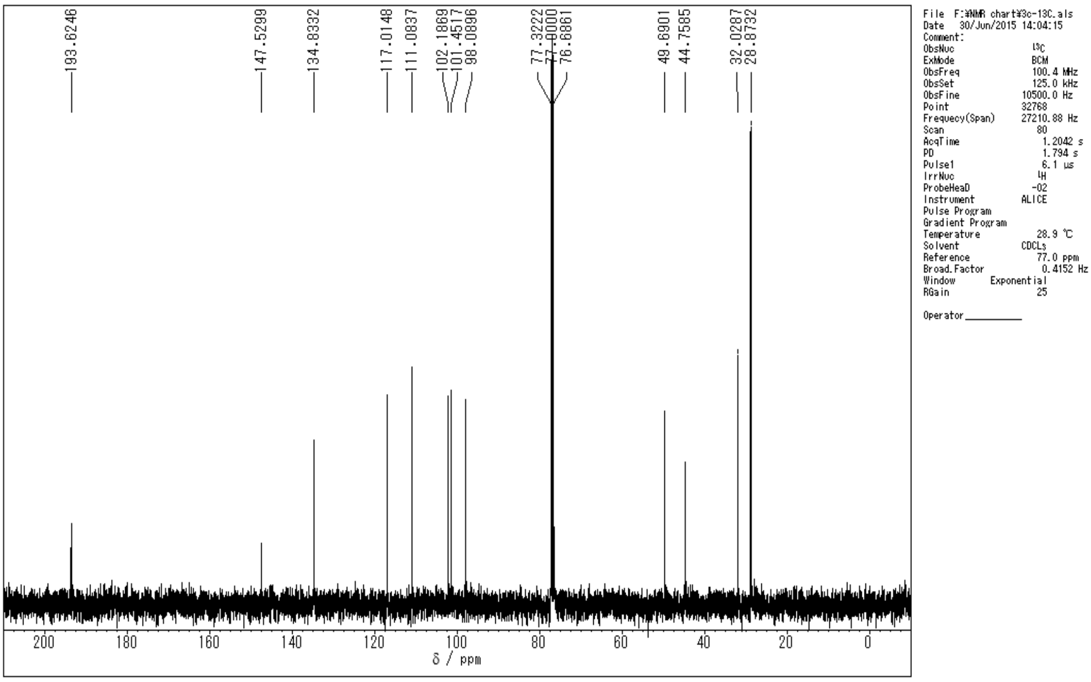

Supplementary Figure 24 | ^{1}H and ^{13}C NMR spectra of Compound 3c.

Compound **3d**

(^{1}H NMR, 400 MHz in CDCl$_3$)

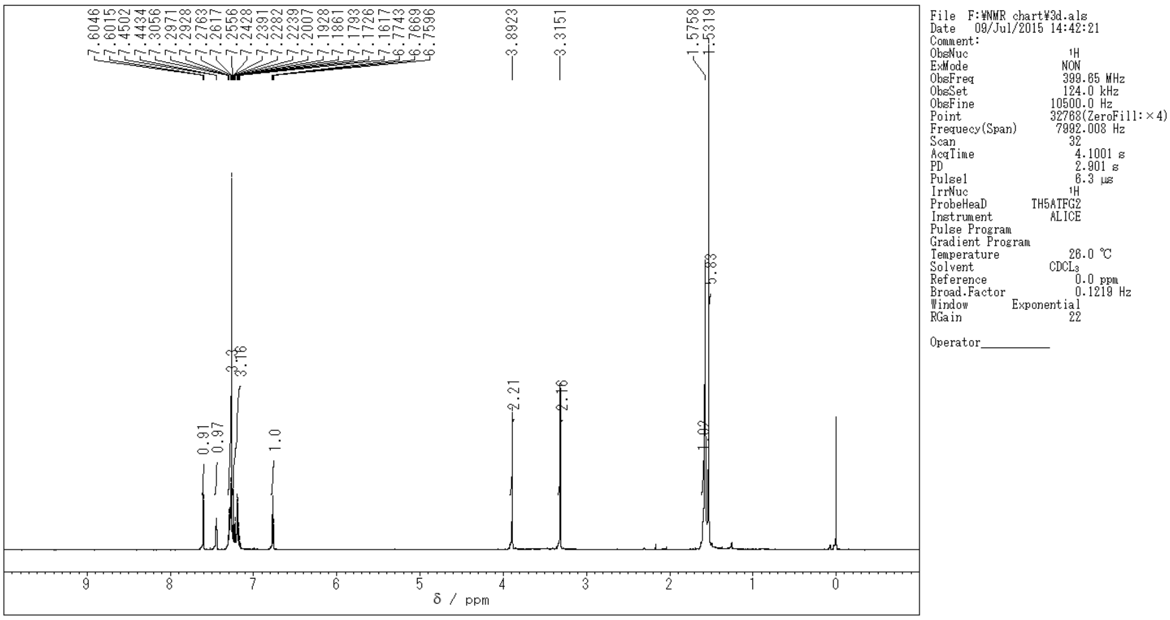

(^{13}C NMR, 100 MHz in CDCl$_3$)

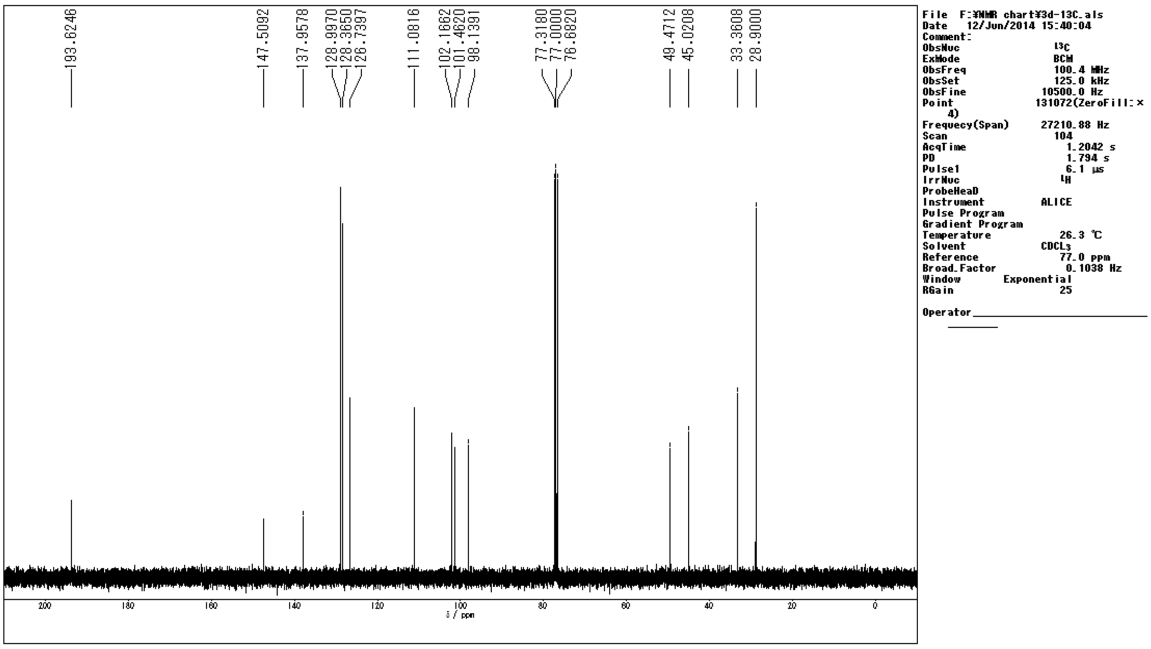

Supplementary Figure 25 | ^{1}H and ^{13}C NMR spectra of Compound 3d.

Compound **3e**

(^{1}H NMR, 400 MHz in $CDCl_3$)

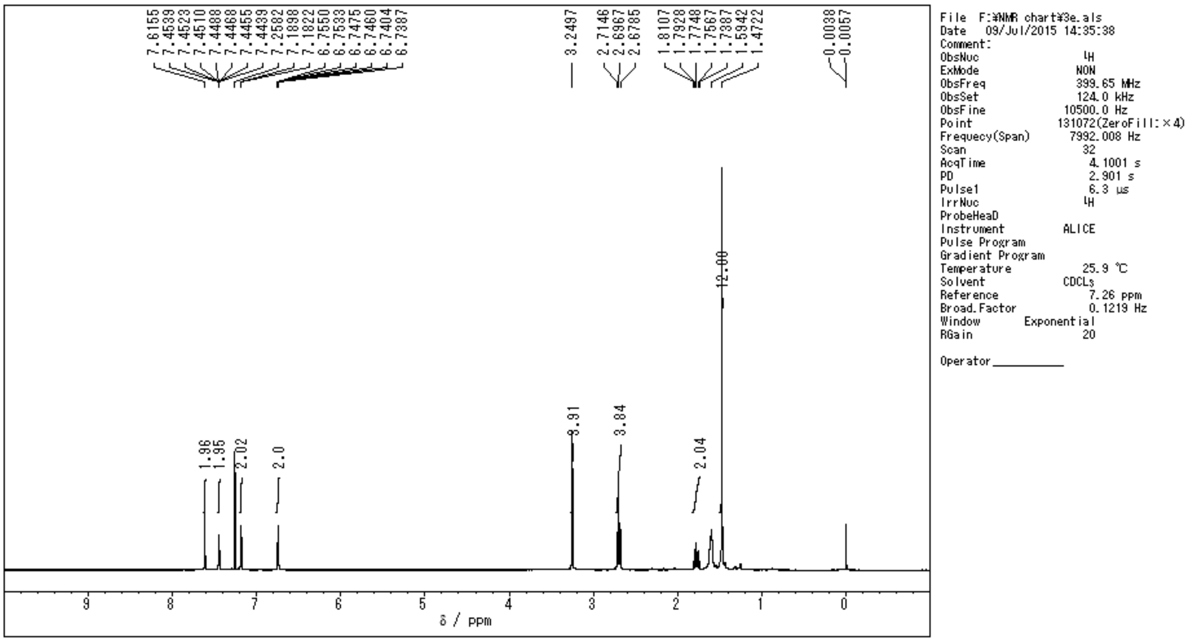

(^{13}C NMR, 100 MHz in $CDCl_3$)

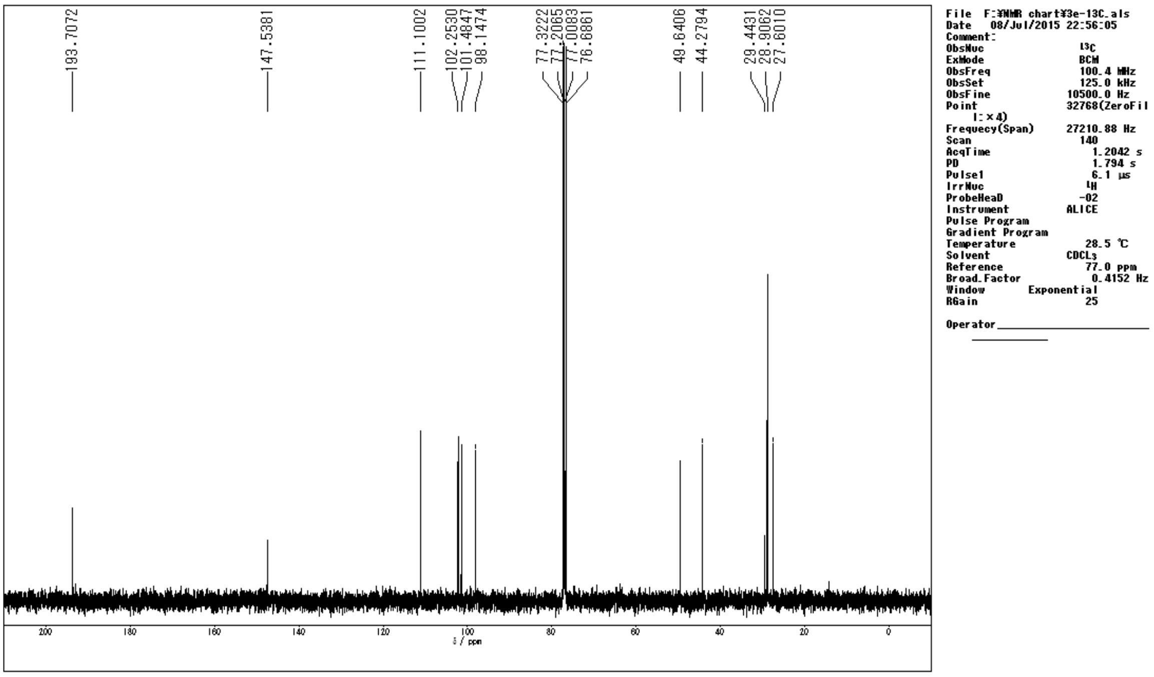

Supplementary Figure 26 | ^{1}H and ^{13}C NMR spectra of Compound 3e.

Compound **3f**

(^{1}H NMR, 400 MHz in $CDCl_3$)

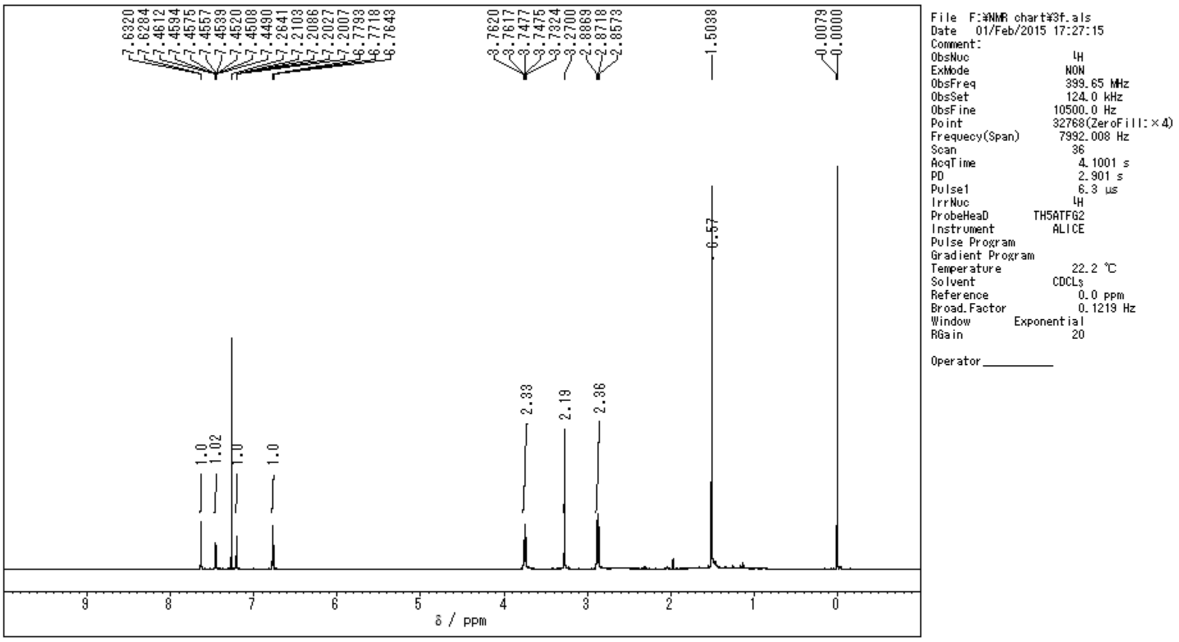

(^{13}C NMR, 100 MHz in $CDCl_3$)

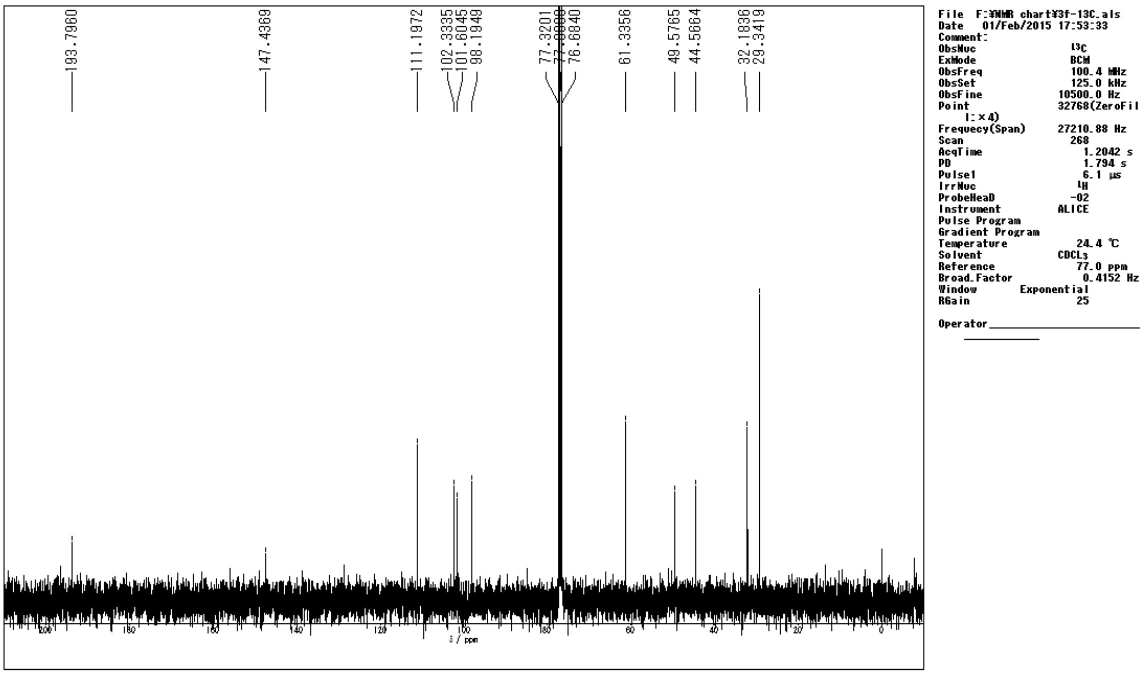

Supplementary Figure 27 | ^{1}H and ^{13}C NMR spectra of Compound 3f.

Compound **3g**

(^{1}H NMR, 400 MHz in CDCl$_3$)

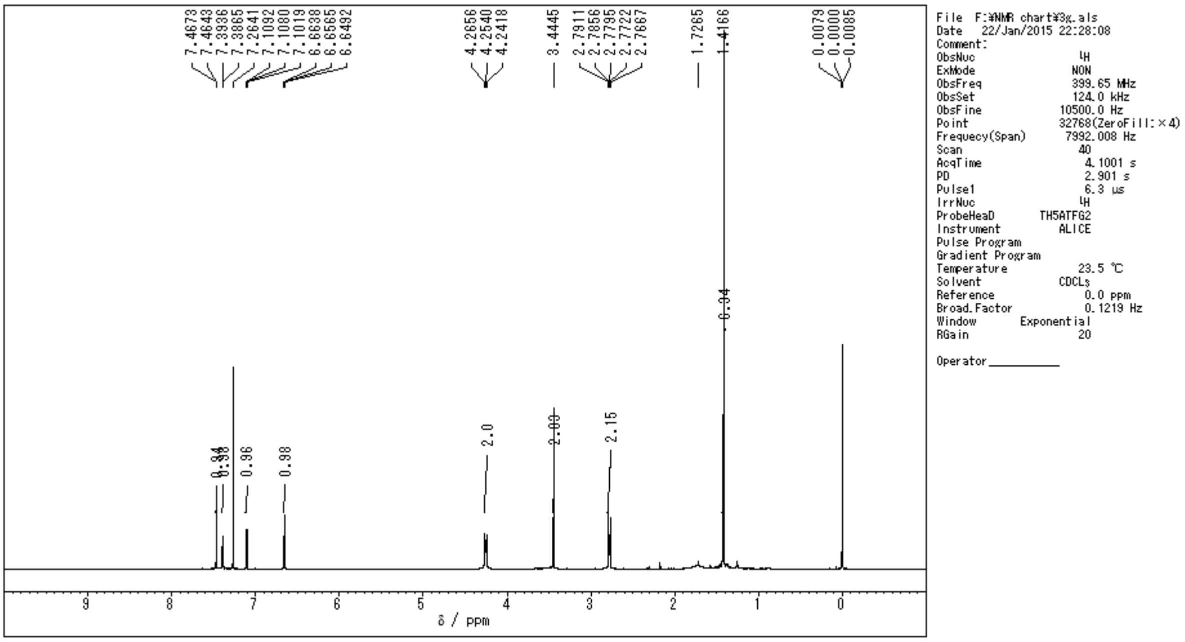

(^{13}C NMR, 100 MHz in CDCl$_3$)

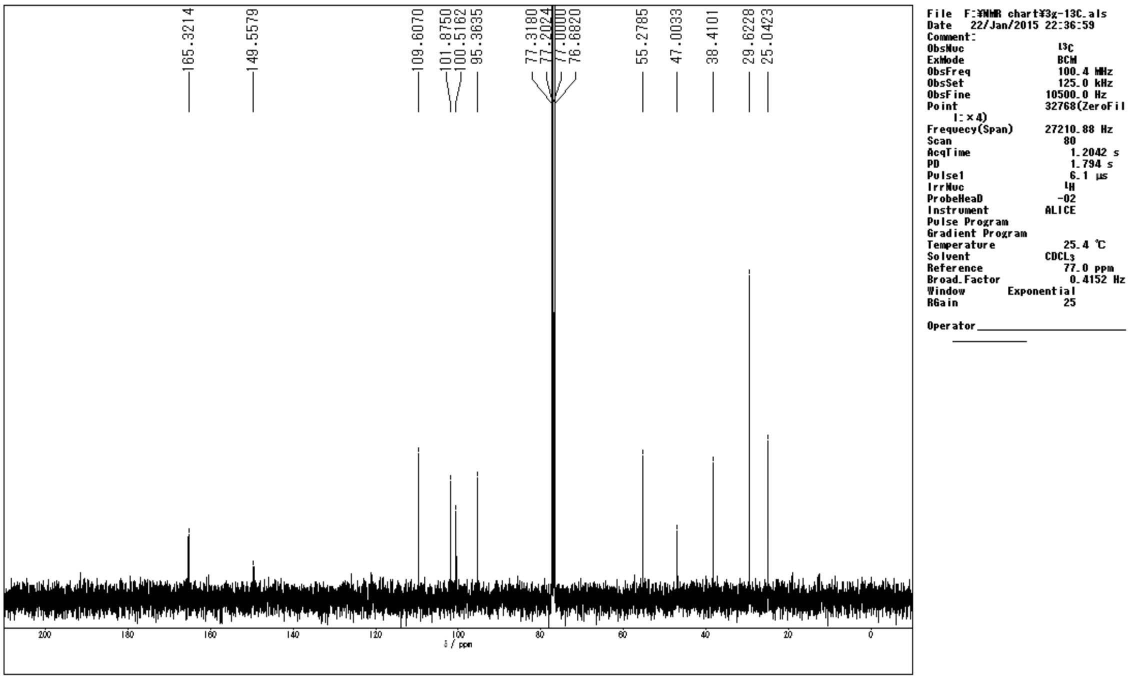

Supplementary Figure 28 | ^{1}H and ^{13}C NMR spectra of Compound 3g.

Compound **S5**

(^{1}H NMR, 500 MHz in $CDCl_3$)

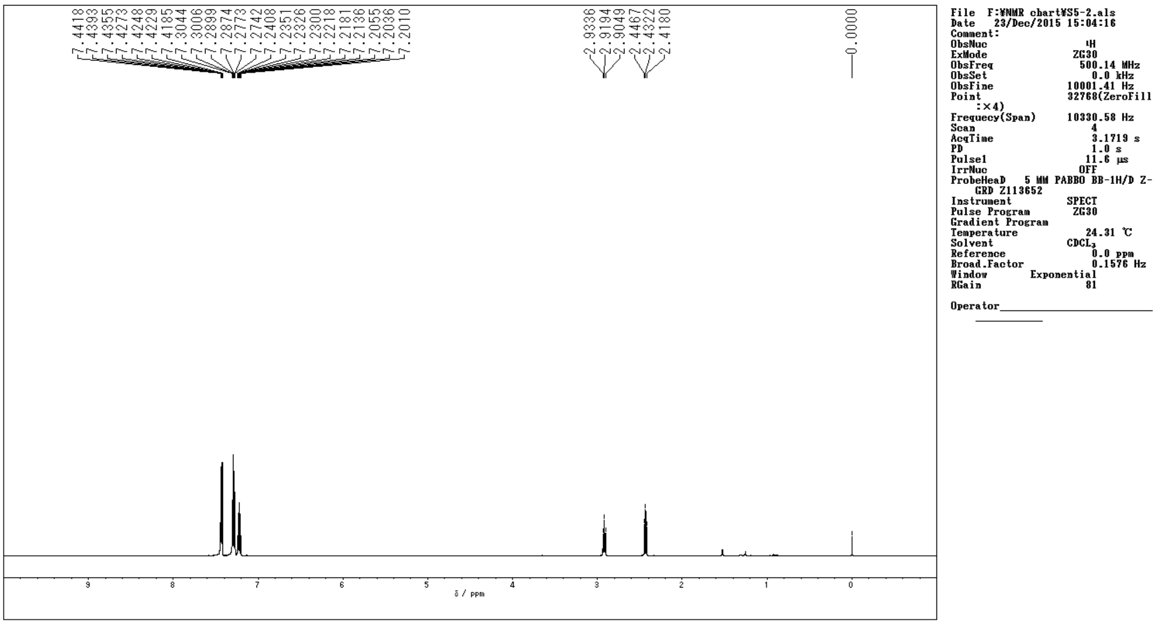

(^{13}C NMR, 125 MHz in $CDCl_3$)

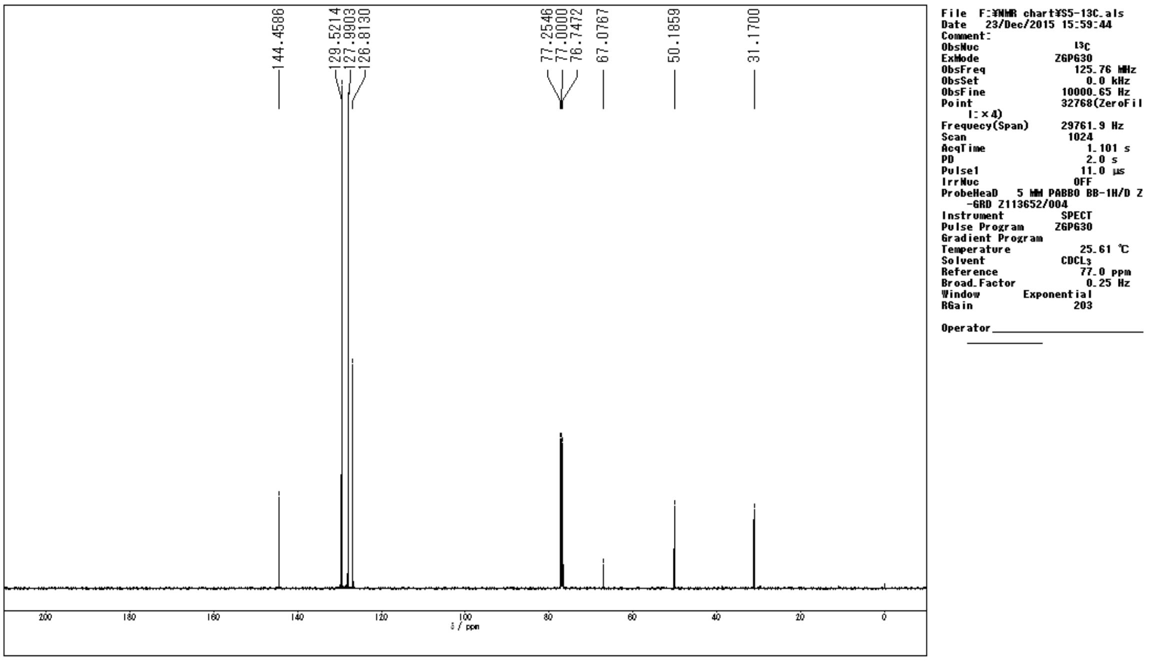

Supplementary Figure 29 | ^{1}H and ^{13}C NMR spectra of Compound S5.

Compound **S6**

(^{1}H NMR, 400 MHz in $CDCl_3$)

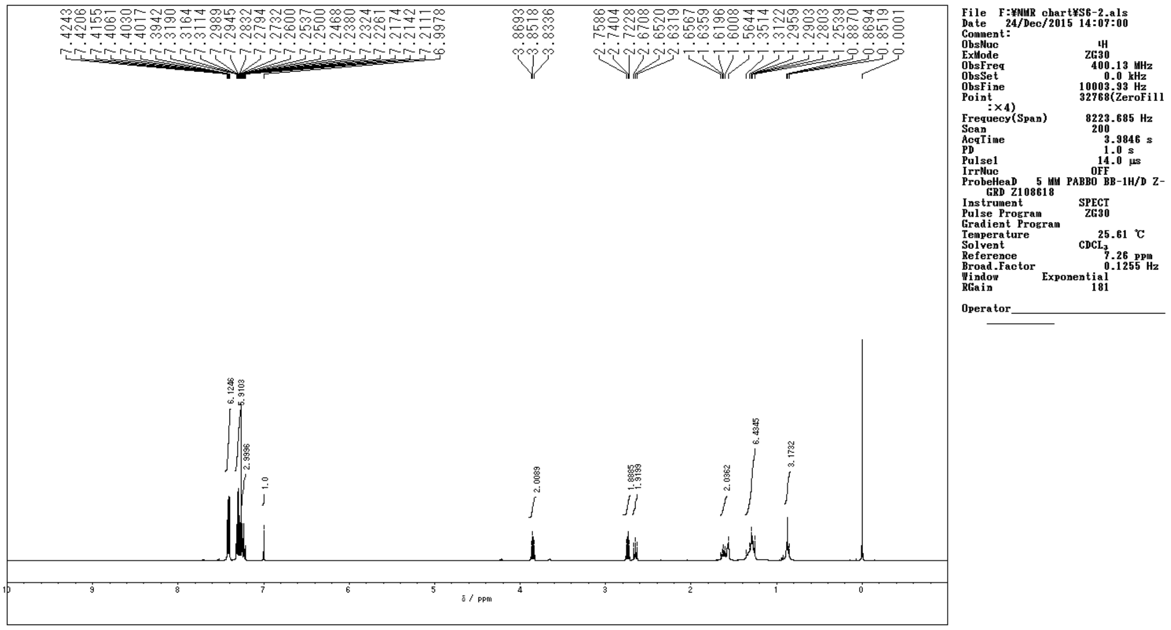

(^{13}C NMR, 125 MHz in $CDCl_3$)

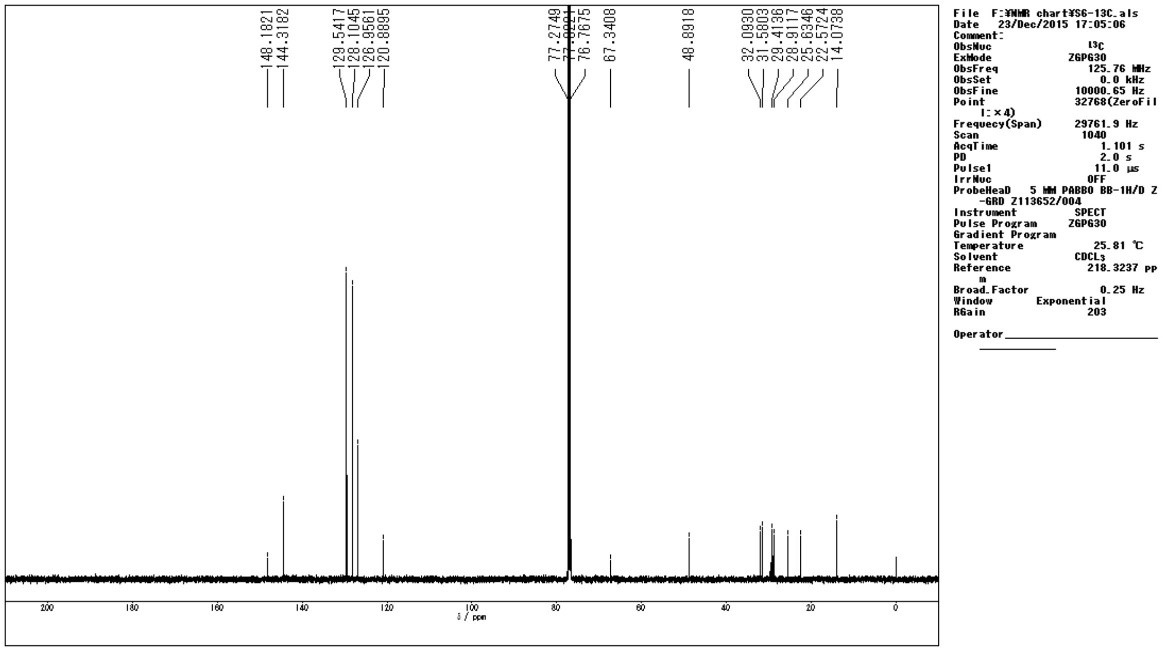

Supplementary Figure 30 | ^{1}H and ^{13}C NMR spectra of Compound S6.

Compound **7h**

(^{1}H NMR, 500 MHz in CDCl$_3$)

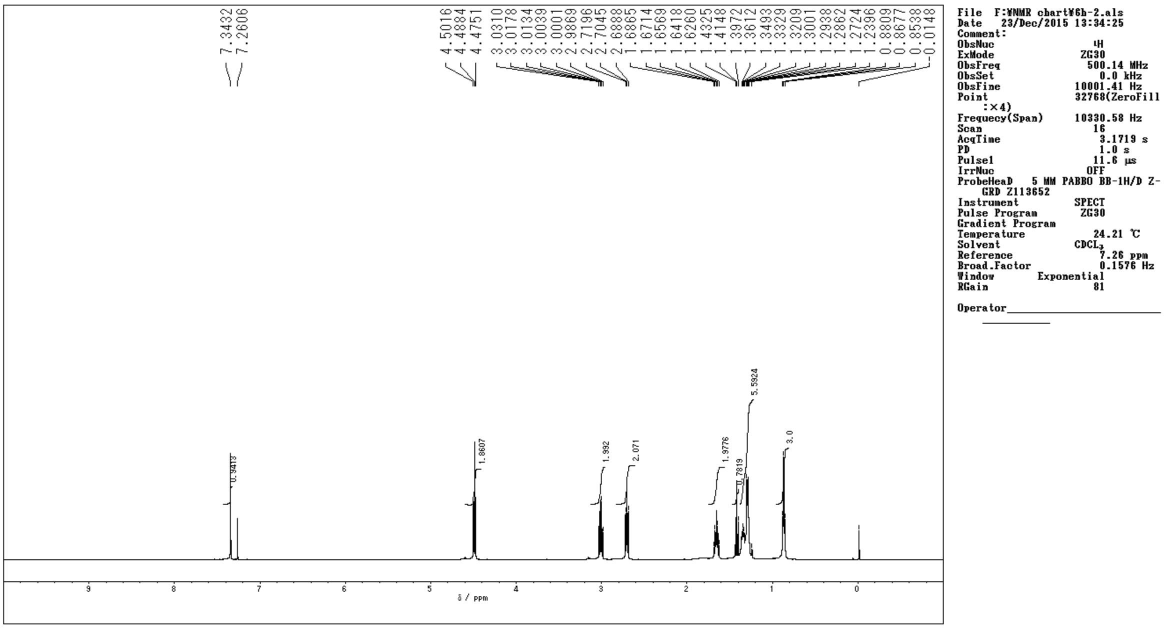

(^{13}C NMR, 125 MHz in CDCl$_3$)

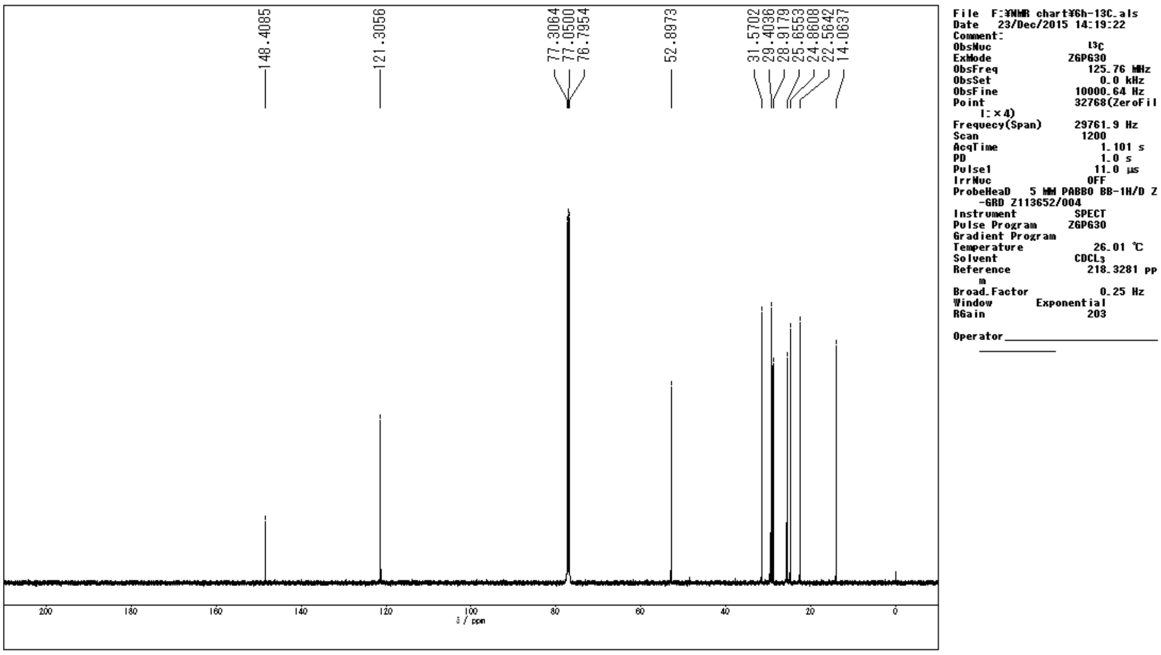

Supplementary Figure 31 | ^{1}H and ^{13}C NMR spectra of Compound 7h.

Compound **3h**

(^{1}H NMR, 500 MHz in CDCl$_3$)

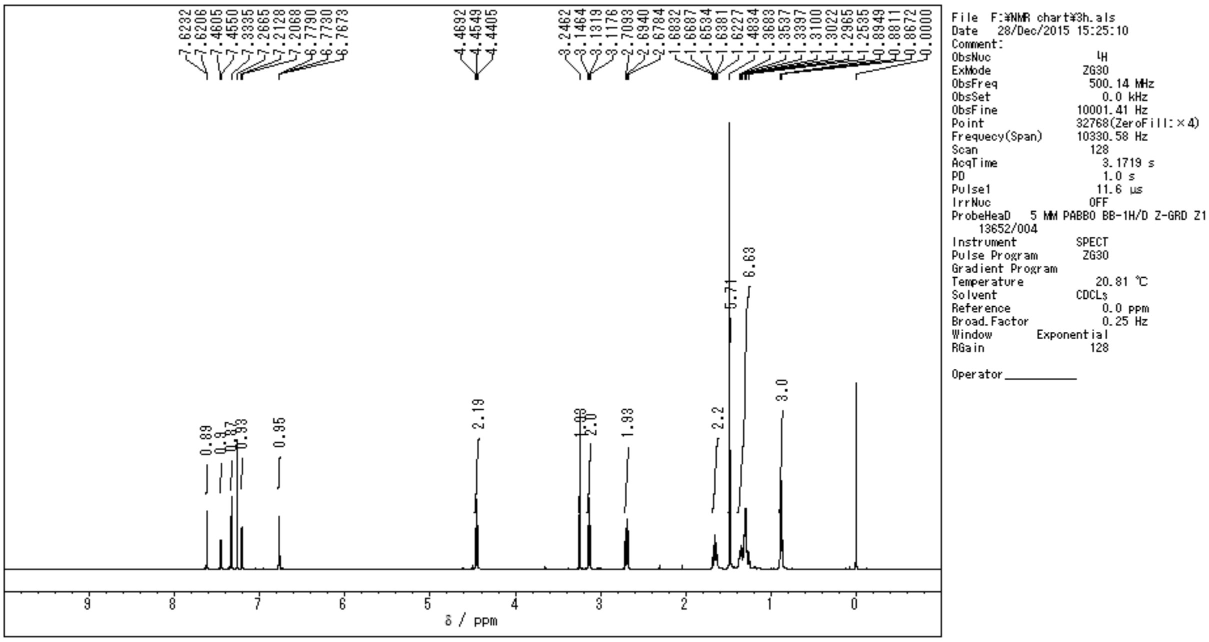

(^{13}C NMR, 125 MHz in CDCl$_3$)

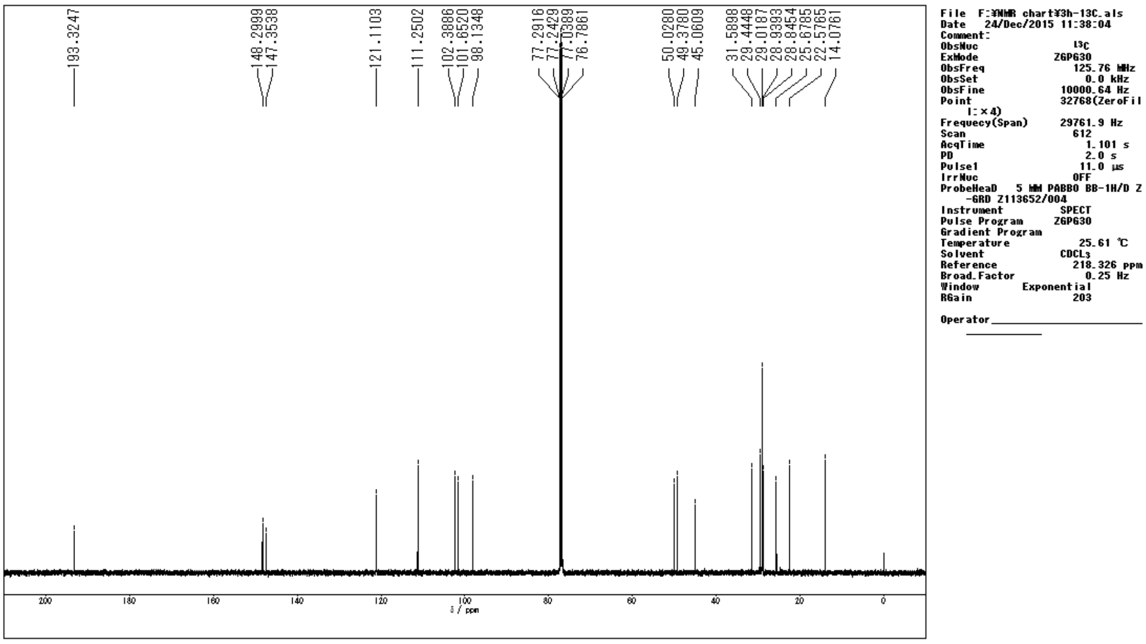

Supplementary Figure 32 | ^{1}H and ^{13}C NMR spectra of Compound 3h.

Compound **3i**

(^{1}H NMR, 500 MHz in $CDCl_3$)

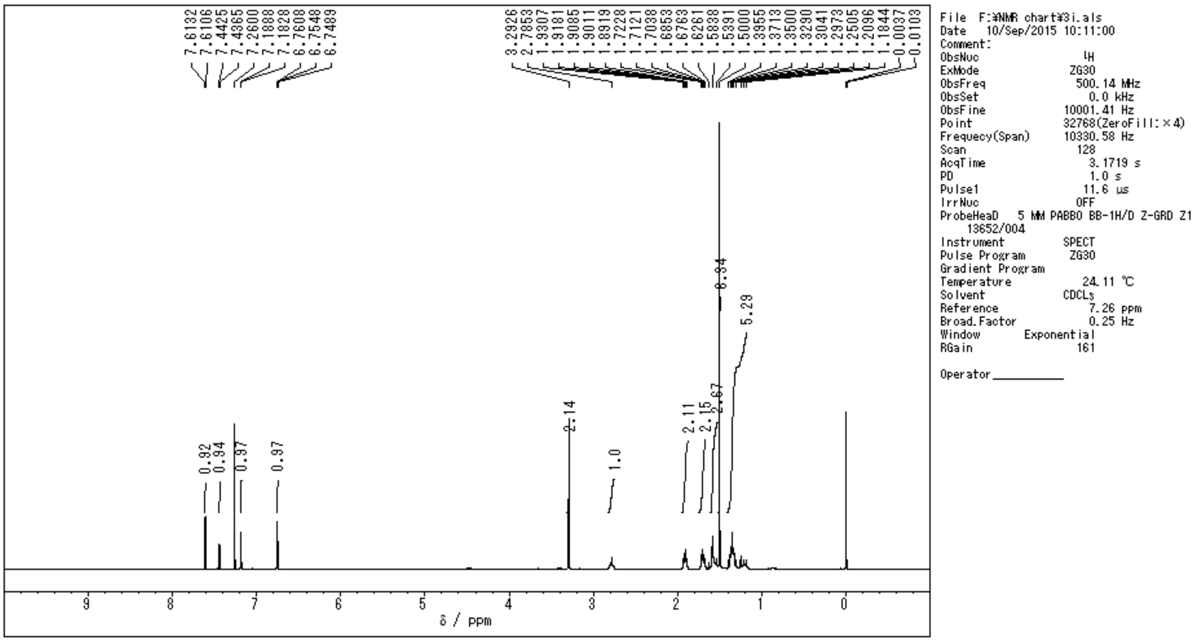

(^{13}C NMR, 100 MHz in $CDCl_3$)

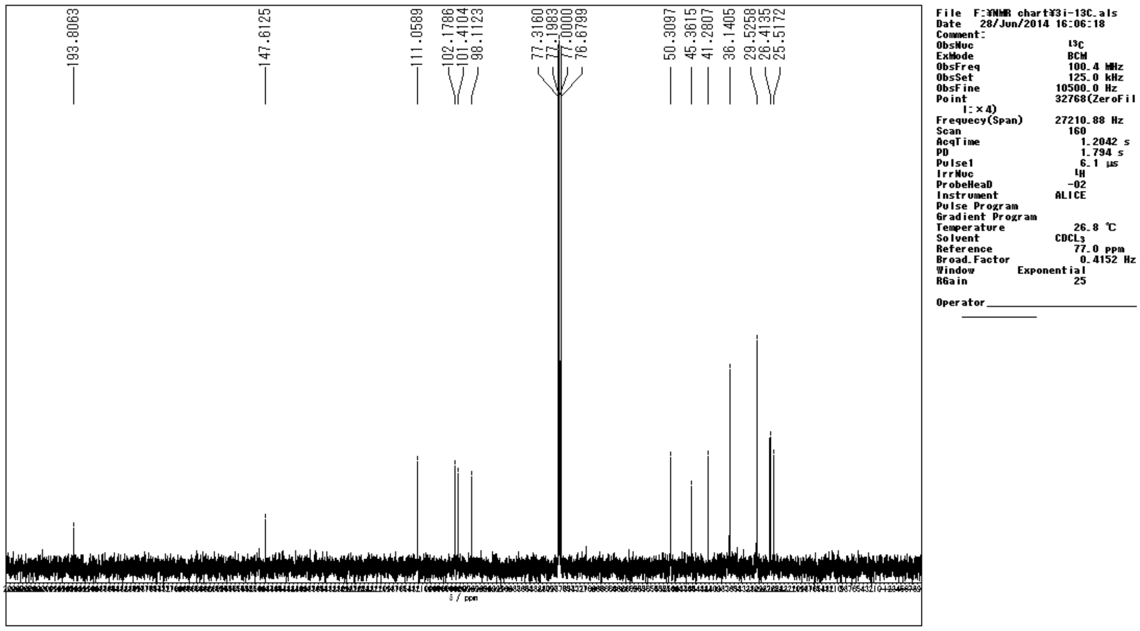

Supplementary Figure 33 | ^{1}H and ^{13}C NMR spectra of Compound 3i.

Compound **3j**

(^{1}H NMR, 400 MHz in $CDCl_3$)

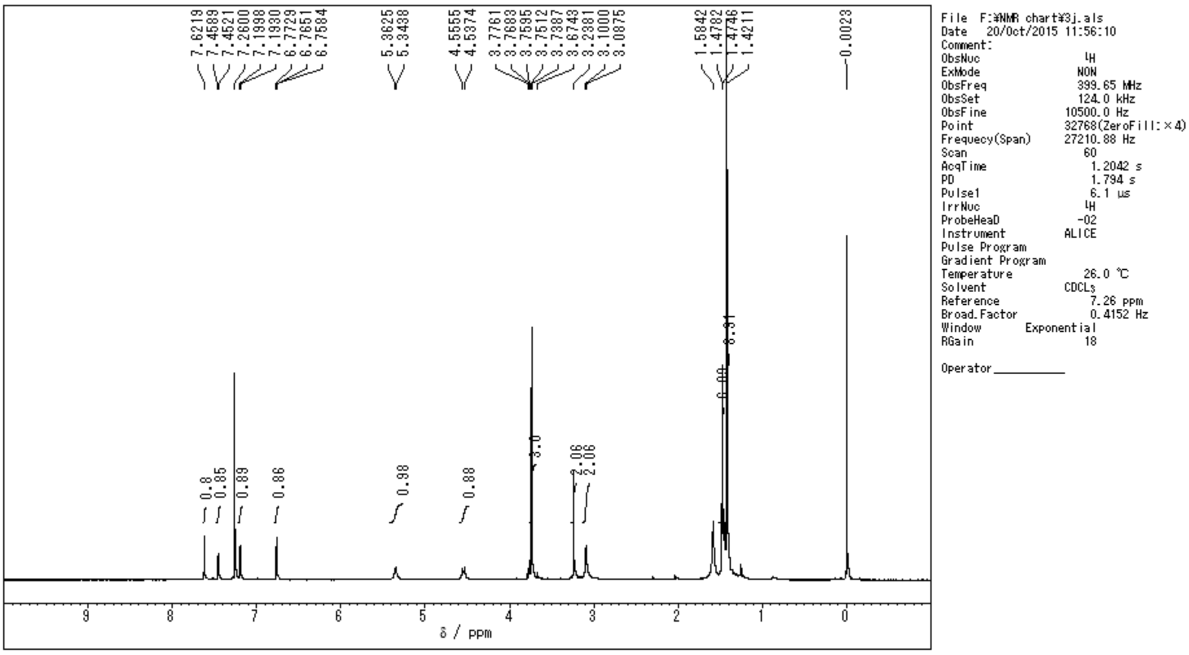

(^{13}C NMR, 125 MHz in $CDCl_3$)

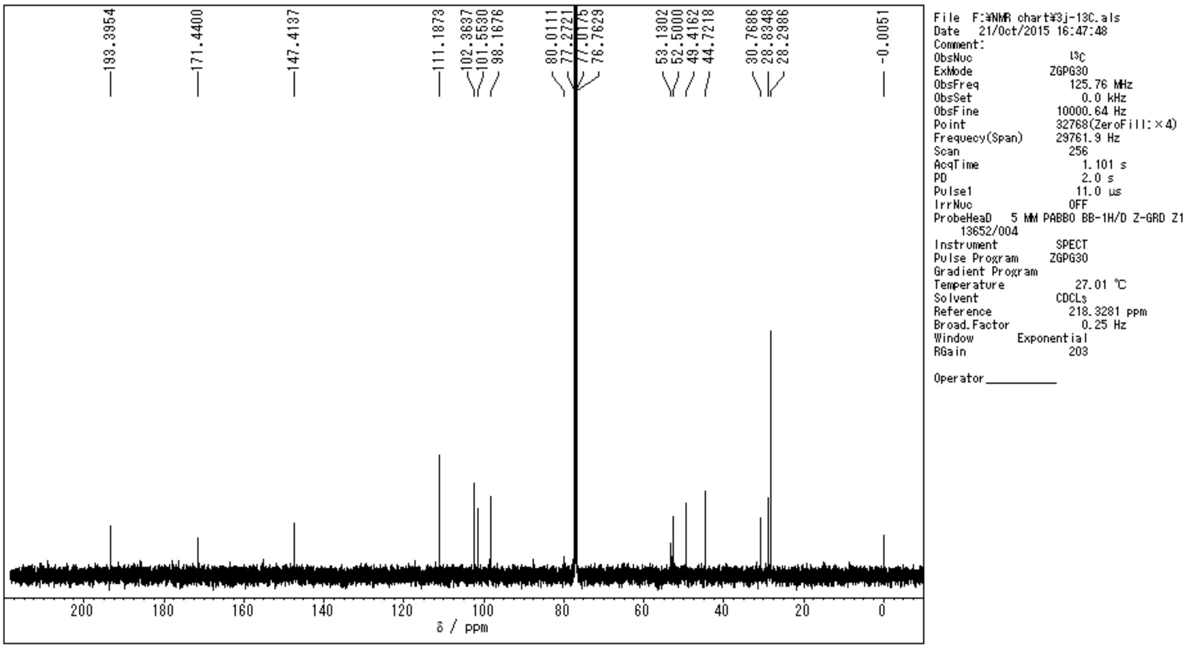

Supplementary Figure 34 | ^{1}H and ^{13}C NMR spectra of Compound 3j.

Compound **3k**

(^{1}H NMR, 400 MHz in $CDCl_3$)

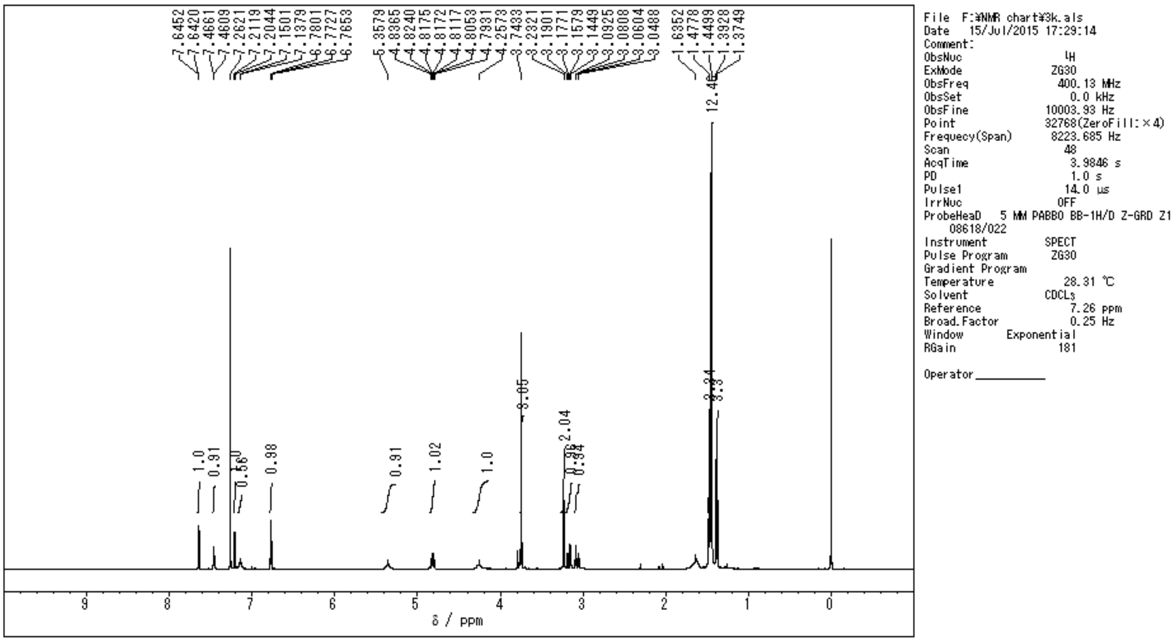

(^{13}C NMR, 100 MHz in $CDCl_3$)

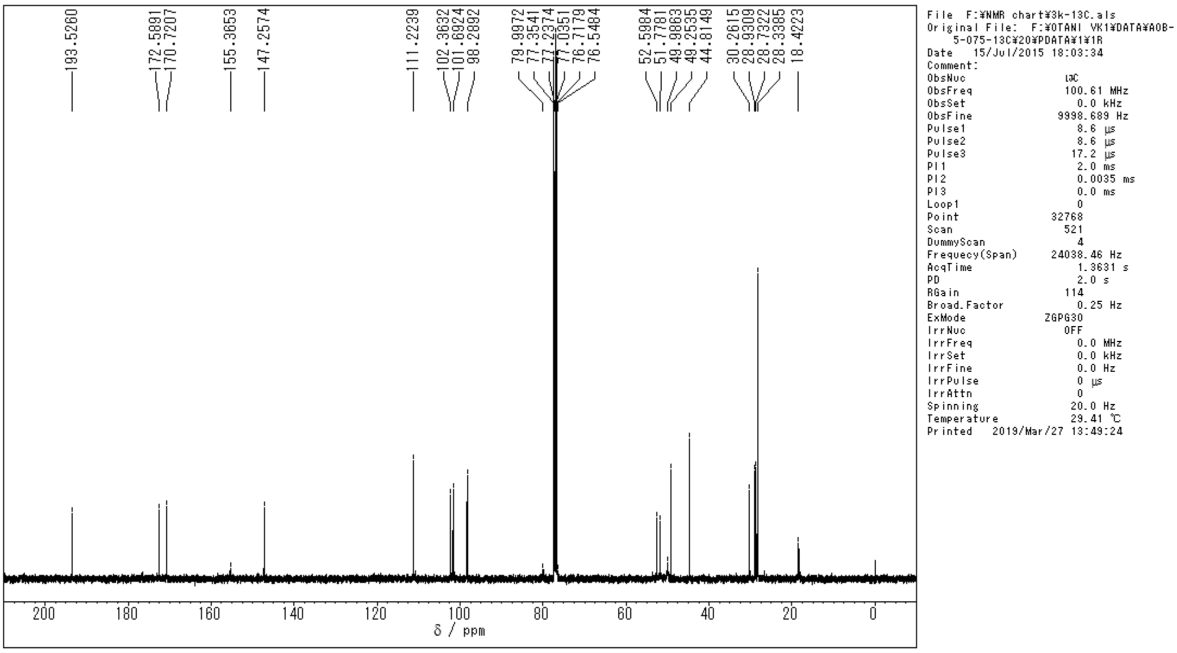

Supplementary Figure 35 | ^{1}H and ^{13}C NMR spectra of Compound 3k.

Compound **3l**

(^{1}H NMR, 500 MHz in D_2O)

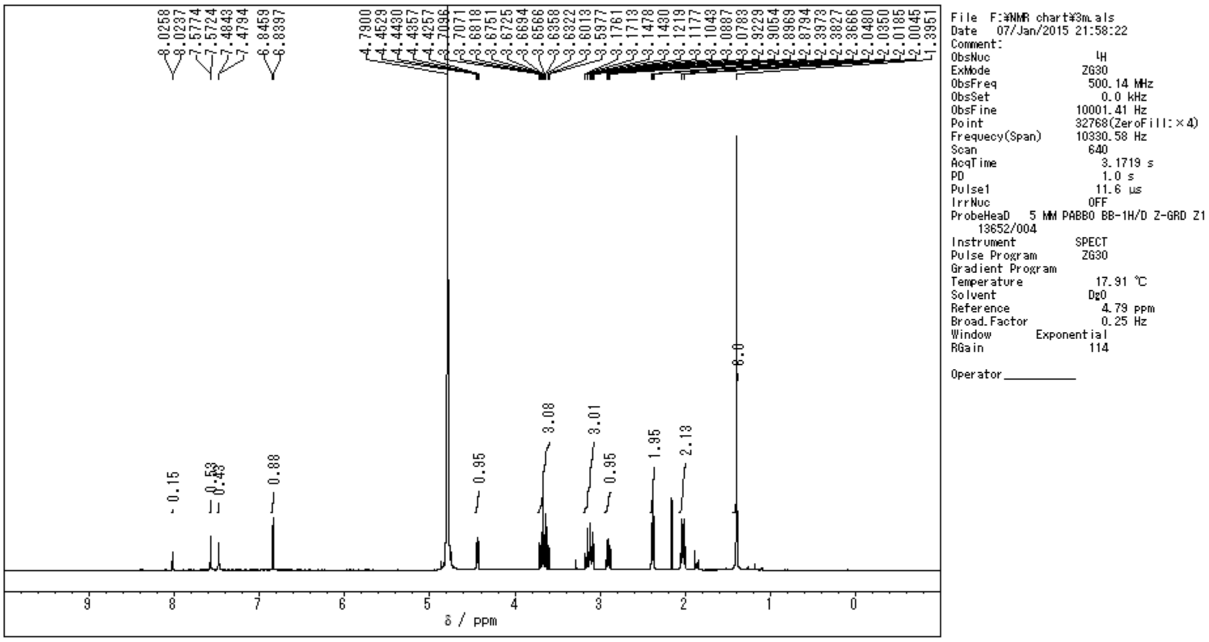

(^{13}C NMR, 125 MHz in D_2O)

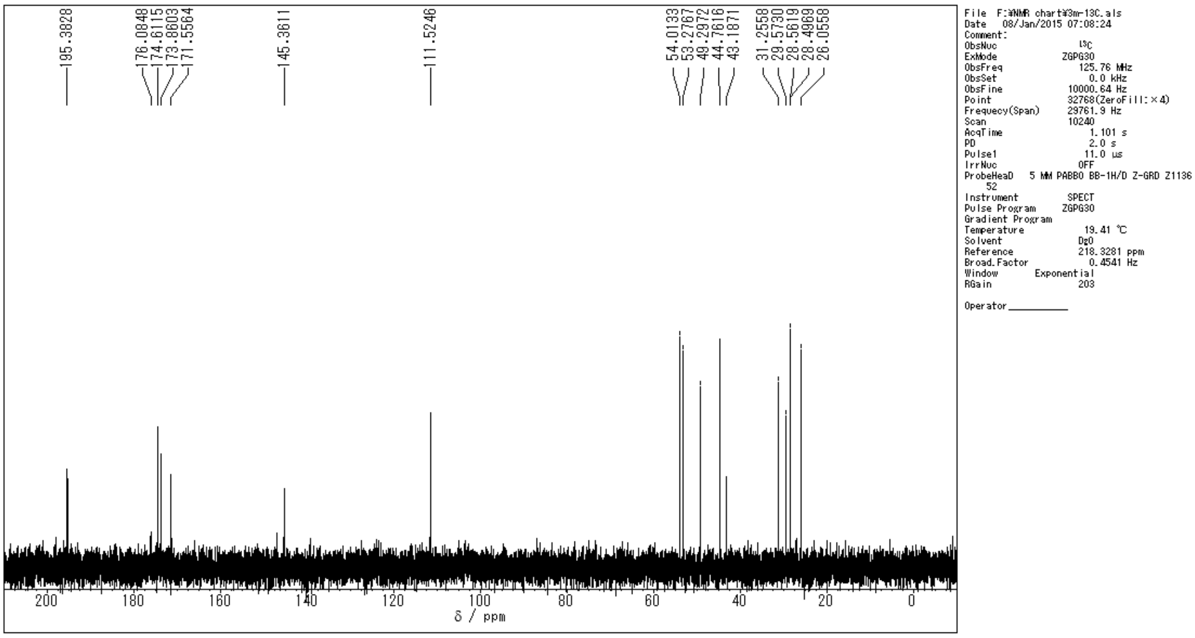

Supplementary Figure 36 | ^{1}H and ^{13}C NMR spectra of Compound 3l.

Compound **3o**

(^{1}H NMR, 500 MHz in $CDCl_3$)

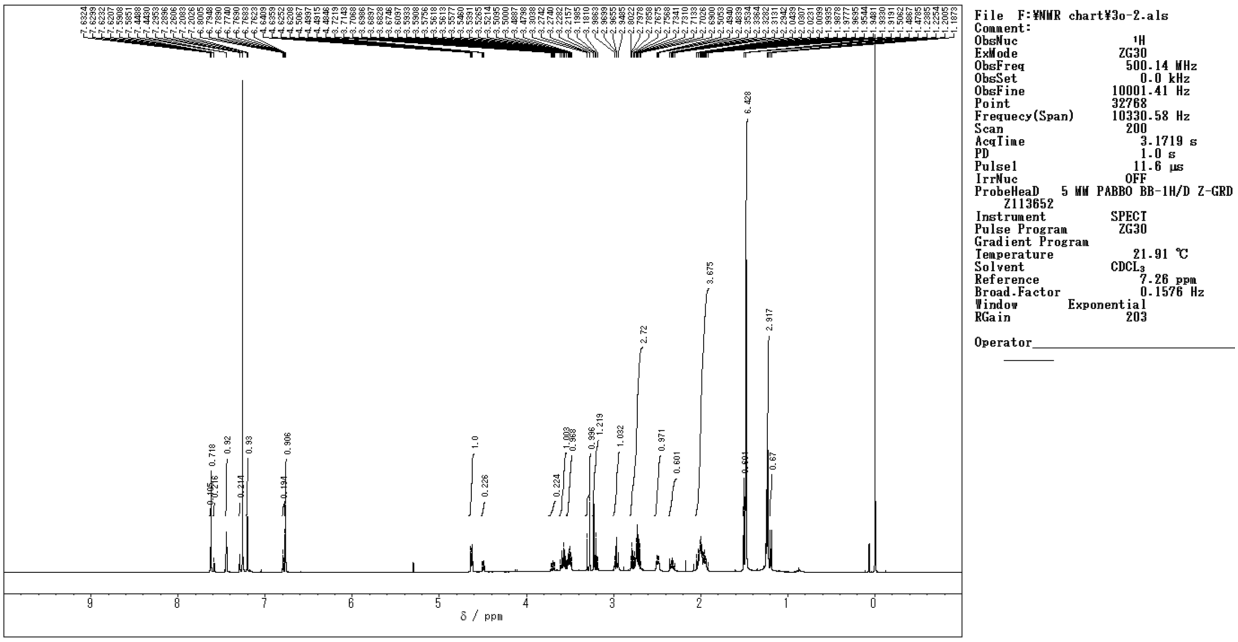

(^{13}C NMR, 125 MHz in $CDCl_3$)

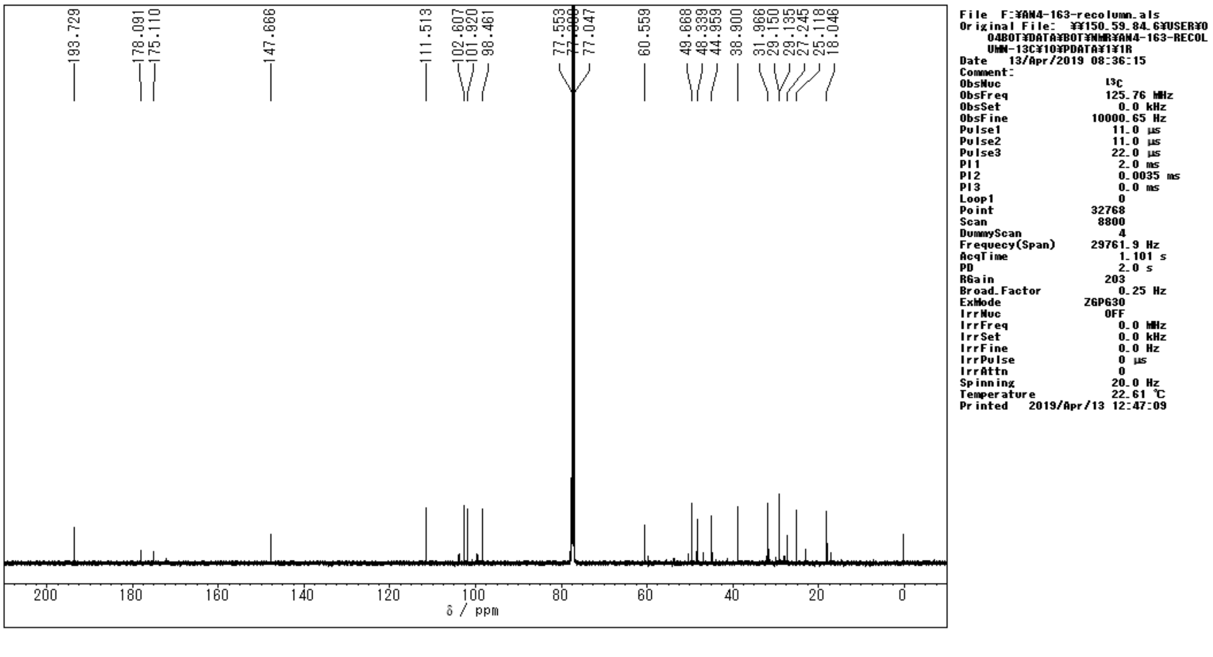

Supplementary Figure 37 | ^{1}H and ^{13}C NMR spectra of Compound 3o.

Compound **1a:** Ex/Em = 285 nm/294 nm

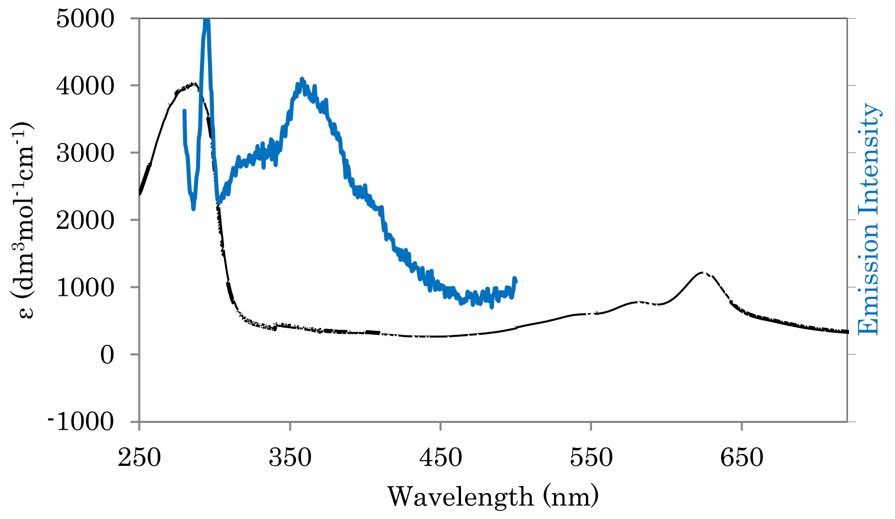

Supplementary Figure 38 | Absorption and Fluorescence emission spectra of 1a.

.

Compound **1b:** Ex/Em = 327 nm/420 nm

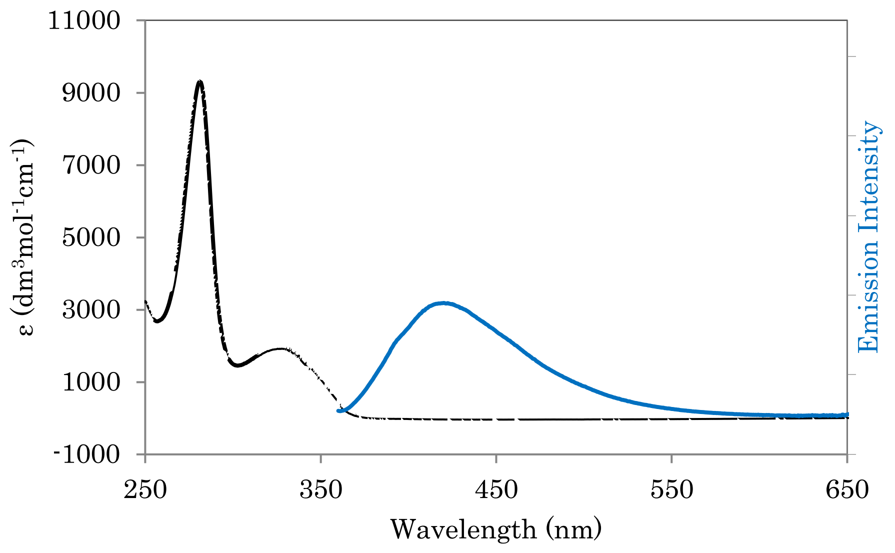

Supplementary Figure 39 | Absorption and Fluorescence emission spectra of 1b.

Compound **1c** Ex/Em = 344 nm/442 nm

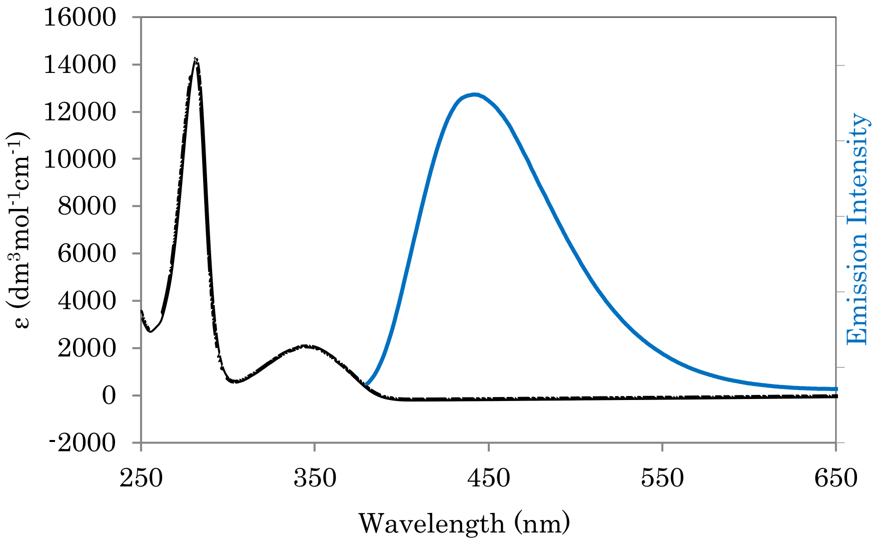

Supplementary Figure 40 | Absorption and Fluorescence emission spectra of 1c.

Compound **1d** Ex/Em = 381 nm/515 nm

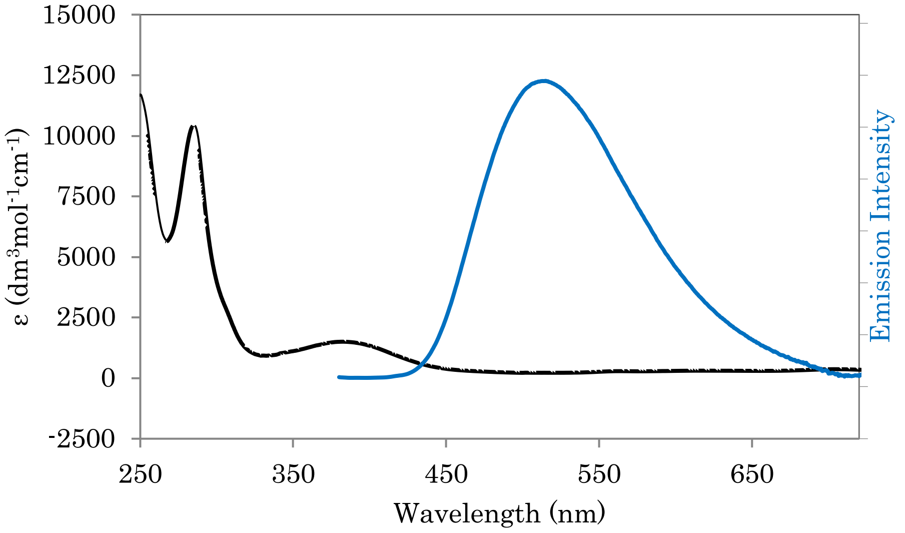

Supplementary Figure 41 | Absorption and Fluorescence emission spectra of 1d.

Compound **5** Ex/Em = 331 nm/432 nm

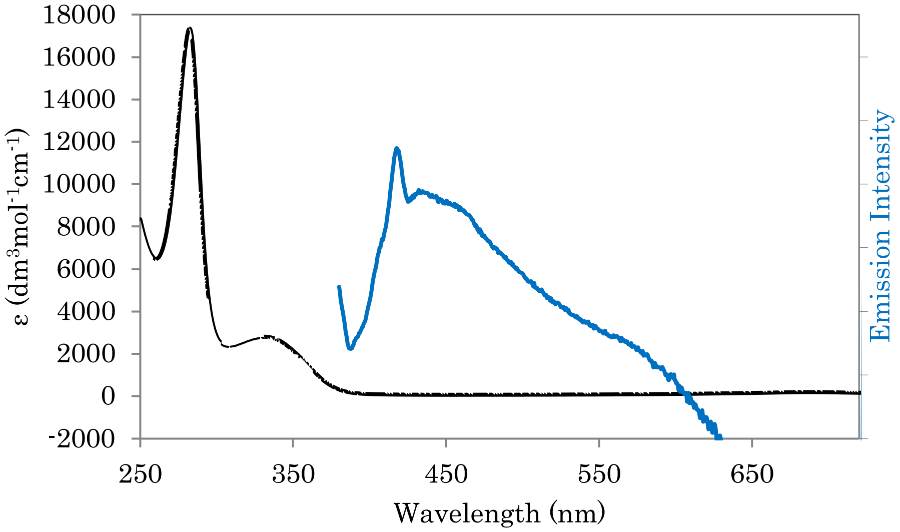

Supplementary Figure 42 | Absorption and Fluorescence emission spectra of 5.

Compound **2a:** Ex/Em = 409 nm/574 nm

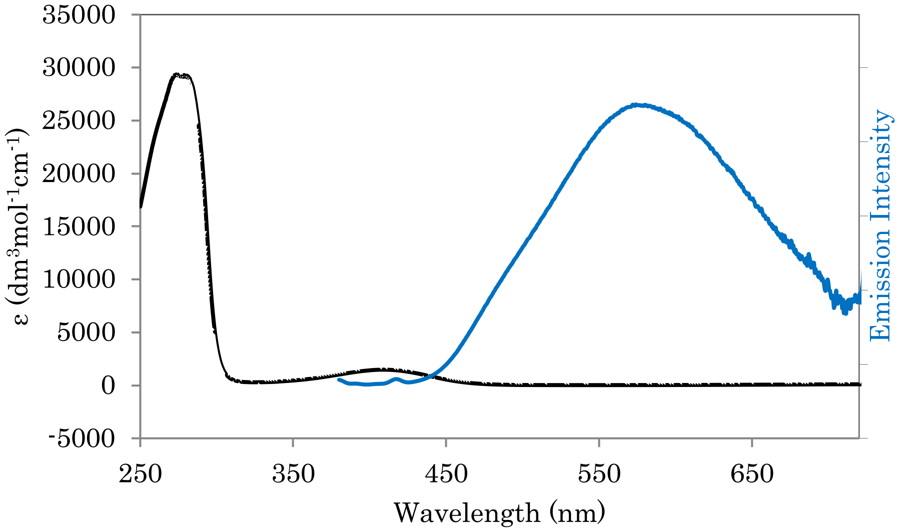

Supplementary Figure 43 | Absorption and Fluorescence emission spectra of 2a.

Compound **3a:** Ex/Em = 389 nm/521 nm

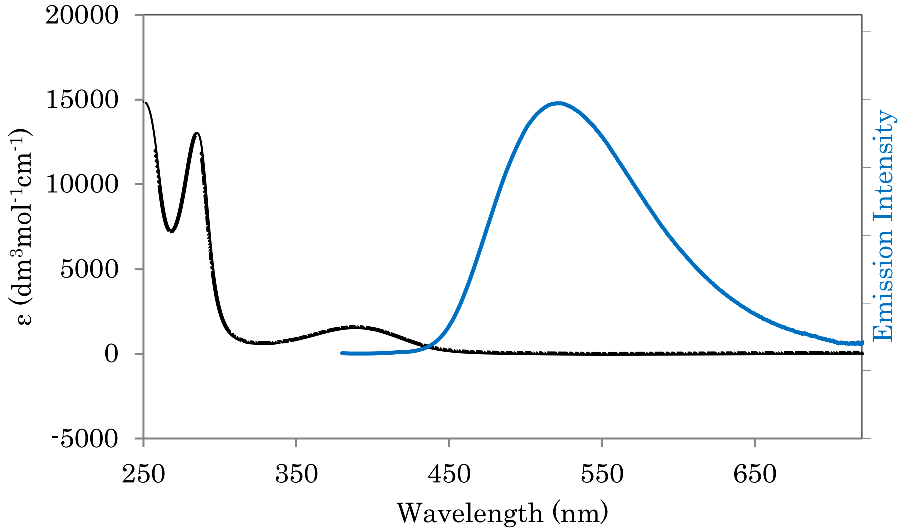

Supplementary Figure 44 | Absorption and Fluorescence emission spectra of 3a.

Compound **3b:** Ex/Em = 388 nm/519 nm

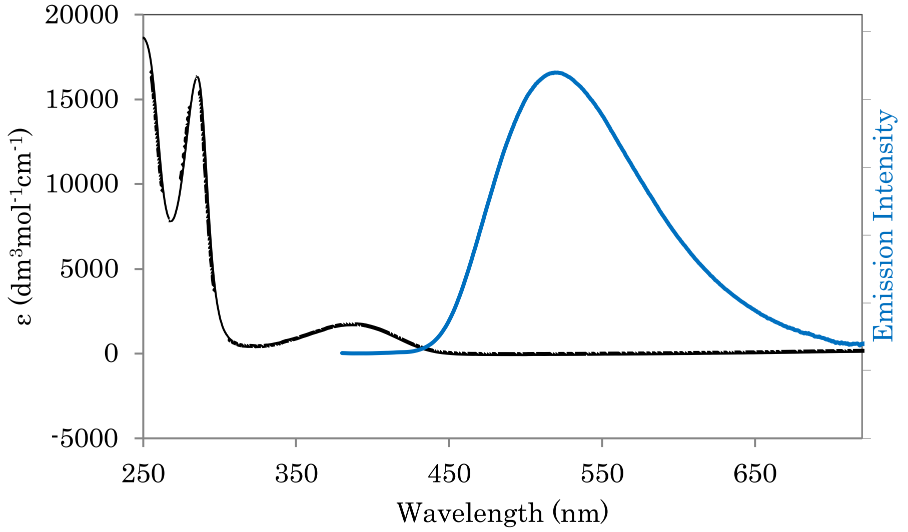

Supplementary Figure 45 | Absorption and Fluorescence emission spectra of 3b.

Compound **3c:** Ex/Em = 388 nm/525 nm

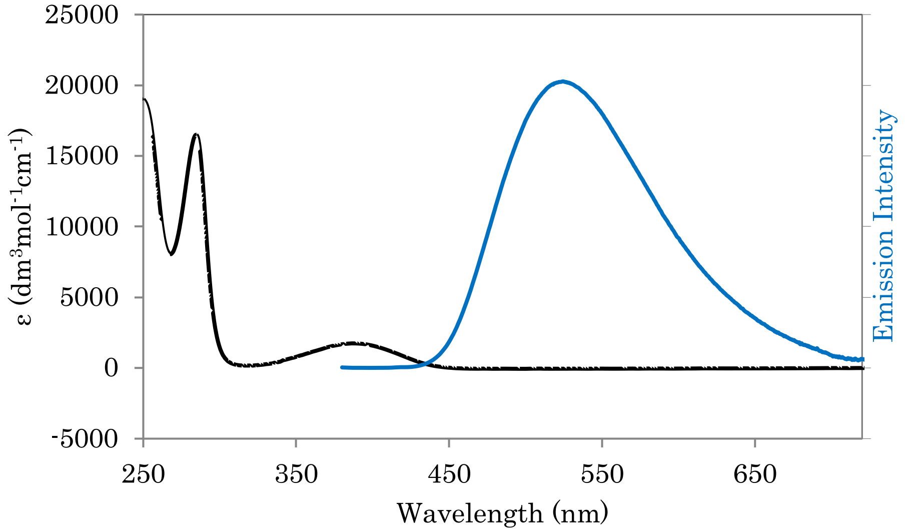

Supplementary Figure 46 | Absorption and Fluorescence emission spectra of 3c.

Compound 3d: Ex/Em =389 nm/517 nm

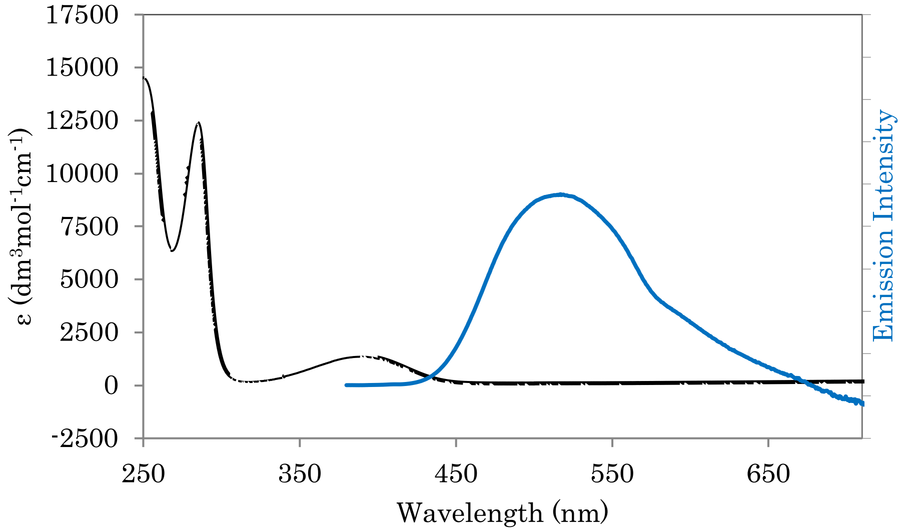

Supplementary Figure 47 | Absorption and Fluorescence emission spectra of 3d.

Compound **3e:** Ex/Em = 390 nm/536 nm

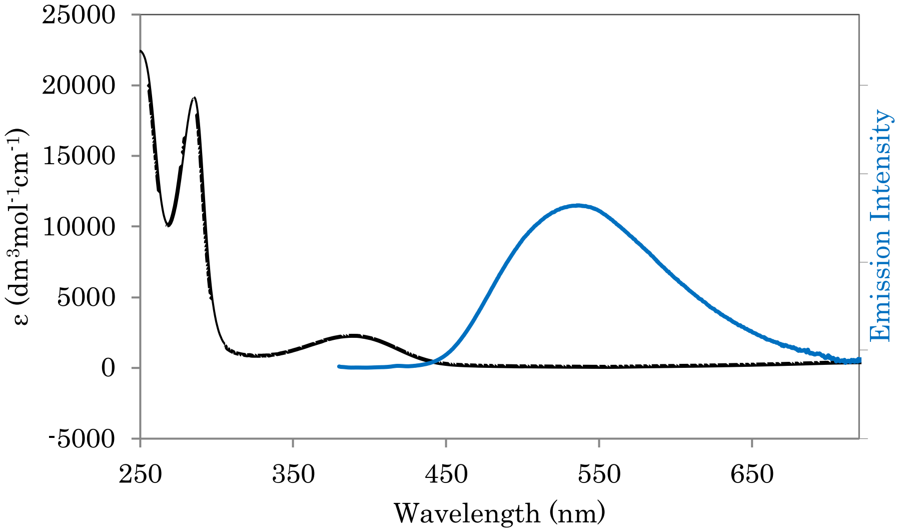

Supplementary Figure 48 | Absorption and Fluorescence emission spectra of 3e.

Compound **3f:** Ex/Em = 391 nm/532 nm

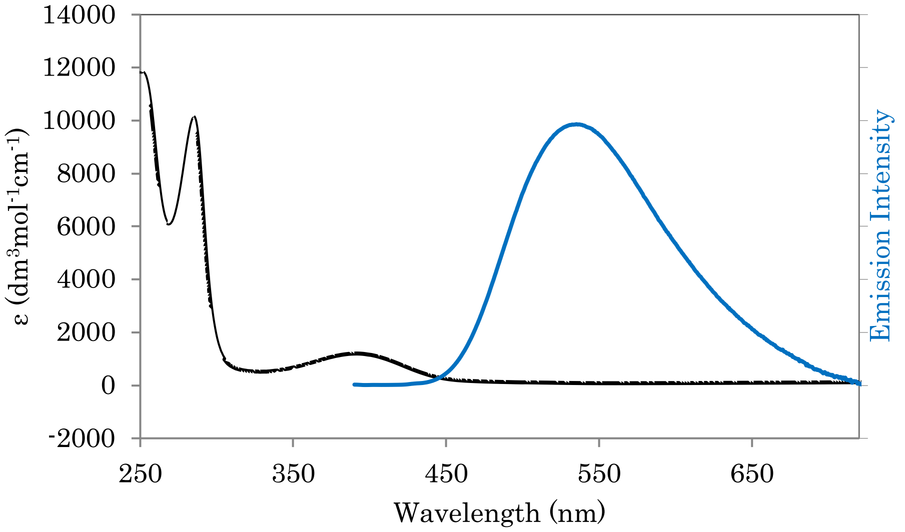

Supplementary Figure 49 | Absorption and Fluorescence emission spectra of 3f.

Compound **3g:** Ex/Em = 360 nm/471 nm

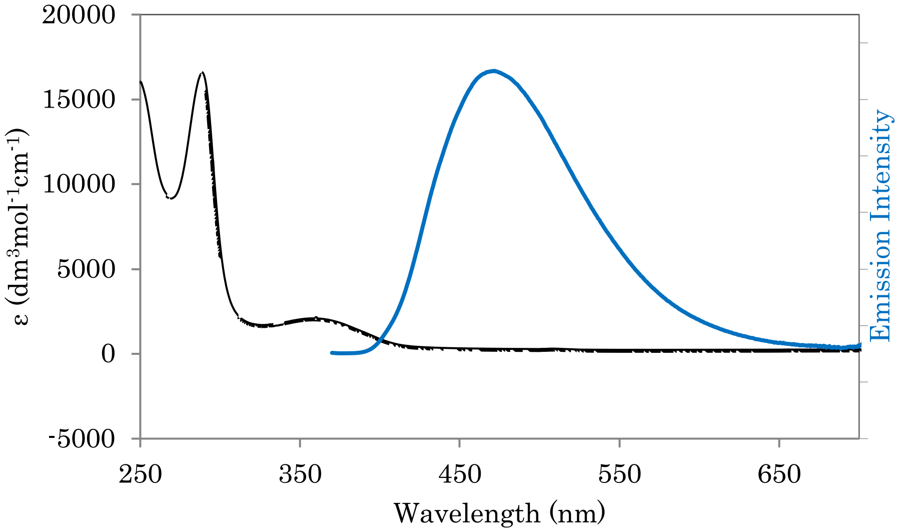

Supplementary Figure 50 | Absorption and Fluorescence emission spectra of 3g.

Compound **3h:** Ex/Em = 392 nm/520 nm

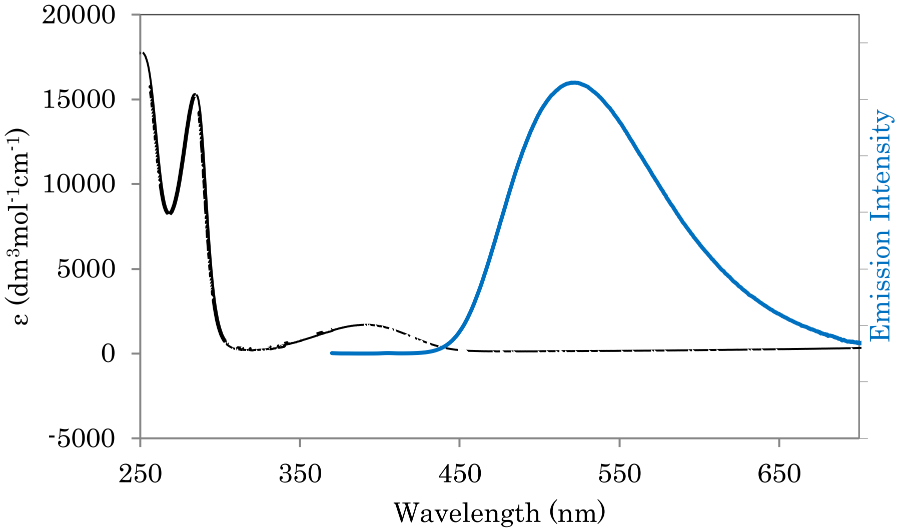

Supplementary Figure 51 | Absorption and Fluorescence emission spectra of 3h.

Compound **3i:** Ex/Em = 389 nm/521 nm

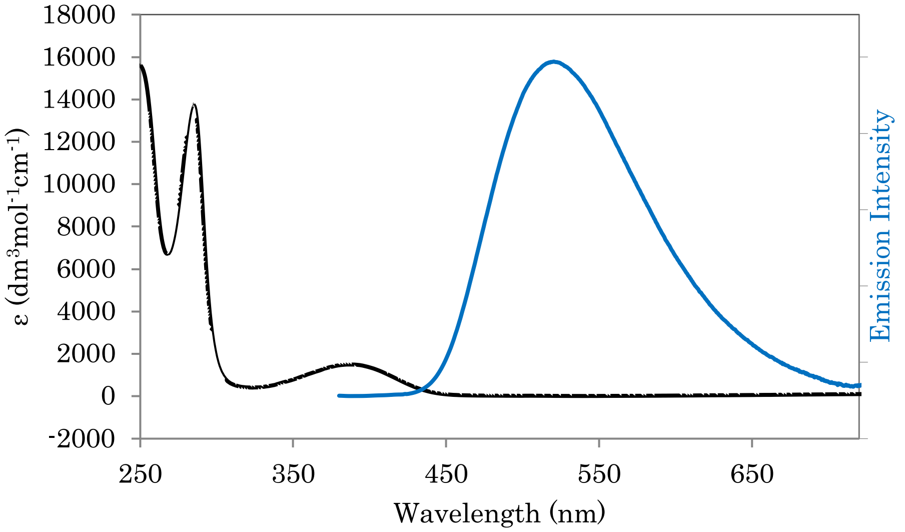

Supplementary Figure 52 | Absorption and Fluorescence emission spectra of 3i.

Compound **3j:** Ex/Em = 390 nm/521 nm

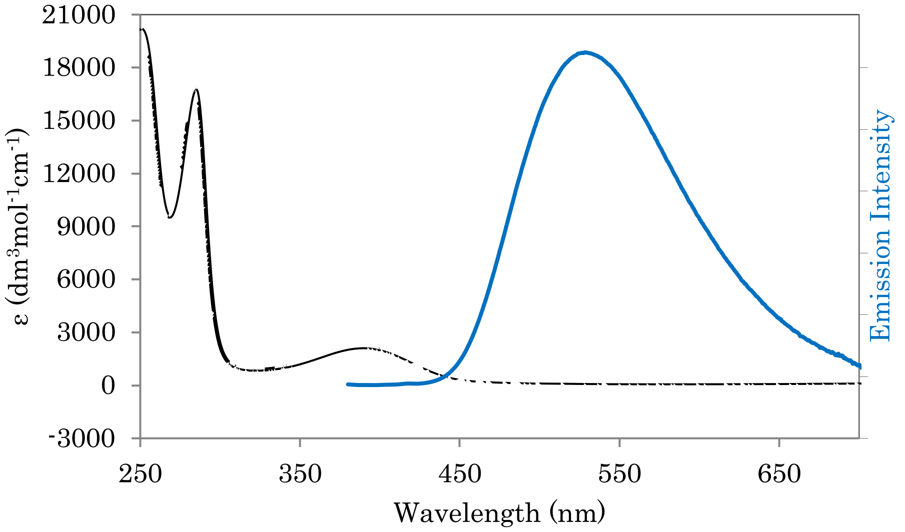

Supplementary Figure 53 | Absorption and Fluorescence emission spectra of 3j.

Compound **3k:** Ex/Em = 393 nm/540 nm

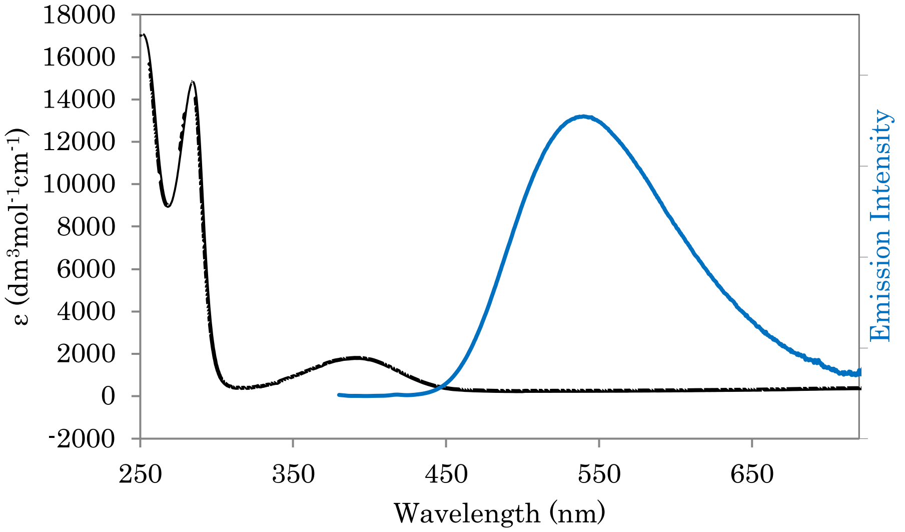

Supplementary Figure 54 | Absorption and Fluorescence emission spectra of 3k.

Compound **3l:** Ex/Em = 396 nm/493 nm

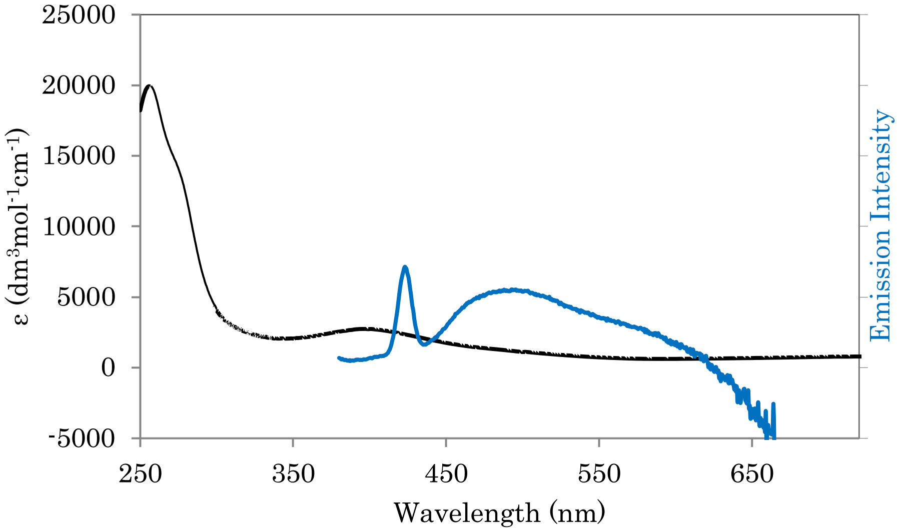

Supplementary Figure 55 | Absorption and Fluorescence emission spectra of 3l.

Compound **3m:** Ex/Em =401 nm/508 nm

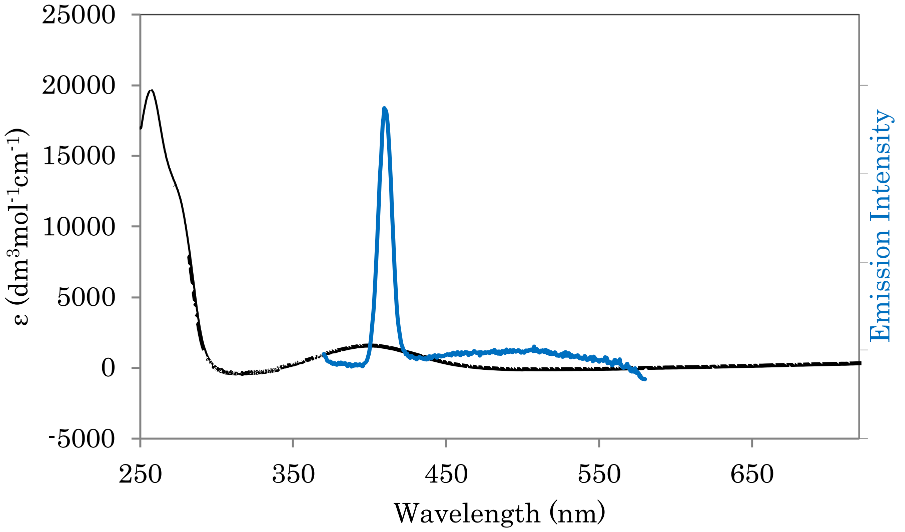

Supplementary Figure 56 | Absorption and Fluorescence emission spectra of 3m.

Compound **3o:** Ex/Em = 395 nm/506 nm

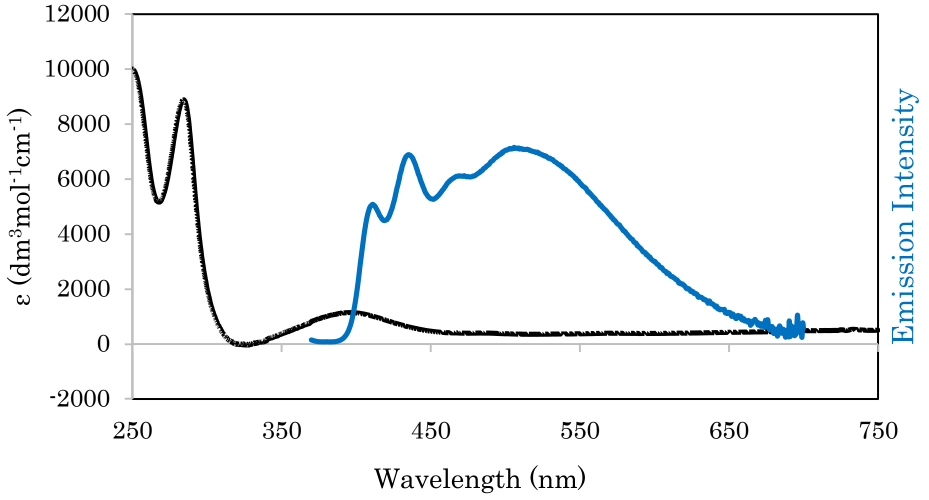

Supplementary Figure 57 | Absorption and Fluorescence emission spectra of 3o.

· Supplementary References

[1] Kamada, R.; Tano, F.; Kudoh, F.; Kimura, N.; Chuman, Y.; Osawa, A.; Namba, K.; Tanino, K.; Sakaguchi, K.; *PLoS ONE* **11**, e0160625 (2016).

[2] Namba, K.; Osawa, A.; Nakayama, A.; Mera, A.; Tano, F.; Chuman, Y.; Sakuda, E.; Taketsugu, T.; Sakaguchi, K.; Kitamura, N.; Tanino. K. *Chem. Sci.* **6**, 1083-1093 (2015).
